# Acupuncture-related therapies for post-stroke pain management: a scoping review and evidence map

**DOI:** 10.3389/fneur.2025.1604655

**Published:** 2025-08-06

**Authors:** Zhuo Zhou, Chao Ke, Wenying Shi, Zhengrong Xie, Zeli Hu, Yilin Zhou, Wei Zhang

**Affiliations:** The First Hospital of Hunan University of Chinese Medicine, Changsha, China

**Keywords:** post-stroke pain, acupuncture therapies, moxibustion, complementary and alternative therapies, review, evidence mapping

## Abstract

**Purpose:**

Post-stroke pain (PSP) is a common symptom among patients with stroke, and acupuncture-related therapies can provide pain relief. This study aimed to summarize the current status of research by mapping the evidence from clinical research on acupuncture therapies for PSP, to identify existing gaps, and to provide a foundation for future research.

**Methods:**

A systematic search was conducted of eight databases for randomized controlled trials (RCTs) and systematic reviews/meta-analyses (SRs/MAs), from database inception to October 22, 2024. The characteristics of the RCTs, including publication profiles, study populations, intervention protocols, and outcome measures, were analyzed and explained with a combination of text and graphics. The methodological quality of RCTs and SRs/MAs was evaluated using the risk of bias (ROB) and AMSTAR2 checklists, respectively.

**Results:**

A total of 346 studies (339 RCTs and 7 SRs/Mas) were included in the evidence map. The earliest study was published in 1994 and the number of publications peaked in 2021. Most studies were published in China, with a limited number of studies published in English. The majority of the studies were conducted on patients in the recovery phase, with shoulder pain being the most frequently reported condition. The most widely used study design compared a combined acupuncture with rehabilitation intervention with rehabilitation alone. The most frequently used interventions were body acupuncture, electroacupuncture, scalp acupuncture, moxibustion, and warm needling. The most frequently used acupoints were Jianyu (LI15), Jianliao (SJ14), and Quchi (LI11). The most frequently involved meridians were the Stomach Meridian of Foot-Yangming (LI) and Triple Energizer Meridian of Hand-Shaoyang (TE). The forearm-upper arm region and the Eight Confluent Points-Luo-Connecting Points were the most frequently targeted area and specific acupoint, respectively. Outcome measures primarily focused on pain relief. The systematic reviews confirmed the effectiveness of acupuncture for PSP.

**Conclusion:**

Acupuncture-related therapies are effective interventions for PSP relief. However, the overall research quality was low, with large evidence gaps. To promote the evidence-based practice, future studies should implement strict inclusion and exclusion criteria and standardize research procedures to ensure high quality and methodological rigor of systematic reviews.

## 1 Introduction

Stroke ranks as the second most prevalent disease worldwide with high rates of mortality and disability (1), and the incidence in younger age groups is increasing. By 2030, the prevalence is projected to rise to 3.9%, a 20.5% increase compared with the prevalence in 2012 (2). The sequelae associated with stroke place substantial psychological and financial burdens on survivors. Post-stroke pain (PSP) is a form of neuropathic pain arising from central nervous system alterations due to stroke, such as central sensitization (3), neuroplastic changes (4), reduced inhibitory mechanisms (5), and localized brain inflammation (4). Its main subtypes include central PSP, hemiplegic shoulder pain, spasticity-induced pain, headache, and complex regional pain syndrome (CRPS) (6, 7). Approximately 30% of patients experience pain in the early stages after a stroke (8), and as the condition progresses, the prevalence of pain increases, accompanied by increased severity and higher rates of depression, fatigue, and anxiety (9). Therefore, effectively alleviating pain is crucial for improving patients’ quality of life and mental health.

Owing to the complex mechanisms underlying various types of PSP, its treatment remains challenging, requiring continuous research and innovation in treatment methods. Current pharmacological treatments focus mainly on drugs targeting the primary lesion and analgesics, antispasmodics, anticonvulsants (e.g., gabapentin and pregabalin), and opioid analgesics (10, 11). Non-drug treatments consist mainly of surgical procedures such as nerve blocks and deep brain stimulation or motor cortex stimulation, alongside psychological therapies (12). Nevertheless, drugs have severe side effects and a high risk of developing tolerance, whereas surgical treatments pose a risk of complications such as bleeding, infection, and hemiplegia (12, 13). Acupuncture, as a traditional Chinese medicine (TCM), is recommended by multiple guidelines for the treatment of post-stroke sequelae, particularly for PSP (14). A meta-analysis has shown that non-pharmacological interventions can effectively relieve pain immediately after the intervention, with acupuncture being particularly effective (15).

Evidence mapping is a novel approach to evidence synthesis (16). It provides an innovative and visual approach summarizing the findings of systematic reviews and overviews of systematic reviews, presenting the impact on relevant research fields in an intuitive manner (16–18). Numerous studies have reviewed research on TCM interventions for different diseases (19–21). However, to our knowledge, no research has specifically focused on evidence mapping for acupuncture-related therapies in managing PSP. Thus, we built an evidence map using existing clinical research, to uncover gaps and inconsistencies in the current evidence base, define future research directions, and provide an evidence base for scientific decision-making by governments, researchers, practitioners, and other stakeholders.

## 2 Methods

### 2.1 Search strategy

We systematically searched eight databases, including the China National Knowledge Network (CNKI), Wanfang Database, China BioMedical Database (CBM), Chongqing VIP Database (VIP), PubMed, Web of Science (WOS), EMBASE and the Cochrane Library, for Chinese and English studies on clinical randomized controlled trials (RCTs) and systematic reviews/meta-analyses (SRs/MAs) of acupuncture-related therapies for PSP published from the inception of each database to October 22, 2024. The search strategy incorporated key terms and their potential variations, such as “stroke,” “pain” and “acupuncture,” using both subject headings and free-text words. The complete search strategies and results are provided in Supplementary Appendix 1.

### 2.2 Eligibility criteria

#### 2.2.1 Study types

This study included all original clinical RCTs, systematic reviews, and meta-analyses focusing on acupuncture for PSP. If the same research team produced multiple systematic reviews addressing the same clinical issue, only the latest version was included. Critical reviews, conference papers, scoping reviews, rapid reviews, and narrative reviews were excluded.

#### 2.2.2 Study participants

Participants were required to have a confirmed diagnosis of stroke supported by imaging data or according to established guidelines. All participants were required to have PSP, with no limitations on the type of pain, which could include central PSP, post-stroke shoulder pain (PHS), limb pain, headache, and CRPS, among other types. Patients with other concurrent diseases or symptoms were excluded.

#### 2.2.3 Interventions and control design

The intervention group was treated with acupuncture-related therapies, such as manual acupuncture, electroacupuncture (EA), scalp acupuncture, moxibustion, acupoint application, acupoint injection, auricular acupressure, and specialized acupuncture methods. These therapies could be used alone or in combination with other acupuncture or non-acupuncture therapies. The control group included various therapies such as no treatment, sham acupuncture, different types of acupuncture therapies, medications, and rehabilitation. If both groups received treatments other than acupuncture, these treatments had to be consistent, and studies where acupuncture was not the sole variable were excluded.

#### 2.2.4 Outcome measures

No predefined standards exist for outcome measures; therefore, all outcome indicators from each study were included in the evidence map.

### 2.3 Literature screening and data extraction

Two reviewers (ZZ and CK) independently conducted the preliminary literature searches across eight databases, and the retrieved literature was imported into EndNote X9 software (Clarivate, Philadelphia, USA), and duplicates were removed using a combination of EndNote X9 software and manual methods. Subsequently, preliminary screening was performed based on the titles and abstracts of the articles. For the remaining eligible literature, full texts were downloaded, and after reviewing the full texts, all studies meeting the eligibility criteria were identified, and relevant data were extracted.

Data extraction and management were performed independently by two reviewers (ZH and YZ). The extracted information, including literature titles, authors, journal names, publication dates, sample sizes, inclusion criteria, exclusion criteria, intervention measures, control measures, diagnostic criteria, treatment courses, and outcome indicators was entered into Microsoft Excel 2024 (Microsoft Corp., Redmond, WA, USA). Data standardization was performed according to the criteria outlined in Supplementary Appendix 2. Disagreements during the study were resolved through discussion or consultation with a third reviewer (WZ) until a consensus was achieved.

### 2.4 Data analysis and evidence presentation

Following the extraction of information from all included studies, data analysis was performed. The results of the data analysis were presented as textual descriptions accompanied by charts, including tables, line graphs, bar charts, pie charts, and bubble charts. All statistical analyses were conducted using Microsoft Excel 2024, and the figures were prepared using software such as Microsoft Excel 2024, Microsoft PowerPoint 2024, and Adobe Illustrator (Adobe, San Jose, USA). The Sankey diagram was prepared by ChiPlot,^1^ a comprehensive web service for biomedical data analysis and visualization. Acupoint association diagrams were generated using IBM SPSS Modeler 18.0 (IBM Corp., Armonk, NY, USA), and the acupoint map model was sourced from the FigDraw platform (approval number: OUIIY66727).^2^

### 2.5 Methodological quality assessment

Two reviewers (ZZ and CK) independently evaluated the methodological quality of the included RCTs using the Cochrane Collaboration risk of bias (RoB) tool (22). This RoB assessment tool covers seven primary items: random sequence generation, allocation concealment, blinding of participants and personnel, blinding of outcome assessment, completeness of outcome data, selective reporting of results, and other potential biases. Each item was rated as “low,” “high,” or “unclear” based on the criteria.

Similarly, the same two reviewers (ZZ and CK) used the assessing the methodological quality of systematic reviews (AMSTAR) 2 tool to assess the methodological quality of the included systematic reviews (SRs) (23). The AMSTAR 2 tool consists of 16 methodological domains, with each item classified as “yes,” “no,” or “partially.” Items 2, 4, 7, 9, 11, 13, and 15 were critical domains, and the overall confidence in the results of the SRs was rated as “high,” “moderate,” “low,” or “very low.”

## 3 Results

### 3.1 Literature search

**Figure 1:**
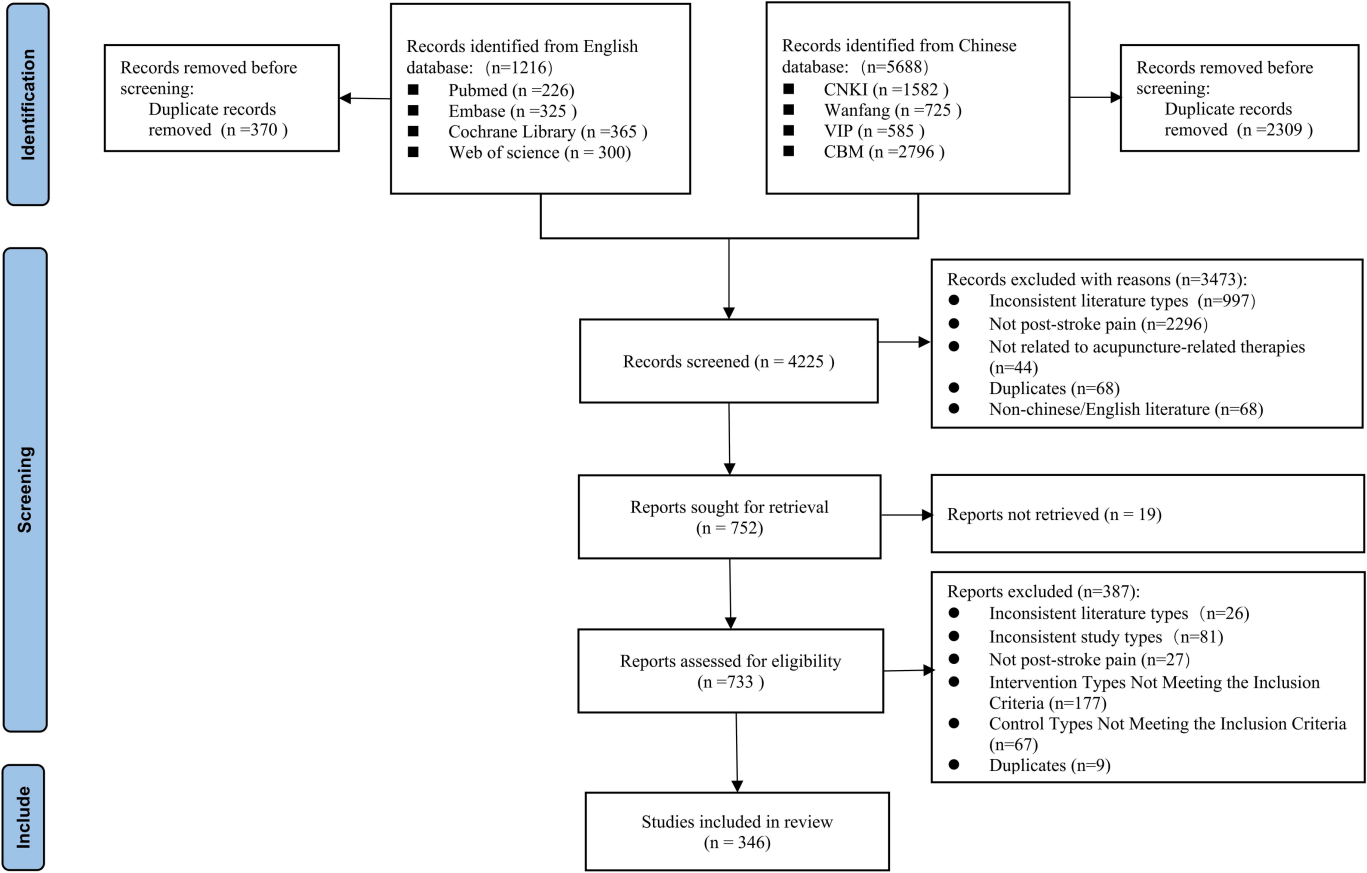
Flow diagram representing the process of literature screening.

An initial search of eight Chinese and English databases retrieved 6,904 articles, with 346 meeting the eligibility criteria and included in our evidence map analysis. The study selection process adhered to the Preferred Reporting Items for Systematic reviews and Meta-Analyses (PRISMA) guidelines (24), as shown in Figure 1. The full list of the included studies is provided in Supplementary Appendix 3.

### 3.2 Basic characteristics of evidence sources

**Figure 2:**
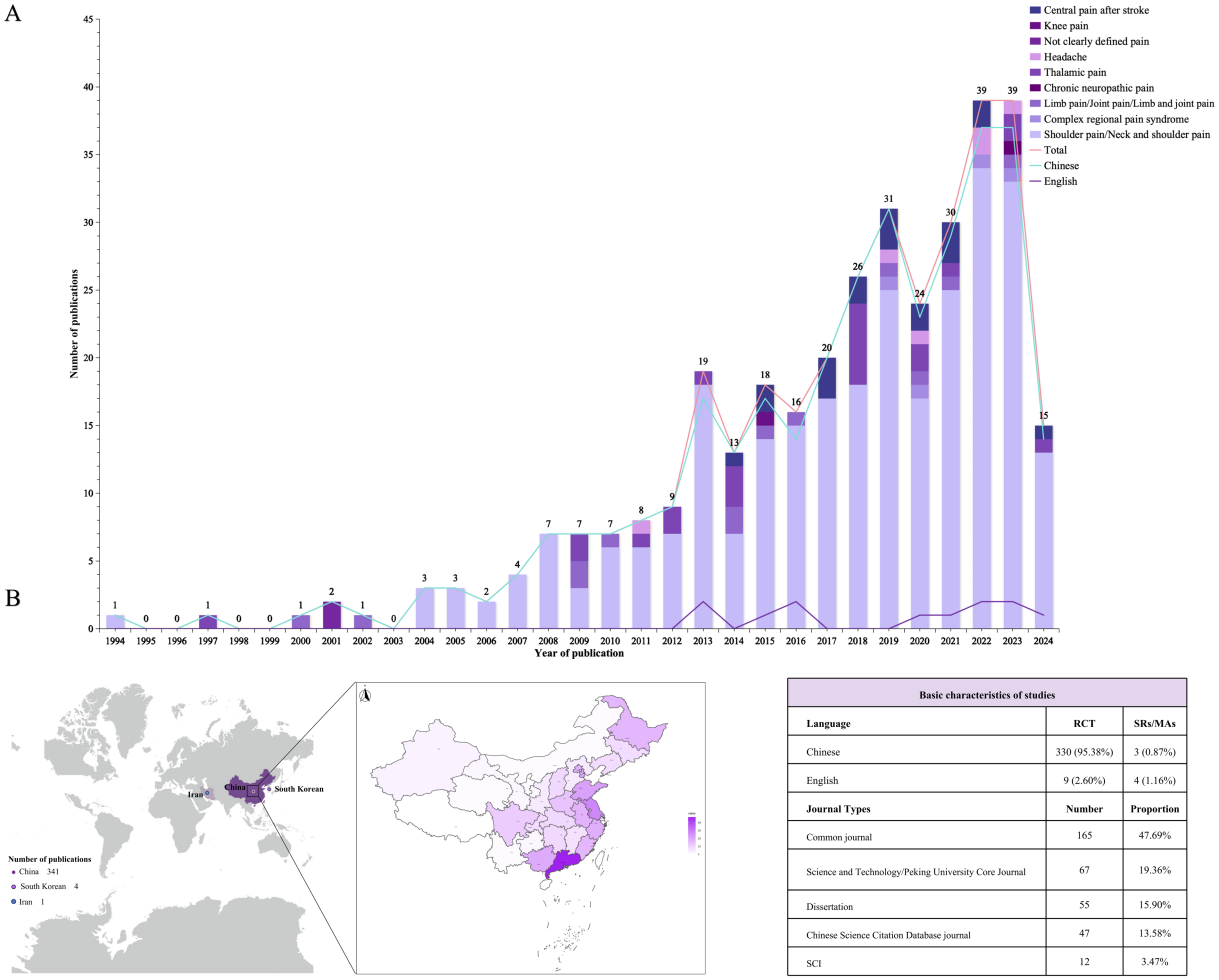
Basic characteristics of the sources of evidence. **(A)** Publication year chart. The line graphs represent the publication volumes of Chinese and English literature separately and the total publication volume, and the bar chart shows the distribution of pain types studied, with different colors indicating different pain types. See the legend in the upper right corner for details. **(B)** Geographical publication distribution map. The intensity of the color corresponds to the number of publications, with darker shades representing higher publication counts.

Figure 2A illustrates publication trends across the eight databases from their inception to October 22, 2024, demonstrating a rising total output in which Chinese publications substantially exceeded English-language publications. Chinese publications maintained a consistent growth, whereas English studies displayed wave-like variations with cyclical patterns from 2012, transitioning to a growth trend after 2019. The first publication on acupuncture for PSP appeared in 1994 focusing on shoulder pain, with the first English study was published in 2012. Publication peaked in 2022 and 2023, with 39 studies in each year. From January to October 2024, 15 studies were published. Geographically, 341 studies (98.6%) were from China, with South Korea contributing 4 studies, and Iran contributing 1 study. Within China, Guangdong and Shandong provinces contributed the most studies (see Figure 2B and Supplementary Appendix 4). Among the 346 publications included in the review, 339 were RCTs and 7 were SRs/MRs. Of the studies, 333 were published in Chinese and 13 were published in English. Fifty-five studies were based on academic dissertations, and articles indexed in the Chinese Science Citation Database and Science Citation Index were in the minority.

**Figure 3:**
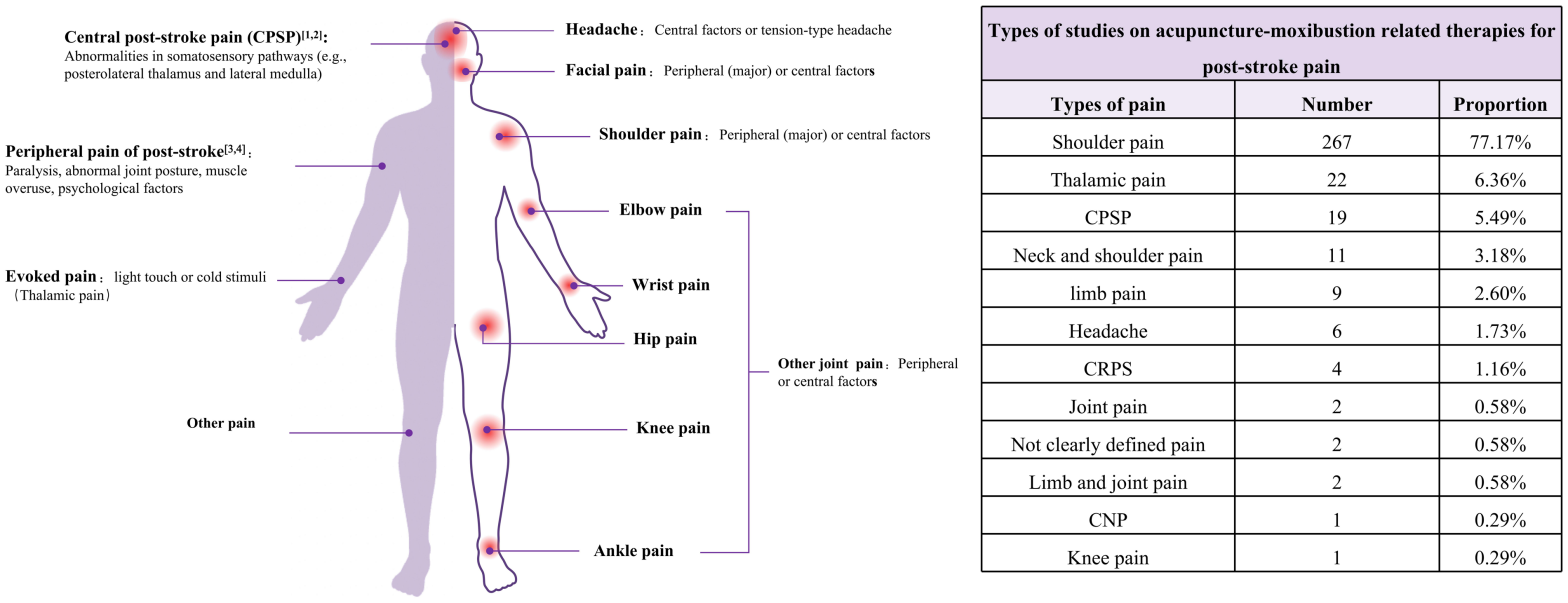
Pain classification and current research on acupuncture for post-stroke pain. The left diagram presents the classification of pain and elucidates the underlying causes of pain. The right-hand table displays the primary pain types covered in the studies referenced in this manuscript.

Given the heterogeneity of pain types and the multifaceted mechanisms underlying PSP (7), we classified pain manifestations in the literature (Figure 3). Shoulder pain (n = 267, 77.2%) emerged as the predominant focus in acupuncture interventions, reflecting the high prevalence of post-stroke shoulder-hand syndrome, with thalamic pain (n = 22, 6.4%) being the second most investigated category.

### 3.3 Basic characteristics of the participants in RCTs

**Figure 4:**
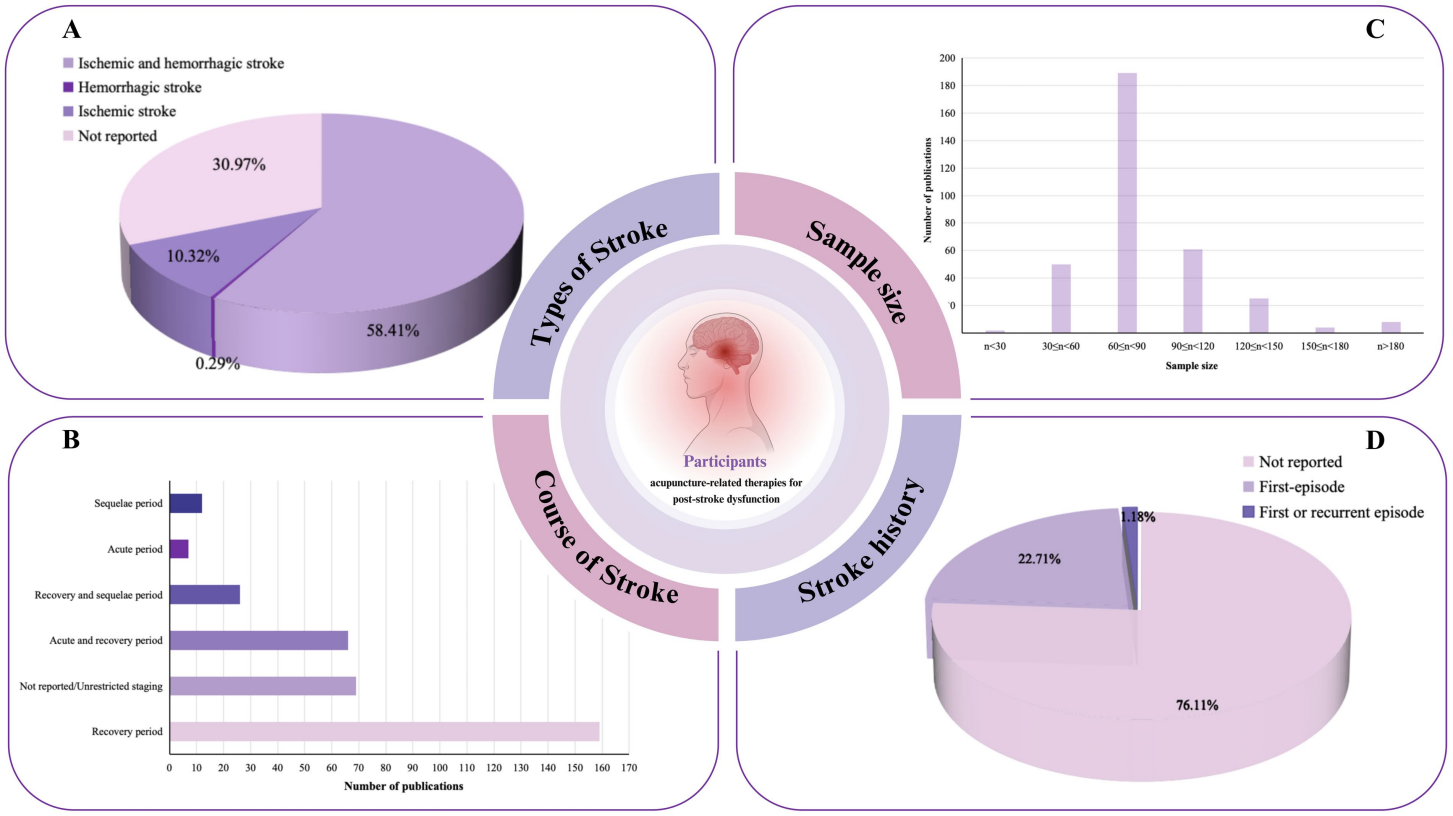
Population characteristics of acupuncture intervention for post-stroke pain. **(A)** Distribution of stroke types included in the research. **(B)** Distribution of stroke stages included in the studies. **(C)** Distribution of sample size of the included RCTs. **(D)** Statistics on the occurrence of first-time strokes.

Of the RCTs, 58.4% enrolled patients with stroke due to either cerebral hemorrhage or cerebral ischemia, whereas 31.0% did not specify the stroke subtype. Only one study selectively included patients with cerebral hemorrhage (Figure 4A), with the majority of studies focusing on patients during the recovery phase (Figure 4B). The sample sizes of the studies generally ranged from 60 to 90 participants, with the maximum sample size reaching 280 and the minimum being 24 (Figure 4C and Supplementary Appendix 3).

The majority of studies included in this evidence map did not specify whether the stroke was a first-time event, with only 22.7% involving patients experiencing their first stroke. In terms of stroke diagnostic criteria, 74 studies used a combination of TCM and conventional medicine diagnostic standards along with imaging diagnosis, 38 studies employed both TCM and conventional medicine criteria, 9 studies used TCM combined with imaging, 87 studies applied conventional medicine alongside imaging diagnosis, 36 cases relied exclusively on imaging, 8 studies used only TCM diagnostics, 39 studies used only conventional medicine diagnostics, and 48 studies did not mention any specific criteria. Refer to Supplementary Appendix 3 for further details.

### 3.4 Basic characteristics of the RCTs interventions

#### 3.4.1 Basic characteristics of the intervention and control groups

**Figure 5:**
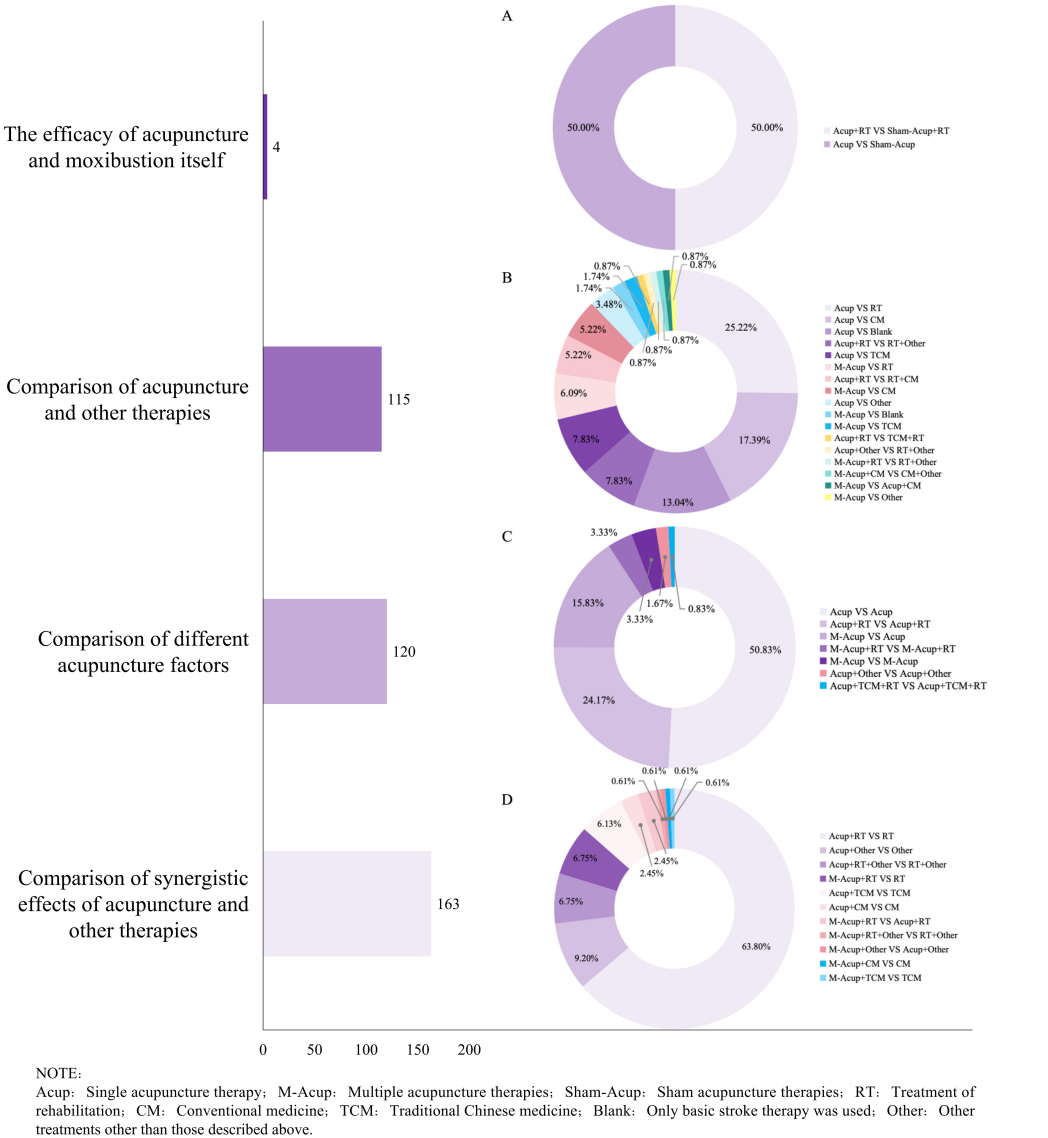
Intervention and control characteristics of acupuncture intervention for post-stroke pain. The left side of the figure shows the purpose of the trial design, and the bars present the total number of designs in each category. The right ring graph presents the specific grouping design for each category. **(A)** Specific grouping design of acupuncture’s own efficacy. **(B)** Specific group design for comparison of acupuncture with other therapies. **(C)** Specific grouping design of different acupuncture factors. **(D)** Specific group design for synergy of acupuncture and other therapies.

This evidence map included a total of 339 randomized controlled studies, comprising not only conventional two-arm studies but also 55 three-arm studies and 2 four-arm studies. To facilitate analysis, multi-arm trials meeting the inclusion criteria were divided, resulting in 402 groups for evaluation. Further details are provided in Supplementary Appendix 5. The 402 control groups were classified according to research aims into: (i) evaluation of acupuncture as a standalone therapy; (ii) comparison of acupuncture efficacy with other therapies; (iii) comparison of different acupuncture factors (acupoints/needling techniques/ therapies); and (iv) evaluation of the synergistic effects of acupuncture with other therapies. The majority of studies aimed to assess the synergistic effects of acupuncture with other therapies (Figure 5). The most common comparison was between acupuncture combined with rehabilitation (RT) versus RT alone (Figure 5D). Among the comparisons of acupuncture with other therapies, the most frequent comparison was acupuncture versus RT (Figure 5B). Additionally, comparisons of different acupuncture therapies and different acupuncture therapies combined with RT were the two most used study designs in exploring various acupuncture factors (Figure 5C). Only four studies evaluated the efficacy of acupuncture alone (Figure 5A). Further details are provided in Supplementary Appendix 6. In the intervention groups, the three most commonly used interventions were acupuncture combined with rehabilitation, acupuncture alone, and a combination of multiple acupuncture methods (Supplementary Appendix 7). The most frequent interventions among the controls were rehabilitation, acupuncture, and acupuncture combined with rehabilitation (Supplementary Appendix 8).

**Figure 6:**
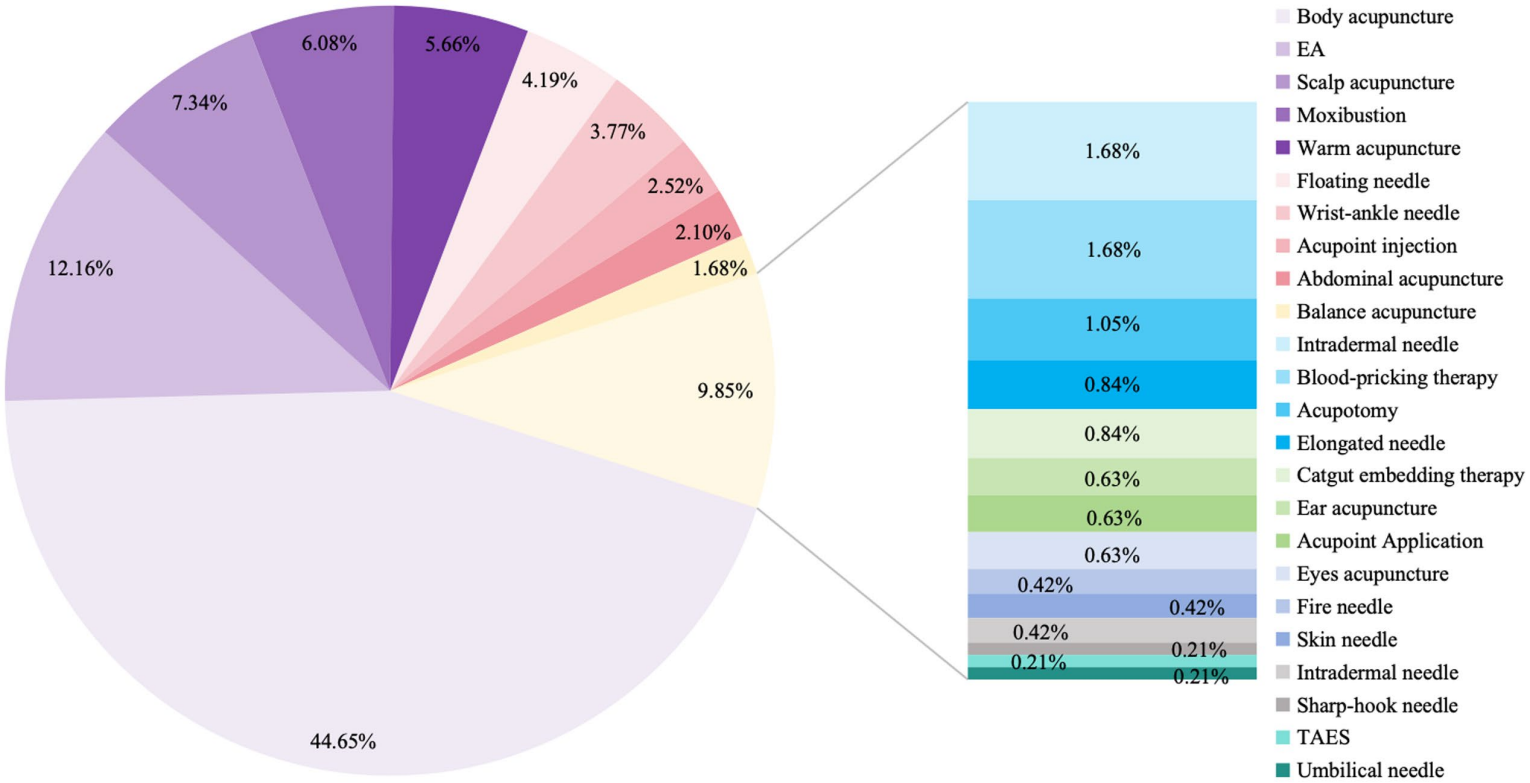
Statistical analysis of the proportion of each acupuncture method in RCTs.

**Figure 7:**
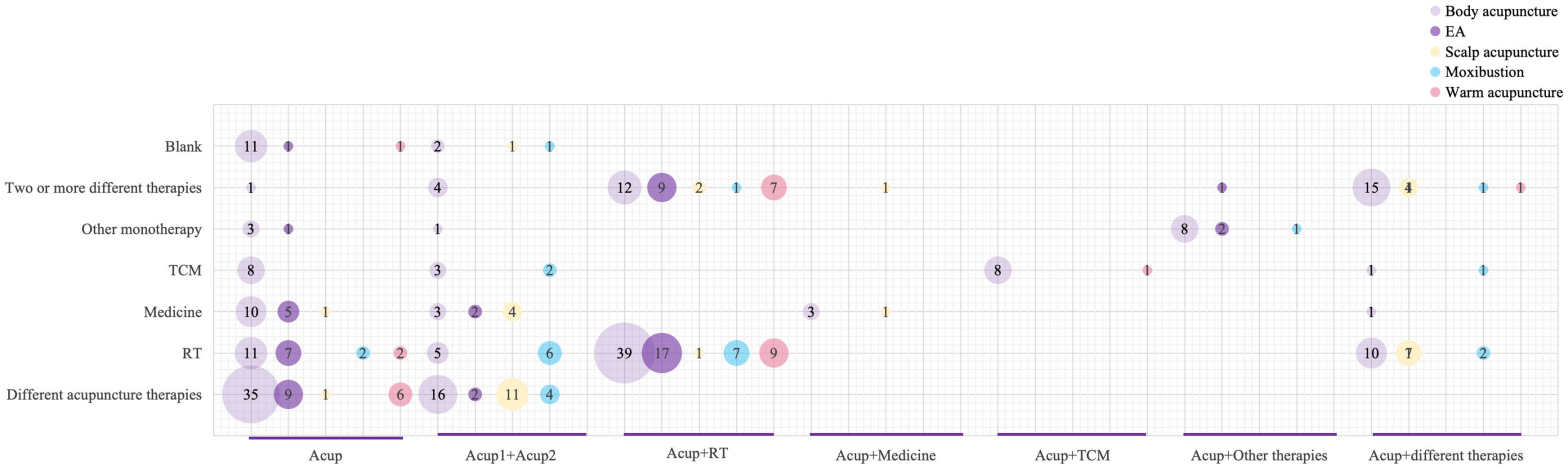
Figure of evidence from clinical studies. The abscissa represents the composition of the observation group, the ordinate represents the control measure, and the bubble size represents the frequency of the study intervention.

We analyzed the subdivided acupuncture-related therapies and found that body acupuncture combined with scalp acupuncture, body acupuncture combined with moxibustion, and scalp acupuncture combined with electroacupuncture were the most favored multi-acupuncture interventions (Supplementary Appendix 9). We analyzed each acupuncture intervention method included in the RCTs, identifying body acupuncture, electroacupuncture, scalp acupuncture, moxibustion, and warm needling as the five most commonly used interventions (Figure 6 and Supplementary Appendix 10). Notably, floating needle and wrist-ankle acupuncture were frequently used as acupuncture interventions for PSP. The bubble chart in Figure 7 shows the use of these five interventions in the studies. Body acupuncture and electroacupuncture were the most frequently used acupuncture interventions in comparisons of acupuncture plus RT versus RT, and in comparisons of different acupuncture therapies. Moxibustion was primarily used in comparisons of multi-acupuncture versus single-acupuncture approaches.

The number of intervention sessions differed among the various acupuncture therapies. The treatment duration generally ranged from 2 to 4 weeks, with few studies extending beyond 6 weeks (Supplementary Appendices 5, 11).

#### 3.4.2 Acupuncture intervention points

Data on acupoints, meridians, body regions, and specific points were extracted from 346 RCTs, yielding 484 acupuncture prescriptions that involved 284 acupoints and trigger points/tender points/myofascial trigger points.

**Figure 8:**
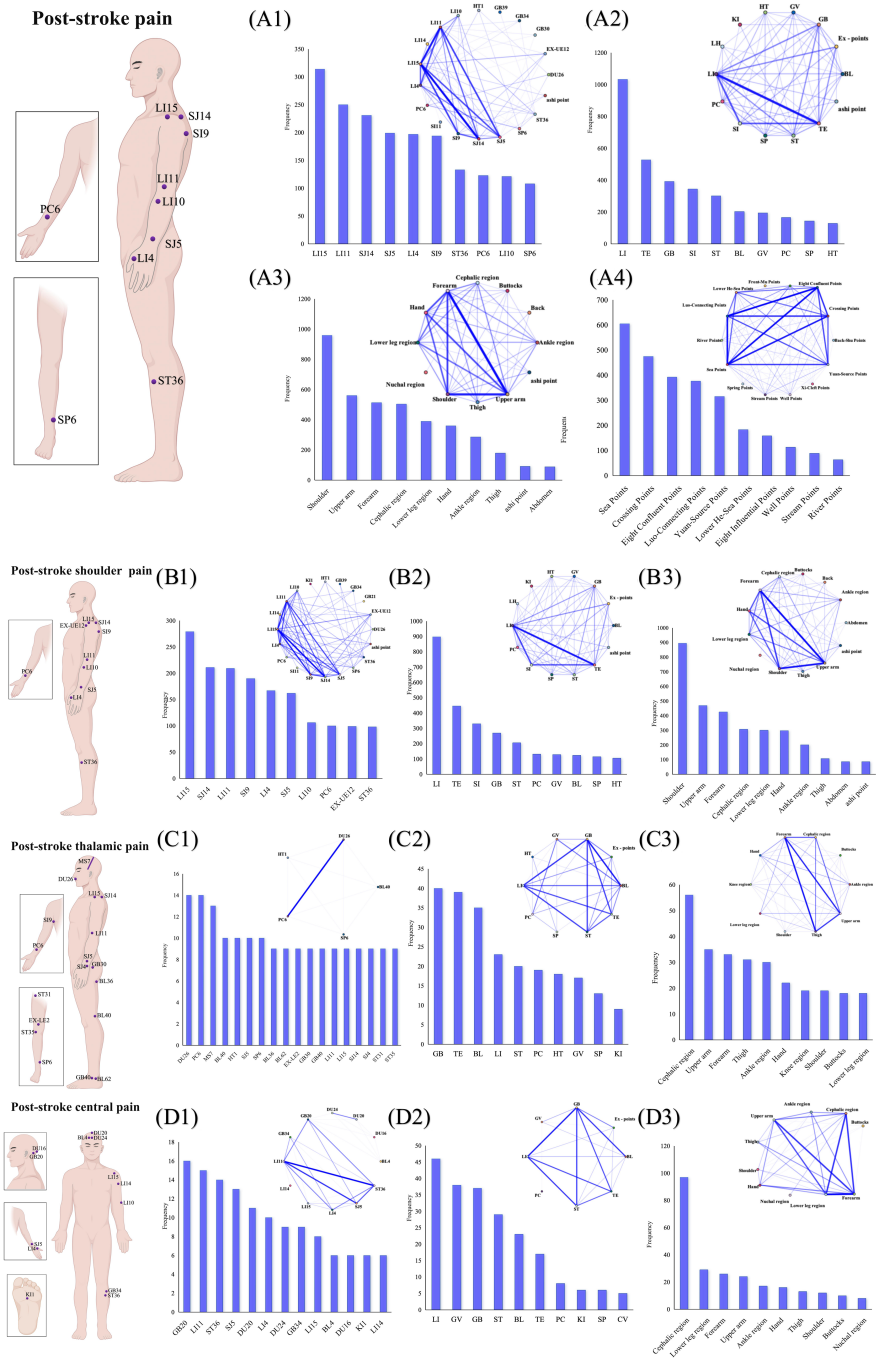
Overview of acupuncture intervention points. On the left side of the image, from top to bottom, are the top 10 acupoint charts for post-stroke pain, post-stroke shoulder pain, thalamic pain, and central pain. On the right side of the image, the lower left shows frequency displays, and the upper right shows association network diagrams. **(A1)** Frequency and association network of acupoints for post-stroke pain. **(A2)** Frequency and association network of meridians for post-stroke pain. **(A3)** Frequency and association network of body regions for post-stroke pain. **(A4)** Frequency and association network of specific points for post-stroke pain. **(B1)** Frequency and association network of acupoints for post-stroke shoulder pain. **(B2)** Frequency and association network of meridians for post-stroke shoulder pain. **(B3)** Frequency and association network of body regions for post-stroke shoulder pain. **(C1)** Frequency and association network of acupoints for post-stroke thalamic pain. **(C2)** Frequency and association network of meridians for post-stroke thalamic pain. **(C3)** Frequency and association network of body regions for post-stroke thalamic pain. **(D1)** Frequency and association network of acupoints for post-stroke central pain. **(D2)** Frequency and association network of meridians for post-stroke central pain. **(D3)** Frequency and association network of body regions for post-stroke central pain.

The most frequently used acupoints were Jianyu (LI15), Jianliao (SJ14), Quchi (LI11), Jianzhen (SI9), Hegu (LI4), Waiguan (SJ5), Shousanli (LI10), Jianqian (EX-UE12), Zusanli (ST36), and Neiguan (PC6). Furthermore, Ashi points, tender points, and myofascial trigger points were also frequently used in PSP treatment. The top 10 meridians were the Stomach Meridian of Foot-Yangming (LI), Triple Energizer Meridian of Hand-Shaoyang (TE), Large Intestine Meridian of Hand-Yangming (SI), Gallbladder Meridian of Foot-Shaoyang (GB), Small Intestine Meridian of Hand-Taiyang (ST), Extra Meridian Points (Ex-points), Pericardium Meridian of Hand-Jueyin (PC), Governor Vessel (GV), Bladder Meridian of Foot-Taiyang (BL), and Spleen Meridian of Foot-Taiyin (SP). Body regions such as the shoulder, upper arm, forearm, lower leg region, and cephalic region were also frequently used. High-frequency specific points with unique therapeutic effects included Sea Points, Crossing Points, Eight Confluent Points, Luo-Connecting Points, and Yuan-Source Points (Supplementary Appendix 12). The most common acupoint links were LI15–SJ14 (214), LI11–LI15 (204), and LI15–SI9 (176) (Figure 8A1). Similarly, the three most common meridian links were LI–TE (315), LI–SI (228), and SI–TE (217) (Figure 8A2). The Forearm–Upper arm (292) and Eight Confluent Points–Luo-Connecting Points (246) were the most strongly associated body regions and specific points, respectively (Figures 8A3,4 and Supplementary Appendix 13).

Our analysis focused on the three most-studied pain types (Supplementary Appendices 12, 13). For post-stroke shoulder pain, Jianyu (LI15), Jianliao (SJ14), Quchi (LI11), Jianzhen (SI9), and Hegu (LI4) were the most frequently used acupoints (Figure 8B1). The most frequently targeted meridians were LI, TE, and SI. As expected, the shoulder, upper arm, and forearm were the predominant treatment regions. The top three acupoint pairs were LI15–SJ14 (195), LI11–LI15 (173), and LI15–SI9 (172). The most commonly paired meridians and regions were LI–TE (276) and Forearm-Upper arm (242) (Figures 8B2,3). In studies of acupuncture for thalamic pain, the five most frequently used acupoints were Shuigou (DU26), Neiguan (PC6), posterior oblique line of vertex-temporal (MS7), Weizhong (BL40), and Jiquan (HT1). The predominant meridians targeted were GB, TE, and GB. The cephalic region, upper arm, and forearm were the most frequently treated areas (Figures 8C1–3). In the treatment of central pain, the most commonly targeted acupoints were Fengchi (GB20), Quchi (LI11), and Zusanli (ST36). The most frequently used meridians were LI, GV, and GB, with the cephalic region, lower leg region, and forearm being the primary treatment areas (Figures 8D1–3).

**Figure 9:**
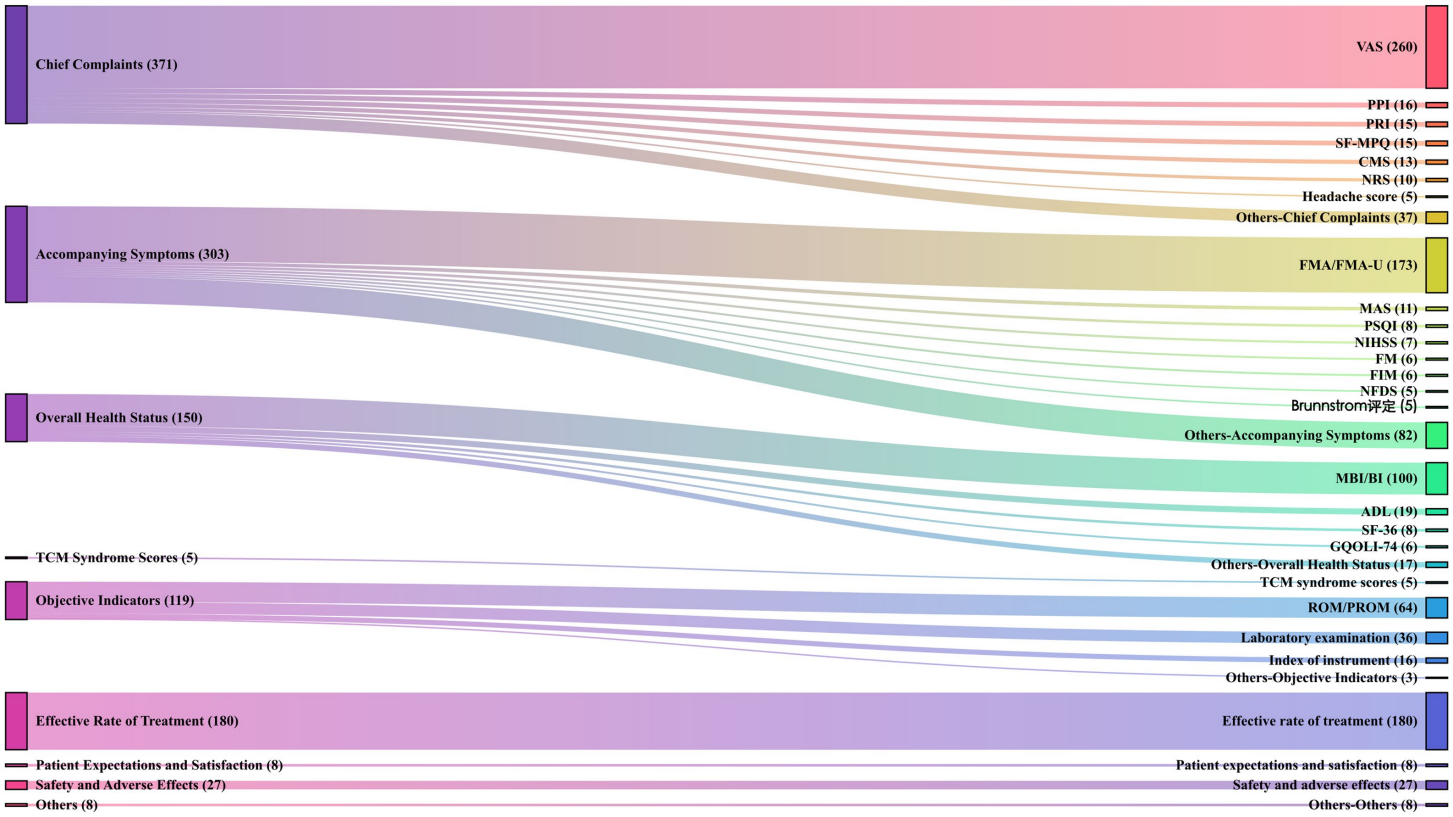
Outcomes characteristics of acupuncture intervention for post-stroke pain. The primary classification of the outcome measures is shown on the left, and the specific outcome measures with their frequencies in parentheses are shown on the right.

We categorized all outcome measures into the following groups: (i) improvement in main complaint-related symptoms, (ii) improvement in accompanying symptoms, (iii) overall health assessment, (iv) changes in TCM syndrome patterns, (v) objective indicators, (vi) effective rate, (vii) safety evaluation, (viii) satisfaction assessment, and (ix) others. Details are provided in Supplementary Appendix 14. The acupuncture interventions focused primarily on pain relief, with the visual analog scale (VAS) being the most frequently used measure outcome measure, as shown in the Sankey diagram in Figure 9. More than half of the studies evaluated the efficacy of acupuncture interventions based on VAS improvements. Furthermore, significant attention was given to daily functional abilities and the influence of pain alleviation on patients’ quality of life. For objective indicators, the primary focus was on joint range of motion and laboratory tests. Safety evaluations of acupuncture interventions were conducted in only 27 studies.

### 3.5 Methodological quality assessment of RCTs

Among the 339 included RCTs, 218 were classified as low risk of bias due to the use of appropriate randomization methods, including 159 studies that used random number tables and some that used computer software for randomization. Additionally, 18 studies were rated as high risk of bias because they used the order of consultation or admission for randomization. In terms of allocation concealment, only 18 articles were considered low risk of bias, as they mostly used envelope methods or computers for allocation concealment, and the remaining studies did not describe the allocation concealment method. The unique characteristics of acupuncture interventions often resulted in high risk of bias in blinding assessments, and the subjective nature of pain meant that patient and outcome blinding were generally classified as high risk of bias. In terms of data completeness, 130 studies had missing data but did not provide detailed analysis, and only 1 study, which was classified as low risk of bias had a protocol and reported relevant outcomes (Supplementary Appendices 3, 15).

### 3.6 Synthesis of the evidence

**Figure 10:**
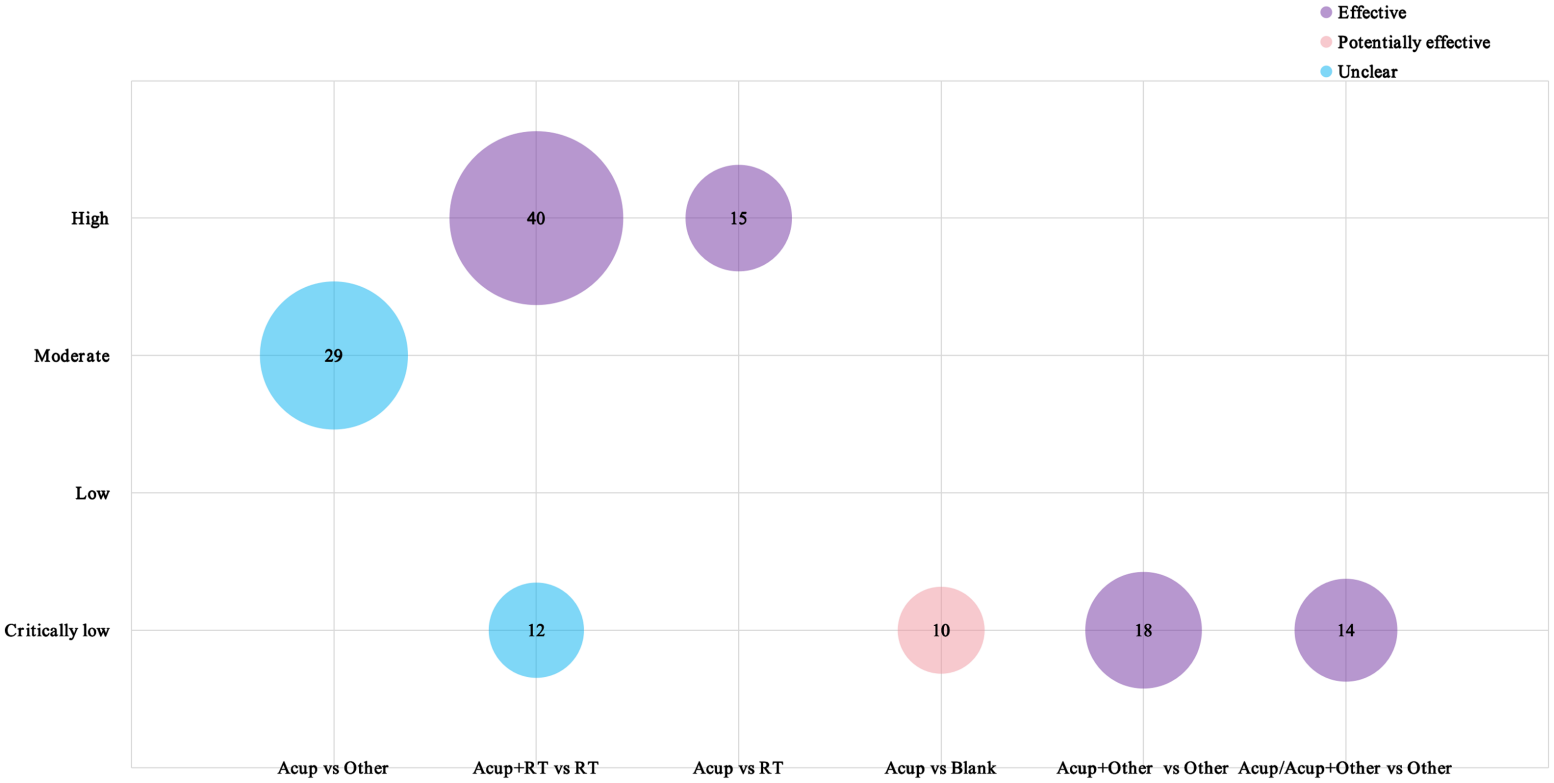
Evidence map of systematic reviews. The X-axis denotes the types of RCT designs included in the SRs/Mas; the Y-axis indicates the study quality; the varying colors indicate different research conclusions; and the numbers inside the circles indicate the number of RCTs included in the studies.

We conducted a quality assessment of the 7 SRs/MAs that were included, and among them, 4 studies were rated as very-low quality, and only 2 studies were rated as high quality (Supplementary Appendix 16). We also classified their findings into three categories: acupuncture being superior to the control group, acupuncture interventions having potential advantages, and inconclusive results. As shown in the bubble chart of Figure 10, a high-quality SRs/MA study that included 40 RCTs demonstrated that the synergistic effect of acupuncture combined with rehabilitation was significantly superior to RT alone, and the efficacy of acupuncture alone was also better than that of RT alone. The four very low-quality studies compared acupuncture plus RT to RT alone, acupuncture to conventional treatment, and acupuncture combined with other therapies to other therapies. Two studies showed that acupuncture was effective or potentially effective, whereas the other 2 studies concluded that the efficacy of acupuncture is unclear, although these studies were limited by small sample sizes and low methodological quality.

## 4 Discussion

Acupuncture is regarded as a valuable approach for stroke recovery, and its analgesic effects have consistently been a focal point and hotspot in research. Due to the abuse and addictive potential of painkillers, physical therapy or complementary and alternative therapies are recommended in clinical practice guidelines for pain management (25, 26). Our study provides an overview of research on acupuncture interventions for PSP. The results suggest a growing interest in acupuncture for PSP, reaching a peak in recent years. However, English publications started later and remain relatively limited, with most studies were conducted in China, particularly in Guangdong and Shandong Provinces. This may be related to the incidence of stroke, economic development, and the level of emphasis on scientific research in these provinces. The overall quality of journals publishing these studies was limited, indicating that the international influence and research quality of acupuncture-related therapies for PSP requires improvement.

One study showed that 87% of strokes are ischemic, 10% are intracerebral hemorrhages (ICHs), and 3% are subarachnoid hemorrhages (SAHs) (2). In our study patients with ischemic stroke were the most frequently studied, particularly those in the recovery phase. As a retrospective cohort study found that the first 3 months after stroke discharge are critical for rehabilitation and quality of life improvement for most patients. For patients with severe conditions, financial constraints, or limited social support, extended rehabilitation and support from 3 to 6 months post-discharge are essential to optimize their quality of life (27). In this study, post-stroke shoulder pain was the most frequently studied condition in acupuncture interventions for stroke, consistent with prior research (28, 29), Shoulder pain is strongly linked to post-stroke shoulder subluxation, rotator cuff injuries, and neurological impairments (30), with an incidence rate of 25–72% after stroke (31), Our findings highlight that shoulder pain continues to be a major focus in acupuncture research for PSP, reflecting its importance and complexity in rehabilitation. Most RCTs included in the analysis did not report the stroke types among participants, and the diagnostic criteria were inconsistent. Additionally, the pain levels of the participants were often unreported. The causes of PSP are diverse, and the location and characteristics of the pain are closely tied to the stroke site. For instance, a previous study found that the ventral posterior nucleus-thalamic junction is strongly associated with thalamic pain (32). Thus, future research should rigorously apply inclusion and exclusion criteria, use internationally recognized diagnostic standards for stroke, and incorporate information on stroke locations. This approach will improve study quality and facilitate more informed acupuncture treatment strategies, such as the choice of acupuncture techniques (33).

Ischemic stroke and pain syndromes have become hot topics in acupuncture research (34), with post-stroke rehabilitation playing a vital role in enhancing patients’ quality of life. Our evidence map indicates that the synergistic effects of acupuncture combined with rehabilitation and the comparative efficacy of various acupuncture therapies are key clinical issues in acupuncture today. The combination of body and scalp acupuncture was the most commonly used clinical intervention. Notably, many new acupuncture therapies have emerged in recent years, including floating needle and transcutaneous electrical acupoint stimulation. A network analysis revealed that the top three interventions for VAS improvement, compared with single rehabilitation training, were floating needle, rehabilitation plus catgut embedding, and other multi-acupuncture combinations (35). The evidence map revealed that the application of micro-acupuncture systems was widely used for PSP, with wrist-ankle needle, abdominal needle, and eyes needle being widely used. The selection of acupoints varied for different types of pain. For shoulder pain, traditional shoulder three-needle techniques were primarily used, whereas for central pain, the GV and scalp acupuncture points were emphasized. Ashi points, due to their unique characteristics, were widely used in PSP. In addition to traditional acupoints, trigger points and myofascial nodes based on modern anatomical medicine were also used. This indicates that traditional acupuncture is continuously evolving. As a study suggests, comparative research on traditional and modern acupuncture techniques is a promising direction for future in-depth studies. This would not only reveal their relative therapeutic effects but might also reveal new intervention strategies and mechanisms to identify the best methods for acupuncture intervention in PSP (36).

The setting of outcome measures is aimed at better evaluating the clinical efficacy of interventions, with their appropriateness, objectivity, and depth impacting clinical decisions. Our study revealed that acupuncture analgesia primarily emphasized pain relief, with considerable attention also given to the effects of pain on mobility and quality of life. Because pain is a subjective experience, objective indicators are difficult to measure, leading to more research on mechanisms such as inflammation and assessments of joint mobility. Patients with stroke are a unique population, with pain impacting both their daily functioning and their ability to reintegrate into society, an area that has received limited attention in our research. In addition to clinical efficacy observations, future research could incorporate imaging studies of acupuncture, filling this gap in the future.

The overall methodological quality of studies, including RCTs and MAs/SRs, was suboptimal, with a notable scarcity of research focusing on PSP. Our findings indicate that few clinical studies conducted sample size estimations, and some studies did not report specific randomization methods or address aspects such as allocation concealment and blinding procedures. The nature of acupuncture interventions complicates practitioner blinding, and although sham acupuncture can theoretically blind patients, ethical considerations in stroke research often preclude its use, creating substantial barriers to effective blinding. Pain is a highly subjective experience. Blinding the outcome assessors for subjective measures can effectively mitigate potential measurement bias (37). Moreover, rigorous standardization of acupoint selection, needling sites, insertion depth, and treatment frequency is essential. Addressing these factors in future research would strengthen the evidence base.

## 5 Strengths and limitations

To our knowledge, this study is the first to use an evidence map to illustrate the current state of research on acupuncture for PSP. The findings of this study suggest that acupuncture offers some advantages in the treatment of PSP.

However, this study has some limitations. First, this study only included Chinese and English databases and did not search the gray literature, which introduced some limitations in the evidence sources. Future studies should incorporate a wider range of databases and ensure regular updates to the research data. Additionally, acupuncture, being a highly distinctive Chinese medical practice, is widely used and highly accepted in China, which led to the majority of included studies being conducted in China. During the classification of intervention types, we divided the data, which may have influenced the overall findings. The inclusion of a wide range of acupuncture interventions, with variations in drug types, dosages, and treatment durations for each intervention/control measure, this imposed some limitations on the applicability of the study results.

## 6 Conclusion

Despite the long history of acupuncture analgesia, the effectiveness of acupuncture differs for various types of pain because of the complex mechanisms underlying PSP. It is evident that the efficacy of acupuncture for post-stroke shoulder pain has received significant attention, but acupuncture intervention for central pain still requires further research. This study suggests that leveraging the significant therapeutic advantages of acupuncture to prevent/treat PSP can both alleviate symptoms and improve quality of life, but more high-quality studies are needed to provide higher-level strength of evidence.

## Data Availability

The original contributions presented in the study are included in the article/Supplementary material, further inquiries can be directed to the corresponding author.

## Footnotes

^1^ https://www.chiplot.online/

^2^ www.figdraw.com

## References

[1] Feigin VL Brainin M Norrving B Martins S Sacco RL Hacke W . World stroke organization (WSO): global stroke fact sheet 2022. Int J Stroke. (2022) 17:18–29. doi: 10.1177/17474930211065917, PMID: 34986727

[2] Martin SS Aday AW Almarzooq ZI Anderson CAM Arora P Avery CL . 2024 heart disease and stroke statistics: a report of US and global data from the American Heart Association. Circulation. (2024) 149:e347–913. doi: 10.1161/CIR.0000000000001209, PMID: 38264914 PMC12146881

[3] Woolf CJ. Central sensitization: implications for the diagnosis and treatment of pain. Pain. (2011) 152:S2–S15. doi: 10.1016/j.pain.2010.09.030, PMID: 20961685 PMC3268359

[4] Scholz J Woolf CJ. The neuropathic pain triad: neurons, immune cells and glia. Nat Neurosci. (2007) 10:1361–8. doi: 10.1038/nn1992 17965656

[5] Sandkühler J. Models and mechanisms of hyperalgesia and allodynia. Physiol Rev. (2009) 89:707–58. doi: 10.1152/physrev.00025.2008, PMID: 19342617

[6] Ma Y Luo J Wang XQ. The effect and mechanism of exercise for post-stroke pain. Front Mol Neurosci. (2022) 15:1074205. doi: 10.3389/fnmol.2022.1074205, PMID: 36533131 PMC9755671

[7] Hansen AP Marcussen NS Klit H Andersen G Finnerup NB Jensen TS. Pain following stroke: a prospective study. Eur J Pain. (2012) 16:1128–36. doi: 10.1002/j.1532-2149.2012.00123.x, PMID: 22407963

[8] Bovim MR Indredavik B Hokstad A Lydersen S Askim T. New-onset pain in the early phase and three months following stroke – data from a multicenter study. J Pain Res. (2018) 11:1869–76. doi: 10.2147/JPR.S165482, PMID: 30271192 PMC6147539

[9] Haslam BS Butler DS Kim AS Carey LM. Somatosensory impairment and chronic pain following stroke: an observational study. Int J Environ Res Public Health. (2023) 20:906. doi: 10.3390/ijerph20020906, PMID: 36673661 PMC9859194

[10] Tamasauskas A Silva-Passadouro B Fallon N Frank B Laurinaviciute S Keller S . Management of central poststroke pain: systematic review and meta-analysis. J Pain. (2025) 26:104666. doi: 10.1016/j.jpain.2024.104666, PMID: 39260808

[11] Klit H Finnerup NB Jensen TS. Central post-stroke pain: clinical characteristics, pathophysiology, and management. Lancet Neurol. (2009) 8:857–68. doi: 10.1016/S1474-4422(09)70176-0, PMID: 19679277

[12] Asadauskas A Stieger A Luedi MM Andereggen L. Advancements in modern treatment approaches for central post-stroke pain: a narrative review. J Clin Med. (2024) 13:5377. doi: 10.3390/jcm13185377, PMID: 39336863 PMC11432561

[13] Deng L He Y Li J. Clinical research progress of acupuncture and moxibustion in the treatment of central pain after stroke. J Extern Ther Tradit Chin Med. (2021) 30:90–2.

[14] Birch S Robinson N. Acupuncture as a post-stroke treatment option: a narrative review of clinical guideline recommendations. Phytomedicine. (2022) 104:154297. doi: 10.1016/j.phymed.2022.154297, PMID: 35816994

[15] Saragih ID Suarilah I Mulyadi M Saragih IS Lee BO. Beneficial effects of non-pharmacological interventions for post-stroke pain: a meta-analysis. J Nurs Scholarsh. (2025) 57:239–52. doi: 10.1111/jnu.13032 39513537

[16] Li Y Li X Li R Cao L Li M Li H . Production and reporting of evidence maps. Chin J Evid Based Med. (2020) 20:1098–103.

[17] Snilstveit B Vojtkova M Bhavsar A Stevenson J Gaarder M. Evidence & gap maps: a tool for promoting evidence informed policy and strategic research agendas. J Clin Epidemiol. (2016) 79:120–9. doi: 10.1016/j.jclinepi.2016.05.015, PMID: 27387966

[18] Tian J Li L Zhang J. Notes for writing evidence charts. Drug Eval China. (2019) 36:81–5.

[19] Yu X Xu G Tian H Chen M Li Q Sun M . Evidence map analysis of clinical studies on acupuncture and moxibustion in the treatment of chronic obstructive pulmonary disease. J Chengdu Univ Tradit Chin Med. (2024) 47:65–73.

[20] Li Q Ge Z Huang S Zhang L Li B. Evidence map analysis of clinical research on the treatment of chronic kidney disease with traditional Chinese medicine in the past ten years. Chin J Tradit Chin Med. (2024) 49:5610–26. 10.19540/j.cnki.cjcmm.20240627.501 39701743

[21] Li X Huang X Zhang P Zheng X Kong D Wu J. Evidence map analysis of clinical research of traditional Chinese medicine injection in the treatment of chronic obstructive pulmonary disease in the past 6 years. China TCM Emerg. (2024) 33:565–70.

[22] Higgins JPT Altman DG Gøtzsche PC Jüni P Moher D Oxman AD . The Cochrane collaboration’s tool for assessing risk of bias in randomised trials. BMJ. (2011) 343:d5928. doi: 10.1136/bmj.d5928, PMID: 22008217 PMC3196245

[23] Shea BJ Reeves BC Wells G Thuku M Hamel C Moran J . AMSTAR 2: a critical appraisal tool for systematic reviews that include randomised or non-randomised studies of healthcare interventions, or both. BMJ. (2017) 358:j4008. doi: 10.1136/bmj.j4008, PMID: 28935701 PMC5833365

[24] Page MJ McKenzie JE Bossuyt PM Boutron I Hoffmann TC Mulrow CD . The PRISMA 2020 statement: an updated guideline for reporting systematic reviews. BMJ. (2021) 372:n71. doi: 10.1136/bmj.n71, PMID: 33782057 PMC8005924

[25] Wu Z Tan W Li S Zhang W Lai M Luo W. Research hotspots and trends of acupoint and pain based on PubMed: a bibliometric analysis. Front Neurol. (2024) 15:1498576. doi: 10.3389/fneur.2024.1498576, PMID: 39895908 PMC11783567

[26] Nahin RL Rhee A Stussman B. Use of complementary health approaches overall and for pain management by US adults. JAMA. (2024) 331:613–5. doi: 10.1001/jama.2023.26775, PMID: 38270938 PMC10811586

[27] Butsing N Voss JG Keandoungchun J Thongniran N Griffin MTQ. Changes of health-related quality of life within 6 months after stroke by clinical and sociodemographic factors. Sci Rep. (2025) 15:416. doi: 10.1038/s41598-024-84454-5, PMID: 39747957 PMC11695920

[28] Neto IS Guimaraes M Ribeiro T Gonçalves A Natario I Torres M. Retrospective cohort study on the incidence and management of hemiplegic shoulder pain in stroke inpatients. Cureus. (2024) 16:e76030. doi: 10.7759/cureus.76030, PMID: 39835068 PMC11743632

[29] Broussy S Saillour-Glenisson F García-Lorenzo B Rouanet F Lesaine E Maugeais M . Sequelae and quality of life in patients living at home 1 year after a stroke managed in stroke units. Front Neurol. (2019) 10:907. doi: 10.3389/fneur.2019.00907, PMID: 31496987 PMC6712081

[30] Anwer S Alghadir A. Incidence, prevalence, and risk factors of hemiplegic shoulder pain: a systematic review. Int J Environ Res Public Health. (2020) 17:4962. doi: 10.3390/ijerph17144962, PMID: 32660109 PMC7400080

[31] Hoang Le LT Huong Nguyen DT Tang HK Luu VT Pham NA Le LB. Investigating the traditional medicine shoulder pain (Jian Tong) characteristics in patients with ischaemic stroke in the early rehabilitation phase. Heliyon. (2024) 10:e24626. doi: 10.1016/j.heliyon.2024.e24626, PMID: 38298670 PMC10828057

[32] Sprenger T Seifert CL Valet M Andreou AP Foerschler A Zimmer C . Assessing the risk of central post-stroke pain of thalamic origin by lesion mapping. Brain. (2012) 135:2536–45. doi: 10.1093/brain/aws153, PMID: 22719000

[33] Wise S Lorenc A. Anatomical and clinical characteristics of scalp acupuncture systems: a scoping review and synthesis. J Acupunct Meridian Stud. (2023) 16:159–75. doi: 10.51507/j.jams.2023.16.5.159, PMID: 37885251

[34] Zhang B Shi H Cao S Xie L Ren P Wang J . Revealing the magic of acupuncture based on biological mechanisms: a literature review. Biosci Trends. (2022) 16:73–90. doi: 10.5582/bst.2022.01039, PMID: 35153276

[35] Huang T Yao H Huang J Wang N Zhou C Huang X . Effectiveness of acupuncture for pain relief in shoulder-hand syndrome after stroke: a systematic evaluation and Bayesian network meta-analysis. Front Neurol. (2023) 14:1268626. doi: 10.3389/fneur.2023.1268626, PMID: 38046583 PMC10693460

[36] Wang Q Zhang Q Lu F Hu H Zhu M. Trends in acupuncture therapy for microcirculation and hemorheology from 1998 to 2023: a bibliometric and visualized study. J Pain Res. (2024) 17:177–96. doi: 10.2147/JPR.S441512, PMID: 38223661 PMC10785693

[37] Wu T Liu D Huang J Mai Y Zhao X Sun W . Application of risk of bias assessment tool in Cochrane systematic reviews of acupuncture. Chin J Evid Based Med. (2014) 14:361–4.

