# Supplementary material for: Acupuncture-related therapies for post-stroke pain management: a scoping review and evidence map
Source: Front Neurol. 2025 Aug 6;16:1604655. doi: 10.3389/fneur.2025.1604655 (PMC12365601; doi:10.3389/fneur.2025.1604655)
Supplement: Supplementary file 1 [file Data_Sheet_1.pdf]

Supplementary Material

Appendix 1. Search strategy in selective databases.

Appendix 2.Data Standardization

Appendix 3.Detailed table of population characteristics.

Appendix 4.The regional distribution of literature was included.

Appendix 5.Detailed table of Intervention and control design characteristics of RCTs.

Appendix 6.The intervention and control design of the included RCTs.

Appendix 7.Frequency statistics of intervention categories.

Appendix 8.Frequency statistics of control categories.

Appendix 9.Statistics of acupuncture interventions included in RCTs.

Appendix 10.Statistical analysis of the frequency of each acupuncture method in RCTs.

Appendix 11. Statistical analysis of the frequency of each acupuncture method in RCTs.

Appendix 12. Statistical analysis of the frequency of acupoint related information in RCTs.

Appendix 13.The high-frequency links of selected acupoint,Meridian, body parts and specific points in 484 acupuncture prescriptions.

Appendix 14.Details of the outcome measures of the included RCTs.

Appendix 15.Risk assessment of RCTs.

Appendix 16.Basic information and quality assessment of SRs/MAs.

Appendix 1. Search strategy in selective databases.

Time： The database was established to 2024.10.20

We searched eight language databases databases: MEDLINE (Pubmed), Embase, Cochrane Library, WOS， CNKI, Wanfang Data, Chongqing VIP, Sinomed to retrieve relevant studies.

Database	Search Term	Results
PubMed	#1 Acupuncture OR Acupuncture Therapy OR Moxibustion[MeSH Terms]<br>#2 Acupuncture[Title/Abstract] OR Acupuncture Therapy[Title/Abstract] OR Acupuncture Treatment[Title/Abstract] OR Treatment, Acupuncture[Title/Abstract] OR Therapy, Acupuncture[Title/Abstract] OR Manual Acupuncture[Title/Abstract] OR Acupuncture-moxibustion[Title/Abstract] OR Moxibustion[Title/Abstract] OR Moxabustion[Title/Abstract] OR Warm needle[Title/Abstract] OR Warm Acupuncture[Title/Abstract] OR Thermoacupuncture[Title/Abstract] OR Electroacupuncture[Title/Abstract] OR Electro-acupuncture[Title/Abstract] OR electric acupuncture[Title/Abstract] OR Acupuncture Point[Title/Abstract] OR Point, Acupuncture[Title/Abstract] OR Acupoint[Title/Abstract] OR Pharmacopuncture[Title/Abstract] OR Pharmacoacupuncture Treatment[Title/Abstract] OR Treatment, Pharmacoacupuncture[Title/Abstract] OR Pharmacoacupuncture Therapy[Title/Abstract] OR Therapy, Pharmacoacupuncture[Title/Abstract] OR Acupoint injection[Title/Abstract] OR acupuncture injection[Title/Abstract] OR pharmaco-acupuncture[Title/Abstract] OR auricular needle[Title/Abstract] OR Acupunctures, Ear[Title/Abstract] OR Ear Acupuncture[Title/Abstract] OR Ear needle[Title/Abstract] OR earlobe acupuncture[Title/Abstract] OR Auricular Acupuncture[Title/Abstract] OR Auriculotherapy[Title/Abstract] OR Acupuncture, Auricular[Title/Abstract] OR auricular plaster therapy[Title/Abstract] OR auricular point sticking[Title/Abstract] OR Auricular pressure[Title/Abstract] OR Fire needle[Title/Abstract] OR Fire acupuncture[Title/Abstract] OR Acupoint catgut embedding[Title/Abstract] OR Scalp acupuncture[Title/Abstract] OR Scalp needle[Title/Abstract] OR Scalp electroacupuncture[Title/Abstract] OR scalp stimulation[Title/Abstract] OR Eye needle[Title/Abstract] OR Eye acupuncture[Title/Abstract] OR Abdominal acupuncture[Title/Abstract] OR Abdominal needle[Title/Abstract] OR filiform needle[Title/Abstract] OR silver needle[Title/Abstract] OR three-edged needle[Title/Abstract] OR intradermal needle[Title/Abstract] OR Point application[Title/Abstract] OR needle-embedding[Title/Abstract] OR Catgut Embedding[Title/Abstract] OR pricking therapy[Title/Abstract] OR point injection[Title/Abstract] OR Skin Acupuncture[Title/Abstract] OR transcutanclus electrical acupoint stimulation[Title/Abstract] OR TEAS[Title/Abstract] OR electrical acupoint stimulation[Title/Abstract] OR Acupuncture Point[Title/Abstract] OR Acupoint[Title/Abstract]<br>#3 #1 OR #2<br>#4 Stroke OR Apoplexy[MeSH Terms]<br>#5 "stroke*"[Title/Abstract] OR "Apoplexy"[Title/Abstract] OR "cerebrovascular apoplexy"[Title/Abstract] OR "apoplexy cerebrovascular"[Title/Abstract] OR "cerebrovascular stroke*"[Title/Abstract] OR "cerebral stroke*"[Title/Abstract] OR "acute stroke*"[Title/Abstract] OR "embolic stroke*"[Title/Abstract] OR "wake up stroke*"[Title/Abstract] OR "ischemic stroke*"[Title/Abstract] OR "thrombotic stroke*"[Title/Abstract] OR "brain ischemia"[Title/Abstract] OR "stroke* cerebrovascular"[Title/Abstract] OR "stroke* cerebral"[Title/Abstract] OR "stroke* acute"[Title/Abstract] OR "brain infarction"[Title/Abstract] OR "cerebral infarct*"[Title/Abstract] OR "intracerebral hemorrhage*"[Title/Abstract] OR "cerebral hemorrhage*"[Title/Abstract] OR "cerebral brain hemorrhage*"[Title/Abstract] OR "hemorrhagic stroke*"[Title/Abstract] OR "hypertensive intracerebral hemorrhage*"[Title/Abstract] OR ("hemorrhage*"[All Fields] AND "Cerebrum"[Title/Abstract]) OR (("Cerebrum"[MeSH Terms] OR "Cerebrum"[All Fields] OR "cerebrums"[All Fields]) AND "hemorrhage*"[Title/Abstract]) OR "cerebral parenchymal hemorrhage*"[Title/Abstract] OR ("hemorrhage*"[All Fields] AND "cerebral parenchymal"[Title/Abstract]) OR "parenchymal hemorrhage* cerebral"[Title/Abstract] OR "hemorrhage* intracerebral"[Title/Abstract] OR "hemorrhage* cerebral"[Title/Abstract] OR "cerebral hemorrhage*"[Title/Abstract] OR ("hemorrhage*"[All Fields] AND "cerebral brain"[Title/Abstract]) OR "cerebrovascular accident*"[Title/Abstract] OR "acute cerebrovascular accident*"[Title/Abstract] OR "cva"[Title/Abstract] OR "vascular accident* brain"[Title/Abstract] OR "brain vascular accident*"[Title/Abstract]<br>#6 #4 OR #5<br>#7 pain[MeSH Terms]<br>#8 pain[Title/Abstract] OR Suffering, Physical[Title/Abstract] OR Physical Suffering[Title/Abstract] OR Ache[Title/Abstract] OR Pain[Title/Abstract] OR Arthralgia[Title/Abstract]<br>#9 #7 OR #8<br>#10 #3 AND #6 AND #9	226

Search	Actions	Details	Query	Results	Time
#10	···	>	Search: **((#3) AND (#6)) AND (#9)** Sort by: **Most Recent**	226	10:37:45
#9	···	>	Search: **(#7) OR (#8)** Sort by: **Most Recent**	1,030,174	10:37:02
#8	···	>	Search: **pain[Title/Abstract] OR Suffering, Physical[Title/Abstract] OR Physical Suffering[Title/Abstract] OR Ache[Title/Abstract] OR Pain[Title/Abstract] OR Arthralgia[Title/Abstract]** Sort by: **Most Recent**	865,466	10:36:41
#7	···	>	Search: **pain[MeSH Terms]** Sort by: **Most Recent**	481,197	10:36:21
#6	···	>	Search: **(#4) OR (#5)** Sort by: **Most Recent**	508,576	10:35:32
#5	···	>	Search: **"stroke*"[Title/Abstract] OR "Apoplexy"[Title/Abstract] OR "cerebrovascular apoplexy"[Title/Abstract] OR "apoplexy cerebrovascular"[Title/Abstract] OR "cerebrovascular stroke*"[Title/Abstract] OR "cerebral stroke*"[Title/Abstract] OR "acute stroke*"[Title/Abstract] OR "embolic stroke*"[Title/Abstract] OR "wake up stroke*"[Title/Abstract] OR "ischemic stroke*"[Title/Abstract] OR "thrombotic stroke*"[Title/Abstract] OR "brain ischemia"[Title/Abstract] OR "stroke* cerebrovascular"[Title/Abstract] OR "stroke* cerebral"[Title/Abstract] OR "stroke* acute"[Title/Abstract] OR "brain infarction"[Title/Abstract] OR "cerebral infarct*"[Title/Abstract] OR "intracerebral hemorrhage*"[Title/Abstract] OR "cerebral hemorrhage*"[Title/Abstract] OR "cerebral brain hemorrhage*"[Title/Abstract] OR "hemorrhagic stroke*"[Title/Abstract] OR "hypertensive intracerebral hemorrhage*"[Title/Abstract] OR ("hemorrhage*"[All Fields] AND "Cerebrum"[Title/Abstract]) OR (("Cerebrum"[MeSH Terms] OR "Cerebrum"[All Fields] OR "cerebrums"[All Fields]) AND "hemorrhage*"[Title/Abstract]) OR "cerebral parenchymal hemorrhage*"[Title/Abstract] OR ("hemorrhage*"[All Fields] AND "cerebral parenchymal"[Title/Abstract]) OR "parenchymal hemorrhage* cerebral"[Title/Abstract] OR "hemorrhage* intracerebral"[Title/Abstract] OR "hemorrhage* cerebral"[Title/Abstract] OR "cerebral hemorrhage*"[Title/Abstract] OR ("hemorrhage*"[All Fields] AND "cerebral brain"[Title/Abstract]) OR "cerebrovascular accident*"[Title/Abstract] OR "acute cerebrovascular accident*"[Title/Abstract] OR "cva"[Title/Abstract] OR "vascular accident* brain"[Title/Abstract] OR "brain vascular accident*"[Title/Abstract]** Sort by: **Most Recent**	394,538	10:33:59
#4	···	>	Search: **Stroke OR Apoplexy[MeSH Terms]** Sort by: **Most Recent**	472,690	10:28:59
#3	···	>	Search: **(#1) OR (#2)** Sort by: **Most Recent**	55,077	10:28:15
#2	···	>	Search: **Acupuncture[Title/Abstract] OR Acupuncture Therapy[Title/Abstract] OR Acupuncture Treatment[Title/Abstract] OR Treatment, Acupuncture[Title/Abstract] OR Therapy, Acupuncture[Title/Abstract] OR Manual Acupuncture[Title/Abstract] OR Acupuncture-moxibustion[Title/Abstract] OR Moxibustion[Title/Abstract] OR Moxabustion[Title/Abstract] OR Warm needle[Title/Abstract] OR Warm Acupuncture[Title/Abstract] OR Thermoacupuncture[Title/Abstract] OR Electroacupuncture[Title/Abstract] OR Electro-acupuncture[Title/Abstract] OR electric acupuncture[Title/Abstract] OR Acupuncture Point[Title/Abstract] OR Point, Acupuncture[Title/Abstract] OR Acupoint[Title/Abstract] OR Pharmacopuncture[Title/Abstract] OR Pharmacoacupuncture Treatment[Title/Abstract] OR Treatment, Pharmacoacupuncture[Title/Abstract] OR Pharmacoacupuncture Therapy[Title/Abstract] OR Therapy, Pharmacoacupuncture[Title/Abstract] OR Acupoint injection[Title/Abstract] OR acupuncture injection[Title/Abstract] OR pharmaco-acupuncture[Title/Abstract] OR auricular needle[Title/Abstract] OR Acupunctures, Ear[Title/Abstract] OR Ear Acupuncture[Title/Abstract] OR Ear needle[Title/Abstract] OR earlobe acupuncture[Title/Abstract] OR Auricular Acupuncture[Title/Abstract] OR Auriculotherapy[Title/Abstract] OR Acupuncture, Auricular[Title/Abstract] OR auricular plaster therapy[Title/Abstract] OR auricular point sticking[Title/Abstract] OR Auricular pressure[Title/Abstract] OR Fire needle[Title/Abstract] OR Fire acupuncture[Title/Abstract] OR Acupoint catgut embedding[Title/Abstract] OR Scalp acupuncture[Title/Abstract] OR Scalp needle[Title/Abstract] OR Scalp electroacupuncture[Title/Abstract] OR scalp stimulation[Title/Abstract] OR Eye needle[Title/Abstract] OR Eye acupuncture[Title/Abstract] OR Abdominal acupuncture[Title/Abstract] OR Abdominal needle[Title/Abstract] OR filiform needle[Title/Abstract] OR silver needle[Title/Abstract] OR three-edged needle[Title/Abstract] OR intradermal needle[Title/Abstract] OR Point application[Title/Abstract] OR needle-embedding[Title/Abstract] OR Catgut Embedding[Title/Abstract] OR pricking therapy[Title/Abstract] OR point injection[Title/Abstract] OR Skin Acupuncture[Title/Abstract] OR transcutanclus electrical acupoint stimulation[Title/Abstract] OR TEAS[Title/Abstract] OR electrical acupoint stimulation[Title/Abstract] OR Acupuncture Point[Title/Abstract] OR Acupoint[Title/Abstract]** Sort by: **Most Recent**	44,521	10:27:43
#1	···	>	Search: **Acupuncture OR Acupuncture Therapy OR Moxibustion[MeSH Terms]** Sort by: **Most Recent**	46,299	10:27:06

Web of Science	**#1** Stroke* OR Apoplexy OR Cerebrovascular Apoplexy OR Apoplexy, Cerebrovascular OR Cerebrovascular Stroke* OR Cerebral Stroke* OR Acute Stroke* OR Embolic Stroke* OR Wake up Stroke* OR Ischemic Stroke* OR Thrombotic Stroke* OR Brain Ischemia OR Stroke*, Cerebrovascular OR Stroke*, Cerebral OR Stroke*, Acute OR Brain Infarction OR Cerebral Infarct* OR Intracerebral Hemorrhage* OR Cerebral Hemorrhage* OR Cerebral Brain Hemorrhage* OR Hemorrhagic stroke* OR Hypertensive Intracerebral Hemorrhage* OR Hemorrhage*, Cerebrum OR Cerebrum Hemorrhage* OR Cerebral Parenchymal Hemorrhage* OR Hemorrhage*, Cerebral Parenchymal OR Parenchymal Hemorrhage*, Cerebral OR Hemorrhage*, Intracerebral OR Hemorrhage*, Cerebral OR Cerebral Hemorrhage* OR Brain Hemorrhage*, Cerebral OR Hemorrhage*, Cerebral Brain OR Cerebrovascular Accident* OR Acute Cerebrovascular Accident* OR CVA* OR Vascular Accident*, Brain OR Brain Vascular Accident*	320

	**#2** Acupuncture OR Acupuncture Therapy OR Acupuncture Treatment OR Treatment, Acupuncture OR Therapy, Acupuncture OR Manual Acupuncture OR Acupuncture-moxibustion OR Moxibustion OR Moxabustion OR Warm needle OR Warm Acupuncture OR Thermoacupuncture OR Electroacupuncture OR Electro-acupuncture OR electric acupuncture OR Acupuncture Point OR Point, Acupuncture OR Acupoint OR Pharmacopuncture OR Pharmacoacupuncture Treatment OR Treatment, Pharmacoacupuncture OR Pharmacoacupuncture Therapy OR Therapy, Pharmacoacupuncture OR Acupoint injection OR acupuncture injection OR pharmaco-acupuncture OR auricular needle OR Acupunctures, Ear OR Ear Acupuncture OR Ear needle OR earlobe acupuncture OR Auricular Acupuncture OR Auriculotherapy OR Acupuncture, Auricular OR auricular plaster therapy OR auricular point sticking OR Auricular pressure OR Fire needle OR Fire acupuncture OR Acupoint catgut embedding OR Scalp acupuncture OR Scalp needle OR Scalp electroacupuncture OR scalp stimulation OR Eye needle OR Eye acupuncture OR Abdominal acupuncture OR Abdominal needle OR filiform needle OR silver needle OR three-edged needle OR intradermal needle OR Point application OR needle-embedding OR Catgut Embedding OR pricking therapy OR point injection OR Skin Acupuncture OR transcutanclus electrical acupoint stimulation OR TEAS OR electrical acupoint stimulation OR Acupuncture Point OR Acupoint<br>**#3** pain OR Suffering, Physical OR Physical Suffering OR Ache OR Arthralgia<br>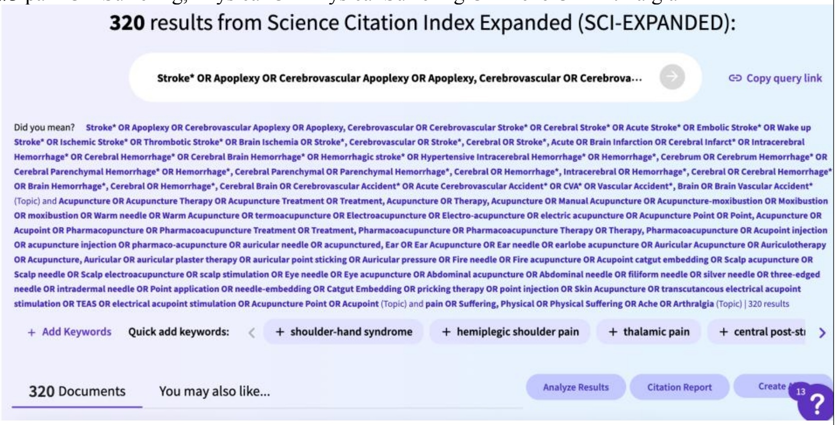 	
Embase	**#1** 'acupuncture'/exp OR 'moxibustion'/exp OR 'acupuncture therapy'/exp OR acupuncture:ti,ab,kw OR 'acupuncture therapy':ti,ab,kw OR 'acupuncture treatment':ti,ab,kw OR 'treatment, acupuncture':ti,ab,kw OR 'therapy, acupuncture':ti,ab,kw OR 'manual acupuncture':ti,ab,kw OR 'acupuncture moxibustion':ti,ab,kw OR moxibustion:ti,ab,kw OR moxabustion:ti,ab,kw OR 'warm needle':ti,ab,kw OR 'warm acupuncture':ti,ab,kw OR thermoacupuncture:ti,ab,kw OR electroacupuncture:ti,ab,kw OR 'electro acupuncture':ti,ab,kw OR 'electric acupuncture':ti,ab,kw OR 'point, acupuncture':ti,ab,kw OR pharmacopuncture:ti,ab,kw OR 'pharmacoacupuncture treatment':ti,ab,kw OR 'treatment, pharmacoacupuncture':ti,ab,kw OR 'pharmacoacupuncture therapy':ti,ab,kw OR 'therapy, pharmacoacupuncture':ti,ab,kw OR 'acupoint injection':ti,ab,kw OR 'acupuncture injection':ti,ab,kw OR 'pharmaco acupuncture':ti,ab,kw OR 'auricular needle':ti,ab,kw OR 'acupunctures, ear':ti,ab,kw OR 'ear acupuncture':ti,ab,kw OR 'ear needle':ti,ab,kw OR 'earlobe acupuncture':ti,ab,kw OR 'auricular acupuncture':ti,ab,kw OR auriculotherapy:ti,ab,kw OR 'acupuncture, auricular':ti,ab,kw OR 'auricular plaster therapy':ti,ab,kw OR 'auricular point sticking':ti,ab,kw OR 'auricular pressure':ti,ab,kw OR 'fire needle':ti,ab,kw OR 'fire acupuncture':ti,ab,kw OR 'acupoint catgut embedding':ti,ab,kw OR 'scalp acupuncture':ti,ab,kw OR 'scalp needle':ti,ab,kw OR 'scalp electroacupuncture':ti,ab,kw OR 'scalp stimulation':ti,ab,kw OR 'eye needle':ti,ab,kw OR 'eye acupuncture':ti,ab,kw OR 'abdominal acupuncture':ti,ab,kw OR 'abdominal needle':ti,ab,kw OR 'filiform needle':ti,ab,kw OR 'silver needle':ti,ab,kw OR 'three-edged needle':ti,ab,kw OR 'intradermal needle':ti,ab,kw OR 'point application':ti,ab,kw OR 'needle embedding':ti,ab,kw OR 'catgut embedding':ti,ab,kw OR 'pricking therapy':ti,ab,kw OR 'point injection':ti,ab,kw OR 'skin acupuncture':ti,ab,kw OR 'transcutanclus electrical acupoint stimulation':ti,ab,kw OR teas:ti,ab,kw OR 'electrical acupoint stimulation':ti,ab,kw OR 'acupuncture point':ti,ab,kw OR acupoint:ti,ab,kw<br>**#2** 'stroke'/exp OR 'Apoplexy'/exp OR stroke*:ti,ab,kw OR apoplexy:ti,ab,kw OR 'cerebrovascular apoplexy':ti,ab,kw OR 'apoplexy, cerebrovascular':ti,ab,kw OR 'cerebrovascular stroke*':ti,ab,kw OR 'cerebral stroke*':ti,ab,kw OR 'acute stroke*':ti,ab,kw OR 'embolic stroke*':ti,ab,kw OR 'wake up stroke*':ti,ab,kw OR 'ischemic stroke*':ti,ab,kw OR 'thrombotic stroke*':ti,ab,kw OR 'brain ischemia':ti,ab,kw OR 'stroke*, cerebrovascular':ti,ab,kw OR 'stroke*, cerebral':ti,ab,kw OR 'stroke*, acute':ti,ab,kw OR 'brain infarction':ti,ab,kw OR 'cerebral infarct*':ti,ab,kw OR 'intracerebral hemorrhage*':ti,ab,kw OR 'cerebral brain hemorrhage*':ti,ab,kw OR 'hemorrhagic stroke*':ti,ab,kw OR 'hypertensive intracerebral hemorrhage*':ti,ab,kw OR 'hemorrhage*, cerebrum':ti,ab,kw OR 'cerebrum hemorrhage*':ti,ab,kw OR 'cerebral parenchymal hemorrhage*':ti,ab,kw OR 'hemorrhage*, cerebral parenchymal':ti,ab,kw OR 'parenchymal hemorrhage*, cerebral':ti,ab,kw OR 'hemorrhage*, intracerebral':ti,ab,kw OR 'hemorrhage*, cerebral':ti,ab,kw OR 'cerebral hemorrhage*':ti,ab,kw OR 'brain hemorrhage*, cerebral':ti,ab,kw OR 'hemorrhage*, cerebral brain':ti,ab,kw OR 'cerebrovascular accident*':ti,ab,kw OR 'acute cerebrovascular accident*':ti,ab,kw OR cva*:ti,ab,kw OR 'vascular accident*, brain':ti,ab,kw OR 'brain vascular	325

	accident*':ti,ab,kw<br>#3 'pain'/exp OR pain:ti,ab,kw OR Ache:ti,ab,kw OR Physical Suffering:ti,ab,kw OR Suffering, Physical:ti,ab,kw OR Arthralgia:ti,ab,kw<br>**#4** #1 AND #2 AND #3<br>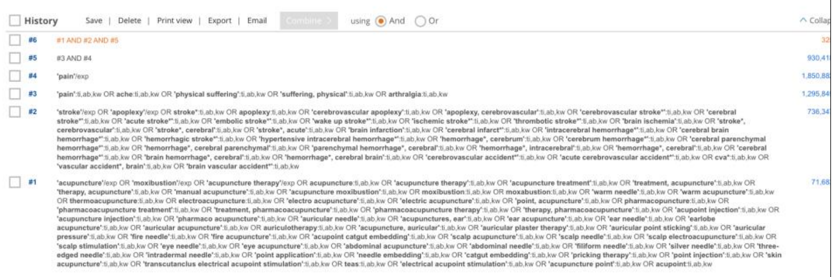	
Cochrane Library	**#1** MeSH descriptor: [Acupuncture] explode all trees<br>**#2** MeSH descriptor: [Acupuncture Therapy] explode all trees<br>**#3** MeSH descriptor: [Moxibustion] explode all trees<br>**#4**(Acupuncture OR Acupuncture Therapy OR Acupuncture Treatment OR Treatment, Acupuncture OR Therapy, Acupuncture OR Manual Acupuncture OR Acupuncture-moxibustion OR Moxibustion OR Moxabustion OR Warm needle OR Warm Acupuncture OR Thermoacupuncture OR Electroacupuncture OR Electro-acupuncture OR electric acupuncture OR Acupuncture Point OR Point, Acupuncture OR Acupoint OR Pharmacopuncture OR Pharmacoacupuncture Treatment OR Treatment, Pharmacoacupuncture OR Pharmacoacupuncture Therapy OR Therapy, Pharmacoacupuncture OR Acupoint injection OR acupuncture injection OR pharmaco-acupuncture OR auricular needle OR Acupunctures, Ear OR Ear Acupuncture OR Ear needle OR earlobe acupuncture OR Auricular Acupuncture OR Auriculotherapy OR Acupuncture, Auricular OR auricular plaster therapy OR auricular point sticking OR Auricular pressure OR Fire needle OR Fire acupuncture OR Acupoint catgut embedding OR Scalp acupuncture OR Scalp needle OR Scalp electroacupuncture OR scalp stimulation OR Eye needle OR Eye acupuncture OR Abdominal acupuncture OR Abdominal needle OR filiform needle OR silver needle OR three-edged needle OR intradermal needle OR Point application OR needle-embedding OR Catgut Embedding OR pricking therapy OR point injection OR Skin Acupuncture OR transcutanclus electrical acupoint stimulation OR TEAS OR electrical acupoint stimulation OR Acupuncture Point OR Acupoint):ti,ab,kw<br>**#5** #1 OR #2 OR #3 OR #4<br>**#6** MeSH descriptor: [Stroke] explode all trees<br>**#7** (Stroke* OR Apoplexy OR Cerebrovascular Apoplexy OR Apoplexy, Cerebrovascular OR Cerebrovascular Stroke* OR Cerebral Stroke* OR Acute Stroke* OR Embolic Stroke* OR Wake up Stroke* OR Ischemic Stroke* OR Thrombotic Stroke* OR Brain Ischemia OR Stroke*, Cerebrovascular OR Stroke*, Cerebral OR Stroke*, Acute OR Brain Infarction OR Cerebral Infarct* OR Intracerebral Hemorrhage* OR Cerebral Hemorrhage* OR Cerebral Brain Hemorrhage* OR Hemorrhagic stroke* OR Hypertensive Intracerebral Hemorrhage* OR Hemorrhage*, Cerebrum OR Cerebrum Hemorrhage* OR Cerebral Parenchymal Hemorrhage* OR Hemorrhage*, Cerebral Parenchymal OR Parenchymal Hemorrhage*, Cerebral OR Hemorrhage*, Intracerebral OR Hemorrhage*, Cerebral OR Cerebral Hemorrhage* OR Brain Hemorrhage*, Cerebral OR Hemorrhage*, Cerebral Brain OR Cerebrovascular Accident* OR Acute Cerebrovascular Accident* OR CVA* OR Vascular Accident*, Brain OR Brain Vascular Accident*):ti,ab,kw<br>**#8** #6 OR #7<br>**#9** MeSH descriptor: [pain] explode all trees<br>**#10** (pain OR Suffering, Physical OR Physical Suffering OR Ache OR Arthralgia):ti,ab,kw<br>**#11** #9 OR #10<br>**#12** #5 AND #8 AND #11	365

	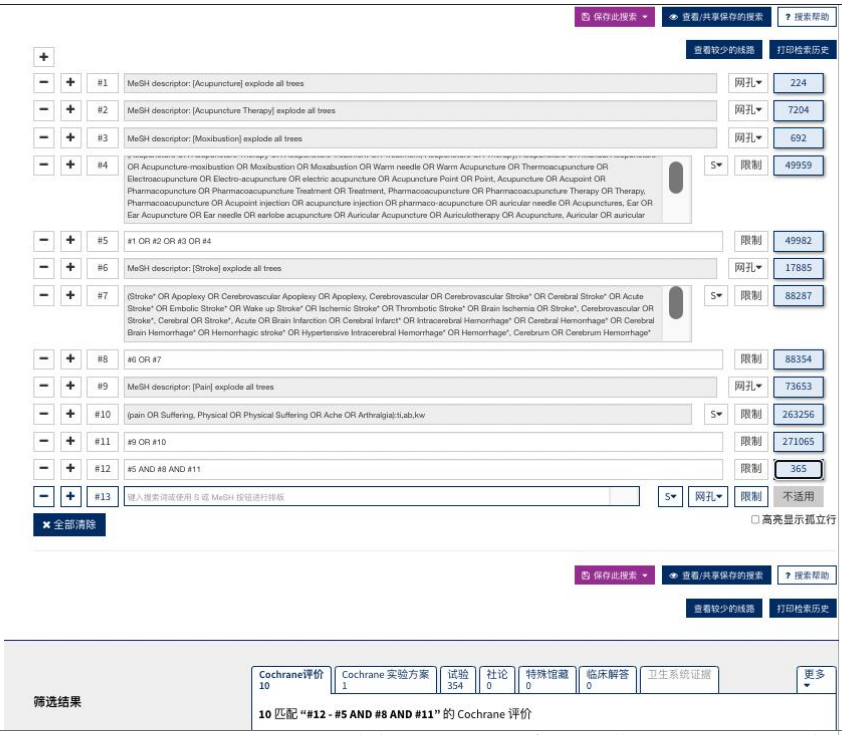	
CNKI	SU%=('针刺'+'电针'+'针灸'+'刺法'+'经皮穴位电刺激'+'隔姜灸'+'隔药灸'+'隔附子饼灸'+'体针'+'耳针'+'头针'+'毫针'+'隔盐灸'+'耳穴贴压'+'热敏灸'+'麦粒灸'+'梅花针'+'穴位敷贴'+'穴位贴敷'+'天灸'+'针刺治疗'+'针灸疗法'+'穴位埋线'+'火针'+'穴位注射'+'艾灸'+'灸法'+'灸疗'+'灸术'+'温针'+'针法'+'三棱针'+'皮肤针'+'芒针'+'眼针'+'手针'+'足针'+'腕踝针'+'平衡针'+'揿针'+'皮内针'+'腹针'+'舌针'+'项针') AND SU%=('中风'+'卒中'+'脑梗死'+'脑梗塞'+'脑出血') AND SU%=('疼痛'+'痛'+'痛症') 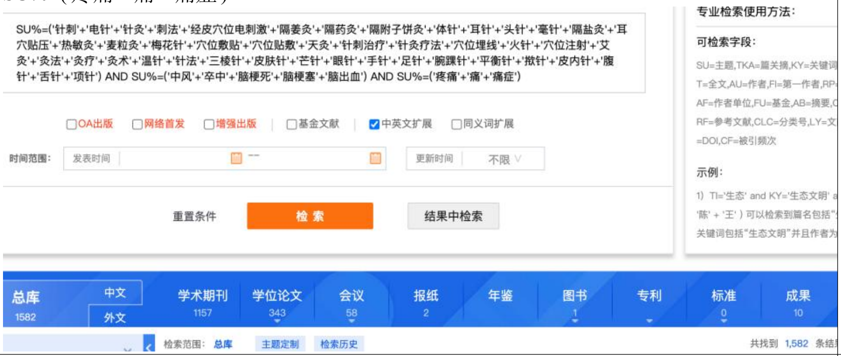	1582
CBM	【快速检索状态】：(针刺 OR 电针 OR 针灸 OR 刺法 OR 经皮穴位电刺激 OR 隔姜灸 OR 隔药灸 OR 隔附子饼灸 OR 体针 OR 耳针 OR 头针 OR 毫针 OR 隔盐灸 OR 耳穴贴压 OR 热敏灸 OR 梅花针 OR 穴位敷贴 OR 穴位贴敷 OR 天灸 OR 针刺治疗 OR 针灸疗法 OR 穴位埋线 OR 火针 OR 穴位注射 OR 艾灸 OR 灸法 OR 灸疗 OR 灸术 OR 温针 OR 针法 OR 三棱针 OR 皮肤针 OR 芒针 OR 眼针 OR 手针 OR 足针 OR 腕踝针 OR 平衡针 OR 揿针 OR 皮内针 OR 腹针 OR 舌针 OR 项针) AND (中风 OR 卒中 OR 脑梗死 OR 脑梗塞 OR 脑出血) AND (痛 OR 疼痛 OR 痛症) 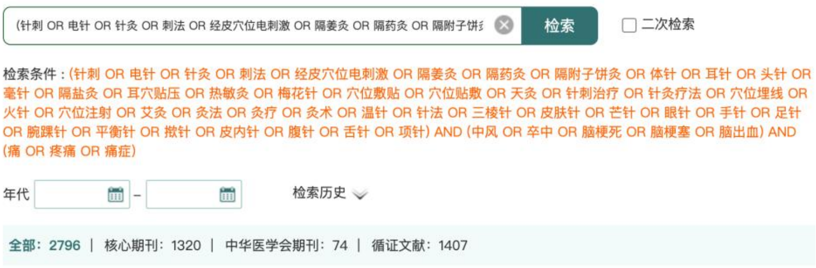	2796

<table>
<tr><td>VIP</td><td>M=(针刺 OR 电针 OR 针灸 OR 刺法 OR 经皮穴位电刺激 OR 隔姜灸 OR 隔药灸 OR 隔附子饼灸 OR 体针 OR 耳针 OR 头针 OR 毫针 OR 隔盐灸 OR 耳穴贴压 OR 热敏灸 OR 梅花针 OR 穴位敷贴 OR 穴位贴敷 OR 天灸 OR 针刺治疗 OR 针灸疗法 OR 穴位埋线 OR 火针 OR 穴位注射 OR 艾灸 OR 灸法 OR 灸疗 OR 灸术 OR 温针 OR 针法 OR 三棱针 OR 皮肤针 OR 芒针 OR 眼针 OR 手针 OR 足针 OR 腕踝针 OR 平衡针 OR 揿针 OR 皮内针 OR 腹针 OR 舌针 OR 项针) AND M=(中风 OR 卒中 OR 脑梗死 OR 脑梗塞 OR 脑出血) AND M=(痛 OR 疼痛 OR 痛症)
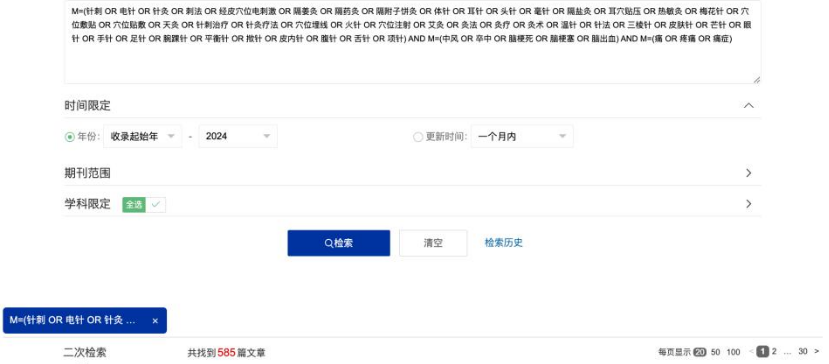
</td><td>585</td></tr>
<tr><td>Wanfang database</td><td>题名或关键词=(“针刺” OR “电针” OR “针灸” OR “刺法” OR “经皮穴位电刺激” OR “隔姜灸” OR “隔药灸” OR “隔附子饼灸” OR “体针” OR “耳针” OR “头针” OR “毫针” OR “隔盐灸” OR “耳穴贴压” OR “热敏灸” OR “麦粒灸” OR “梅花针” OR “穴位敷贴” OR “穴位贴敷” OR “天灸” OR “针刺治疗” OR “针灸疗法” OR “穴位埋线” OR “火针” OR “穴位注射” OR “艾灸” OR “灸法” OR “灸疗” OR “灸术” OR “温针” OR “针法” OR “三棱针” OR “皮肤针” OR “芒针” OR “眼针” OR “手针” OR “足针” OR “腕踝针” OR “平衡针” OR “揿针” OR “皮内针” OR “腹针” OR “舌针” OR “项针”) AND 题名或关键词=(“中风” OR “卒中” OR “脑梗死” OR “脑梗塞” OR “脑出血”) AND 题名或关键词=(“痛” OR “疼痛” OR “痛症 ”)
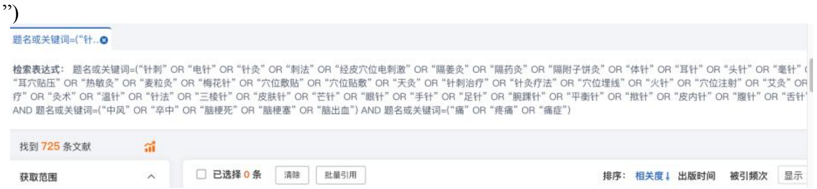
</td><td>725</td></tr>
</table>

Appendix 2. Data Standardization

(1) Standardization of Stroke Type and Disease Duration Data

When extracting stroke type and disease course duration from the included population, if the original text did not specify "Ischemic stroke", "Hemorrhagic stroke" or "Ischemic stroke and hemorrhagic stroke", it was defined as "Not reported".

Stroke stages were categorized into acute phase, recovery phase, and sequelae phase, aligned with the inclusion and exclusion criteria in the articles. If two stages were involved, they were combined.

(2) Standardization of Basic Drug Therapy and Rehabilitation Training

Basic drug treatments for the primary stroke disease in the literature were extracted separately and treated as blank controls.Because rehabilitation training affects pain relief or joint mobility, if basic treatment included rehabilitation training, it was extracted as a separate treatment modality.

(3) Standardization of Multi-Arm Studies

For multi-arm studies, eligible studies were split into pairwise comparisons to assess the effects of acupuncture scientifically.

(4) Extraction Principles for Specific Acupoints in Acupuncture Interventions

When recording acupuncture intervention data, if a study included both specific scalp or auricular acupoints and conventional acupoints, they were extracted as distinct intervention methods.If the study involved electroacupuncture, it was extracted separately and clearly labeled as electroacupuncture.

(5) Guidelines for Acupoint Data Entry

During acupoint data entry, only primary acupoints were recorded, and acupoints related to symptom adjustments were omitted.

If the intervention and control groups involved two different acupuncture therapies, they were split and extracted to form two independent prescriptions, and repeated acupoints within the same study were not double-counted.

The names and locations of all acupoints were standardized based on the "13th Five-Year" national planning textbook "Meridians and Acupoints." For extraordinary points and other special acupoints not included in the textbook, the authors' original records were used as the reference.If colloquial terms such as "Renzhong acupoint" were mentioned in the articles, they were standardized according to the textbook. Tender points, myofascial points, etc., were recorded as per the original text.

Each acupoint entered was recorded in the table by its meridian, acupoint, body part, and specific acupoint category for subsequent analysis. If an acupoint had multiple specific attributes, it was assigned to different categories simultaneously.

(6) Standardization of Outcome Indicator Extraction

They were categorized as follows: a. Symptoms related to the main complaint (if any): such as improvements in neurological deficits, motor function, pain, etc.; b. Disease-associated symptoms (if any): typically the most common accompanying or concurrent symptoms, such as sleep disorders, depression, anxiety, etc.; c. Overall health status: quality of life scales, self-care ability; d. Changes in syndrome patterns (if any): TCM symptom patterns; e. Objective indicators: physical examinations, laboratory tests, imaging studies, etc.; f. Treatment efficacy: effective rate, cure rate, significant improvement rate, etc.; g. Safety and adverse reactions; h. Treatment costs: such as treatment expenses, time costs, transportation costs, etc.; i. Patient expectations and satisfaction; j. Others.

Appendix 3.Detailed table of population characteristics.

Ref. No.	Journal grade	Year	Categories of Pain	Sample size	Types of Stroke	Stroke phase	First-episode	Conventional medicine diagnostic criteria	TCM diagnostic criteria	Imaging diagnostic criteria	Other criteria	Collaboration's risk of bias						
												1	2	3	4	5	6	7
1	1	2024	1	90	3	2	3	Y	N	Y	/	U	U	H	H	L	U	U
2	1	2020	1	46	1	5	3	N	N	Y	/	L	U	U	U	H	U	U
3	1	2016	1	53	3	2	3	N	N	N	/	U	U	H	H	H	U	U
4	1	2023	11	69	4	3	3	N	N	N	/	L	U	U	U	L	U	U
5	2	2013	1	60	3	2	3	Y	N	Y	/	L	U	H	H	L	U	U
6	1	2013	1	30	4	1	3	N	N	N	/	U	L	L	L	L	U	U
7	1	2021	1	34	3	2	2	Y	N	Y	VAS≥4	L	L	U	U	H	L	U
8	1	2015	1	124	3	2	3	N	N	Y	Ashworth≥1	L	U	H	H	H	U	U
9	1	2022	1	60	3	2	3	Y	N	N	/	L	U	U	U	L	U	U
10	5	2020	3	120	3	2	1	Y	Y	Y	/	L	U	H	H	L	U	U
11	5	2018	3	110	4	3	3	N	N	N	/	L	U	H	H	L	U	U
12	5	2019	3	88	1	4	1	N	N	N	Wang Xue-ping, LI Jian-hui, MI Hai-juan. The diagnostic value and treatment of central post-stroke pain [J]. International Journal of Cerebrovascular	L	U	H	H	L	U	U

											Disease, 2016,24 (6):550-554.							
13	5	2018	3	80	4	3	3	N	N	N	/	L	U	H	H	L	U	U
14	3	2020	3	138	4	5	3	N	Y	N	VAS≥4	U	U	H	H	H	U	U
15	3	2015	3	70	4	5	1	N	N	N	VAS≥4	L	U	H	H	H	U	U
16	3	2017	3	70	4	3	1	N	N	N	VAS≥4mm；KlitH, FinnerupNB, JensenTS．Centralpost—strokepain：clinicalcharacteristics，pathophysiology, andmanagement[J]. LancetNeurol，2009，8(9)：857—868	L	U	H	H	H	U	U
17	5	2023	1	104	3	2	3	N	N	Y	/	L	U	H	H	L	U	U
18	4	2015	1	63	3	2	3	Y	Y	Y	Chinese Guidelines for Diagnosis and Treatment of rehabilitation Medicine	H	U	H	H	H	U	U
19	5	2020	1	50	4	2	3	N	N	Y	/	U	U	H	H	L	U	U
20	5	2019	1	80	3	4	2	Y	Y	Y	/	L	U	H	H	H	U	U
21	5	2019	1	40	4	4	3	N	N	N	/	L	U	H	H	H	U	U
22	3	2018	1	60	3	2	2	Y	Y	Y	/	L	U	H	H	H	U	U
23	4	2023	1	60	3	2	3	Y	Y	N	VAS≥5；Constant-Murley≤20	L	U	H	H	H	U	U
24	4	2021	2	60	4	5	3	Y	N	Y	VAS≥4	L	U	H	H	H	U	U
25	2	2023	1	82	3	2	1	Y	N	N	VAS≥4	L	U	H	H	H	U	U
26	5	2022	4	200	1	4	3	Y	N	Y	/	L	U	H	H	L	U	U
27	5	2012	1	129	3	4	1	Y	N	Y	/	U	U	H	H	L	U	U
28	3	2011	1	139	3	2	1	Y	N	Y	/	L	U	H	H	H	U	U
29	5	2008	1	60	4	2	3	Y	N	N	/	U	U	H	H	L	U	U
30	5	2022	4	80	4	2	3	Y	N	Y	/	L	U	H	H	L	U	U

31	4	2015	1	60	3	2	3	Y	Y	N	/	L	U	H	H	L	U	U
32	5	2019	1	80	4	5	3	N	N	N	/	L	U	H	H	L	U	U
33	3	2005	1	60	1	6	3	Y	N	Y	/	H	U	H	H	L	U	U
34	4	2007	1	120	3	1	3	Y	N	Y	/	L	U	H	H	L	U	U
35	5	2021	1	90	1	6	1	Y	N	Y	Diagnostic criteria for shoulder pain after stroke hemiplegia in the Rehabilitation Assessment and Treatment of Stroke	U	U	H	H	H	U	U
36	3	2018	1	142	4	4	3	Y	N	Y	/	L	U	H	H	L	U	U
37	5	2022	1	78	3	4	3	Y	N	Y	/	L	U	H	H	H	U	U
38	5	2006	1	60	1	6	3	N	N	Y	/	U	U	H	H	L	U	U
39	5	2016	1	135	3	3	3	Y	N	Y	/	U	U	H	H	H	U	U
40	3	2016	1	90	3	2	3	Y	N	Y	/	U	U	H	H	H	U	U
41	5	2022	1	74	4	2	1	Y	Y	Y	VAS≥1	L	U	H	H	H	U	U
42	5	2009	1	55	4	2	3	Y	N	N	/	U	U	H	H	L	U	U
43	2	2019	1	60	3	2	3	Y	Y	Y	/	L	U	H	H	L	U	U
44	4	2020	1	60	4	2	1	Y	Y	N	VAS≥4	L	U	H	H	L	U	U
45	5	2013	2	40	3	3	3	N	N	Y	VAS＞6	L	U	H	H	L	U	U
46	3	2021	1	120	1	4	3	Y	N	Y	The stage Ⅰ diagnostic criteria of shoulder-hand syndrome formulated by the Chinese Rehabilitation Medicine Diagnosis and Treatment Standard	L	U	H	H	L	U	U
47	2	2013	1	64	3	2	1	Y	N	Y	Rehabilitation Medicine for Stroke	L	U	H	H	L	U	U
48	2	2014	1	80	4	2	3	Y	Y	Y	/	L	U	H	H	L	U	U
49	3	2018	1	90	4	2	3	N	N	N	/	L	U	H	H	L	U	U

50	2	2017	1	80	3	2	3	Y	N	N	/	L	U	H	H	H	U	U
51	5	2009	8	78	3	2	3	N	N	N	/	U	U	H	H	L	U	U
52	5	2022	1	60	3	2	3	N	N	N	/	U	U	H	H	L	U	U
53	5	2024	1	100	3	2	3	Y	Y	Y	/	L	U	H	H	L	U	U
54	5	2021	1	100	3	6	3	N	N	Y	/	L	U	H	H	H	U	U
55	2	2015	1	80	3	4	3	Y	N	Y	/	L	U	H	H	H	U	U
56	2	2018	2	24	3	2	3	N	Y	Y	VAS≥5	H	U	H	H	L	U	U
57	4	2019	1	60	3	2	1	Y	Y	Y	2016AHA/ASA Guidelines for the Rehabilitation of Adults with Stroke	L	U	H	H	H	U	U
58	3	2013	1	60	3	2	3	Y	N	Y	/	U	U	H	H	L	U	U
59	4	2008	1	36	3	6	3	Y	Y	N	/	L	U	H	H	H	U	U
60	5	2021	3	75	3	6	3	N	N	Y	Diagnostic criteria for central pain after stroke in the Expert Consensus on the Diagnosis and Treatment of Neuropathic Pain	U	U	H	H	L	U	U
61	3	2019	1	120	3	4	3	Y	N	Y	FMA≤54; Diagnostic criteria for post-stroke shoulder pain in the Rehabilitation Assessment and Treatment of Stroke	L	U	H	H	H	U	U
62	5	2021	4	186	4	2	1	N	N	Y	Functional independence score ≥15	L	U	H	H	L	U	U
63	3	2011	6	42	1	2	3	Y	N	Y	/	U	U	H	H	L	U	U
64	5	2022	4	130	4	2	1	N	Y	N	/	L	U	H	H	H	U	U
65	5	2016	1	40	3	4	3	N	N	N	/	L	U	H	H	H	U	U
66	2	2004	1	90	3	6	3	N	N	Y	/	U	U	H	H	L	U	U
67	2	2004	1	90	3	4	3	Y	Y	Y	/	L	U	H	H	L	U	U

68	5	2023	1	55	3	2	3	Y	N	Y	/	L	U	H	H	H	U	U
69	5	2020	1	50	3	2	3	Y	Y	Y	/	U	U	H	H	H	U	U
70	2	2024	3	94	4	6	3	N	Y	N	/	L	U	H	H	L	U	U
71	5	2021	4	100	4	6	1	Y	N	Y	/	L	U	H	H	L	U	U
72	4	2022	6	60	1	2	1	Y	Y	Y	/	L	U	H	H	H	U	U
73	5	2013	1	50	3	2	3	Y	N	Y	/	U	U	H	H	L	U	U
74	2	2019	1	96	4	4	3	Y	Y	Y	/	L	U	H	H	H	U	U
75	5	2024	1	100	3	2	3	Y	Y	N	/	L	U	H	H	L	U	U
76	5	2017	1	60	4	5	3	N	N	N	/	U	U	H	H	L	U	U
77	3	2015	1	120	1	2	3	Y	N	Y	The diagnostic criteria of SHS after stroke was formulated in "The Diagnostic Essentials of Various Cerebrovascular Diseases"	U	U	H	H	H	U	U
78	5	2024	4	62	4	6	3	N	N	N	/	L	U	H	H	H	U	U
79	4	2023	1	63	3	5	3	Y	N	Y	Guidelines for the rehabilitation of stroke in China	L	U	H	H	H	U	U
80	4	2011	1	60	4	2	1	Y	Y	Y	/	L	U	H	H	H	U	U
81	5	2017	1	60	3	4	3	Y	N	Y	/	U	U	H	H	H	U	U
82	3	2012	2	32	3	6	3	Y	Y	Y	VAS≥6	U	U	H	H	L	U	U
83	5	2023	1	48	1	4	3	Y	Y	Y	/	U	U	H	H	L	U	U
84	5	2001	9	194	3	6	3	N	N	N	/	U	U	H	H	L	U	U
85	3	2022	1	74	4	6	3	N	Y	N	/	L	U	H	H	L	U	U
86	2	2013	1	60	3	6	3	Y	Y	Y	/	U	U	H	H	H	U	U
87	3	2017	3	120	3	1	3	Y	Y	Y	/	L	U	H	H	H	U	U
88	5	2021	1	60	4	2	3	Y	Y	N	/	L	U	H	H	L	U	U
89	2	2016	1	80	4	4	3	Y	Y	Y	Hospitalized patients with shoulder pain	L	U	H	H	H	U	U

											according to the diagnostic criteria of the 1995 National Conference on Cerebrovascular Accident							
90	3	2015	1	91	3	2	3	Y	Y	Y	/	L	U	H	H	L	U	U
91	3	2009	2	57	4	5	3	N	N	Y	VAS≥5	L	U	H	H	L	U	U
92	5	2016	1	40	3	2	1	N	Y	Y	/	H	U	H	H	L	U	U
93	4	2022	1	70	1	4	1	Y	N	Y	Chinese Guidelines for Diagnosis and Treatment of rehabilitation Medicine	L	U	H	H	H	U	U
94	5	2022	4	116	4	6	1	N	N	N	/	L	U	H	H	L	U	U
95	5	2023	2	200	3	2	3	Y	N	Y	VAS≥4	L	U	H	H	L	U	U
96	5	2014	5	56	3	2	3	N	N	Y	/	U	U	H	H	L	U	U
97	2	2021	1	39	1	4	1	Y	N	Y	Chinese Guidelines for Diagnosis and Treatment of rehabilitation Medicine	L	L	H	H	L	U	U
98	2	2012	1	80	4	2	3	Y	N	Y	/	H	U	H	H	L	U	U
99	5	2017	1	90	4	4	3	Y	Y	Y	/	U	U	H	H	H	U	U
100	5	2023	1	140	4	2	1	Y	Y	Y	/	U	U	H	H	L	U	U
101	5	2019	1	138	4	4	3	N	N	Y	/	U	U	H	H	H	U	U
102	5	2021	1	64	4	2	3	Y	Y	N	/	U	U	H	H	H	U	U
103	4	2023	1	33	3	2	3	Y	Y	Y	VAS≥3	L	U	H	H	H	U	U
104	2	2022	3	70	4	2	1	N	N	Y	VAS≥3；KlitH, FinnerupNB, JensenTS．Centralpo st—strokepain：clinicalcharacteristics ，pathophysiology, andmanagement[J].	L	U	H	H	H	U	U

											LancetNeurol，2009，8(9)：857—868							
105	5	2022	1	60	1	4	3	Y	N	Y	Applied Clinical Pain Medicine	L	U	H	H	H	U	U
106	5	2015	1	60	3	3	3	N	N	Y	/	L	U	H	H	L	U	U
107	5	2020	1	160	1	4	1	Y	Y	N	/	L	U	H	H	H	U	U
108	5	2015	1	80	3	4	3	N	N	N	/	U	U	H	H	L	U	U
109	4	2023	1	72	4	6	3	Y	Y	Y	VAS≥4	U	U	H	H	H	U	U
110	4	2020	1	60	4	2	3	Y	Y	N	VAS≥2	L	U	H	H	H	U	U
111	5	2020	2	30	3	6	3	Y	N	Y	VAS＞6	L	U	H	H	L	U	U
112	5	2019	1	80	3	6	3	N	N	N	/	U	U	H	H	L	U	U
113	3	2017	1	90	4	2	1	Y	N	Y	/	L	U	H	H	H	U	U
114	5	2022	1	132	4	2	1	Y	N	Y	/	L	U	H	H	H	U	U
115	3	2022	1	200	3	2	3	Y	Y	N	/	L	U	H	H	H	U	U
116	4	2013	1	60	3	2	3	Y	N	Y	/	L	U	H	H	L	U	U
117	5	2014	2	56	4	2	3	N	N	N	/	U	U	H	H	L	U	U
118	5	2021	1	91	3	2	3	N	N	N	/	U	U	U	U	L	U	U
119	2	2023	1	105	3	3	1	Y	Y	Y	VAS≥4	L	U	H	H	H	U	U
120	5	2020	1	120	4	6	3	N	N	N	/	U	U	H	H	L	U	U
121	2	2013	1	60	3	6	3	Y	N	N	/	L	U	H	H	L	U	U
122	2	2011	1	54	1	5	3	N	Y	N	9≥VAS≥6	L	U	H	H	L	U	U
123	5	2014	1	62	3	4	3	N	N	N	/	H	U	H	H	L	U	U
124	5	2015	1	80	4	4	1	Y	Y	Y	/	H	U	H	H	L	U	U
125	5	2014	1	50	3	6	3	Y	N	Y	/	H	U	H	H	L	U	U
126	3	2022	1	88	3	4	3	Y	Y	Y	VAS≥4	L	U	H	H	L	U	U
127	5	2023	1	60	3	4	3	Y	Y	Y	/	L	U	H	H	L	U	U
128	4	2022	3	60	4	5	1	Y	Y	Y	VAS≥4mm；KlitH, FinnerupNB,	L	U	H	H	H	U	U

											JensenTS．Centralpost—strokepain：clinicalcharacteristics，pathophysiology，andmanagement[J]. LancetNeurol，2009，8(9)：857—868							
129	5	2021	1	88	3	6	1	Y	N	Y	Diagnosis of shoulder pain after stroke: referring to the Rehabilitation Medicine of Stroke edited by Wang Maobin in 2006	U	U	H	H	H	U	U
130	3	2011	1	60	3	2	3	Y	N	Y	/	U	U	H	H	L	U	U
131	3	2020	1	180	4	2	3	Y	N	N	/	U	U	H	H	H	U	U
132	2	2001	9	97	3	6	3	N	N	N	/	U	U	H	H	L	U	U
133	3	2022	1	80	3	2	3	Y	Y	N	/	L	U	H	H	H	U	U
134	3	2020	1	68	1	2	1	Y	N	N	/	L	U	H	H	H	U	U
135	5	2021	1	30	4	6	3	N	N	N	/	L	U	H	H	H	U	U
136	3	2021	3	60	4	5	3	Y	N	N	/	L	U	H	H	L	U	U
137	5	2015	1	117	3	4	3	Y	N	N	/	L	U	H	H	L	U	U
138	5	2014	1	60	4	4	3	Y	N	N	Chinese Guidelines for Diagnosis and Treatment of rehabilitation Medicine	U	U	H	H	L	U	U
139	3	2017	1	72	3	2	1	N	Y	Y	/	L	U	H	H	H	U	U
140	2	2008	1	60	3	4	3	Y	N	Y	/	U	U	H	H	L	U	U
141	4	2022	1	100	3	2	3	Y	Y	Y	VAS≥4	L	U	H	H	H	U	U
142	5	2012	2	56	3	2	3	N	N	Y	/	U	U	H	H	L	U	U
143	4	2023	1	63	3	2	1	Y	Y	N	VAS≥4	L	L	H	L	H	U	U
144	5	2014	2	26	3	6	3	N	N	Y	VAS＞6	U	U	H	H	L	U	U

145	5	2022	6	92	1	2	3	N	N	N	/	U	U	H	H	L	U	U
146	3	2019	1	80	3	4	3	Y	N	Y	/	L	U	H	H	H	U	U
147	5	2015	1	60	3	6	3	N	N	N	/	U	U	H	H	L	U	U
148	2	2023	1	75	3	2	1	Y	N	Y	VAS≥4	L	U	H	H	L	U	U
149	5	2023	1	80	4	6	3	N	N	N	/	L	U	H	H	L	U	U
150	5	2021	1	97	4	4	1	N	Y	N	/	L	U	H	H	H	U	U
151	2	1997	2	60	3	6	3	N	N	Y	/	U	U	H	H	L	U	U
152	3	2017	3	120	3	1	3	Y	Y	Y	/	L	U	H	H	H	U	U
153	4	2005	1	90	3	4	3	Y	Y	Y	/	L	U	H	H	L	U	U
154	5	2013	1	60	3	4	1	Y	N	Y	VAS≥4	L	U	H	H	L	U	U
155	5	2023	10	74	4	2	1	N	N	Y	/	L	U	H	H	L	U	U
156	5	2023	1	76	4	6	3	Y	Y	N	/	L	U	H	H	H	U	U
157	3	2014	1	44	3	6	3	Y	N	Y	/	U	U	H	H	L	U	U
158	3	2020	4	72	4	2	3	Y	N	Y	/	U	U	H	H	L	U	U
159	3	2023	1	86	3	2	1	Y	Y	N	/	L	U	H	H	L	U	U
160	5	2022	1	120	3	2	3	N	Y	Y	The diagnostic criteria of hemiplegic shoulder pain in Chinese Rehabilitation Medicine	L	U	H	H	H	U	U
161	3	2022	1	80	1	2	3	Y	Y	N	VAS≥5	L	U	H	H	L	U	U
162	3	2024	1	84	3	5	3	Y	Y	N	/	L	U	H	H	L	U	U
163	3	2014	1	60	3	2	3	Y	Y	Y	/	U	U	H	H	L	U	U
164	2	2020	6	116	1	2	3	Y	N	N	/	L	U	H	H	L	U	U
165	4	2024	1	62	3	2	1	Y	N	Y	VAS≥3	L	U	H	H	H	U	U
166	3	2015	5	80	3	4	3	Y	N	Y	VAS≥ 6	U	U	H	H	L	U	U
167	2	2007	1	98	4	2	3	Y	N	Y	/	U	U	U	U	L	U	U

168	5	2018	1	60	3	2	3	Y	Y	Y	Diagnostic criteria for shoulder pain after stroke hemiplegia in the Rehabilitation Assessment and Treatment of Stroke	H	U	H	H	L	U	U
169	4	2021	1	62	3	2	1	Y	Y	N	VAS > 3	L	L	H	H	L	U	U
170	5	2021	1	60	4	2	1	Y	Y	N	/	U	U	H	H	L	U	U
171	5	2017	1	64	4	1	3	Y	N	Y	The resistance test of biceps brachii was positive	U	U	H	H	H	U	U
172	5	2020	1	60	4	2	3	Y	Y	N	/	L	U	H	H	L	U	U
173	5	2021	1	80	4	6	3	N	N	N	/	L	U	H	H	L	U	U
174	5	2023	1	80	4	6	3	Y	Y	Y	/	L	U	H	H	H	U	U
175	4	2023	1	74	4	6	3	Y	Y	N	/	U	U	H	H	L	U	U
176	3	2020	10	110	4	2	1	Y	Y	N	VAS≥5	L	U	H	H	H	U	U
177	5	2020	1	100	3	2	3	Y	Y	Y	The diagnostic criteria of shoulder-hand syndrome was formulated by Chinese Rehabilitation Research Center in 1996	L	U	H	H	L	U	U
178	4	2018	2	60	3	3	3	N	Y	Y	/	U	U	H	H	H	U	U
179	4	2010	1	30	3	4	3	Y	N	Y	/	L	U	H	H	L	U	U
180	5	2023	6	60	1	6	3	Y	Y	Y	/	L	U	H	H	L	U	U
181	2	2010	1	60	3	2	3	Y	Y	Y	/	U	U	H	H	L	U	U
182	5	2024	1	104	1	4	3	Y	Y	Y	/	L	L	H	H	L	U	U
183	3	2020	1	60	4	4	3	Y	N	N	/	L	U	H	H	H	U	U
184	3	2015	3	62	3	5	3	N	N	Y	VAS≥3	L	U	H	H	H	U	U
185	5	2023	1	108	1	6	1	Y	N	N	/	L	U	H	H	H	U	U
186	3	2018	2	63	3	2	3	N	N	Y	VAS≥4	L	U	H	H	L	U	U

187	5	2018	1	60	3	2	3	Y	N	Y	/	L	U	H	H	L	U	U
188	3	2013	1	80	3	2	3	Y	N	Y	/	U	U	H	H	L	U	U
189	2	2013	1	90	3	2	1	N	N	Y	Wang M B, BryanJO, Young, et al. Neurorehabilitation [M]. Beijing: People's Medical Publishing House, 2009:579-581.	L	U	H	H	L	U	U
190	4	2019	1	74	3	6	1	Y	N	Y	/	L	U	H	H	H	U	U
191	3	2019	1	46	4	4	3	Y	Y	N	/	L	U	H	H	H	U	U
192	2	2011	2	80	3	5	3	N	N	Y	/	H	U	H	H	L	U	U
193	5	2024	1	70	3	2	3	Y	N	N	/	L	U	H	H	H	U	U
194	5	2018	1	90	3	6	2	Y	Y	N	/	L	U	H	H	L	U	U
195	3	2010	1	52	3	2	1	Y	N	Y	/	L	U	H	H	L	U	U
196	4	2022	1	60	3	2	3	Y	N	N	VAS≥3	L	L	U	U	H	U	U
197	5	2018	1	60	3	4	3	Y	N	N	/	L	U	H	H	H	U	U
198	5	2023	1	60	4	2	1	Y	Y	N	VAS≥5	L	U	H	H	H	U	U
199	5	2022	1	60	3	2	3	Y	N	Y	Chinese Guidelines for Diagnosis and Treatment of rehabilitation Medicine	L	U	H	H	L	U	U
200	5	1994	1	61	4	2	3	N	Y	N	/	U	U	H	H	L	U	U
201	5	2021	4	103	2	5	3	N	N	N	/	L	U	H	H	L	U	U
202	4	2008	1	62	4	4	3	Y	Y	N	/	L	U	H	H	L	U	U
203	5	2023	1	60	3	2	1	Y	N	Y	VAS≥3	L	U	H	H	H	U	U
204	5	2009	2	59	3	5	3	N	N	Y	/	L	U	H	H	L	U	U
205	3	2016	1	90	3	2	3	Y	N	Y	/	H	U	H	H	H	U	U
206	2	2007	1	40	3	2	3	Y	N	N	/	H	U	H	H	L	U	U
207	5	2018	1	60	3	2	3	Y	Y	Y	/	L	U	H	H	L	U	U

208	4	2019	1	60	4	2	3	Y	Y	Y	VAS≤6	L	U	H	H	H	U	U
209	5	2024	1	92	3	2	3	Y	N	Y	/	L	U	H	H	L	U	U
210	5	2022	1	60	4	6	3	N	N	N	/	U	U	H	H	H	U	U
211	2	2019	7	96	4	2	3	Y	N	N	/	L	U	H	H	L	U	U
212	5	2014	5	46	4	6	3	N	N	Y	VAS≥ 6	H	U	H	H	L	U	U
213	5	2016	5	200	3	6	3	Y	Y	N	/	L	U	H	H	L	U	U
214	2	2021	1	60	3	4	3	Y	N	Y	/	L	U	H	H	L	U	U
215	4	2014	3	70	4	3	1	N	N	Y	VAS≥4mm；KlitH, FinnerupNB, JensenTS．Centralpo st—strokepain：clinicalcharacteristics，pathophysiology, andmanagement[J]. LancetNeurol，2009，8(9)：857—868	L	U	H	H	H	U	U
216	5	2019	1	60	3	6	3	Y	Y	Y	/	U	U	H	H	L	U	U
217	5	2018	2	60	3	5	3	N	Y	Y	VAS＞6	U	U	H	H	L	U	U
218	2	2019	1	80	4	2	3	Y	Y	N	/	L	U	H	H	H	U	U
219	4	2018	1	60	3	6	3	Y	N	Y	/	L	U	H	H	L	U	U
220	3	2007	1	74	3	6	1	N	N	N	/	U	U	H	H	L	U	U
221	2	2013	1	124	3	6	3	Y	Y	Y	/	L	L	H	U	L	U	U
222	3	2017	1	60	3	2	3	Y	N	N	/	L	U	H	H	L	U	U
223	3	2018	1	60	3	2	1	Y	Y	Y	VAS≥4	L	U	H	H	L	U	U
224	3	2022	1	86	3	2	1	Y	Y	Y	The standard of "stroke shoulder pain" in Rehabilitation Medicine	U	U	H	H	L	U	U
225	3	2022	7	100	1	4	1	Y	N	Y	Brunnstrom＞Ⅰ	U	U	H	H	L	U	U
226	5	2021	1	132	4	6	3	Y	N	Y	Department of Medical Administration,	U	U	H	H	H	U	U

											Ministry of Health, People's Republic of China. Chinese guidelines for the diagnosis and treatment of rehabilitation medicine [M]. Beijing: Huaxia Press, 1999:3-8.]							
227	4	2020	1	60	3	2	3	Y	Y	Y	/	L	U	H	H	L	U	U
228	5	2018	1	280	4	2	3	Y	N	Y	/	L	U	H	H	L	U	U
229	5	2019	3	123	3	1	3	Y	Y	Y	/	U	U	H	H	L	U	U
230	3	2020	2	60	1	2	3	Y	N	Y	/	L	U	H	H	L	U	U
231	5	2017	1	80	3	6	3	N	N	N	/	L	U	H	H	L	U	U
232	5	2024	1	60	4	5	3	Y	Y	Y	/	L	U	H	H	H	U	U
233	4	2018	2	62	3	2	3	N	Y	Y	VAS≥5	U	U	U	U	H	U	U
234	4	2024	2	64	3	5	3	N	Y	Y	VAS≥4	L	U	H	H	H	U	U
235	5	2022	4	92	4	2	1	Y	N	Y	/	L	U	H	H	H	U	U
236	4	2010	5	67	3	2	3	Y	Y	Y	VAS≥ 6	L	L	H	H	H	U	U
237	3	2018	1	94	3	6	3	N	N	Y	The diagnostic criteria of "Chinese Consensus on the Management of severe Cerebrovascular Disease" formulated by Chinese Society of Neurology of Chinese Medical Association and the diagnostic key points of should-hand syndrome formulated by Chinese Rehabilitation Research Center in 2015	L	U	H	H	H	U	U

238	5	2016	1	70	4	2	3	Y	N	Y	/	U	U	H	H	L	U	U
239	4	2005	1	90	1	4	3	Y	N	Y	/	L	U	H	H	L	U	U
240	4	2022	1	72	4	5	3	Y	Y	N	VAS≥4	L	U	H	H	H	U	U
241	5	2022	1	60	4	5	3	Y	Y	N	VAS≥4	L	U	H	H	L	U	U
242	5	2022	1	60	4	4	3	N	N	N	/	L	U	H	H	H	U	U
243	5	2019	5	80	4	2	3	Y	N	Y	/	L	U	H	H	L	U	U
244	5	2021	3	94	3	6	3	Y	N	N	/	L	U	H	H	L	U	U
245	4	2016	1	62	3	4	1	N	N	Y	Brunnstrom: Ⅰ -- Ⅱ stage	L	U	U	U	H	U	U
246	2	2015	1	38	3	2	1	Y	N	Y	/	L	U	H	H	L	U	U
247	5	2016	1	100	4	6	3	Y	N	N	/	U	U	H	H	H	U	U
248	5	2019	1	60	4	6	3	N	N	N	/	U	U	H	H	L	U	U
249	2	2019	1	60	3	5	1	Y	N	Y	/	L	U	H	H	L	U	U
250	4	2011	1	60	3	5	3	Y	Y	Y	/	L	L	H	U	L	U	U
251	3	2016	1	80	3	2	3	Y	N	N	VAS≥5	U	U	H	H	H	U	U
252	5	2006	1	99	3	2	3	Y	N	Y	/	L	U	H	H	L	U	U
253	3	2022	1	120	3	6	1	Y	Y	N	Brunnstrom： Ⅲ～Ⅳ stage	L	U	H	H	L	U	U
254	4	2015	1	60	4	2	3	Y	Y	Y	/	L	U	H	H	H	U	U
255	5	2020	1	30	4	6	3	N	N	N	/	U	U	H	H	L	U	U
256	5	2023	1	82	1	6	3	Y	Y	Y	/	L	U	H	H	L	U	U
257	4	2018	1	60	3	2	3	Y	N	N	/	L	U	H	H	L	U	U
258	5	2012	1	60	3	4	3	Y	N	N	/	L	U	H	H	L	U	U
259	5	2022	1	80	3	4	3	Y	N	Y	/	L	U	H	H	L	U	U
260	2	2018	2	80	4	2	3	N	Y	Y	VAS≥6	L	U	H	H	L	U	U
261	3	2018	1	168	4	6	3	N	N	Y	/	L	U	H	H	H	U	U
262	5	2021	1	80	3	4	3	Y	N	Y	Rehabilitation Medicine for Stroke	U	U	H	H	L	U	U

263	3	2021	1	30	3	6	3	N	N	N	/	U	U	H	H	H	U	U
264	5	2008	1	58	3	2	3	Y	N	Y	/	L	U	H	H	L	U	U
265	2	2017	1	106	3	2	1	Y	Y	Y	The diagnostic criteria of shoulder pain after stroke referred to the "Diagnostic Criteria of shoulder pain after Stroke" in the Rehabilitation Medicine of Stroke	L	U	H	H	H	U	U
266	5	2019	1	80	4	4	3	Y	Y	Y	/	U	U	H	H	H	U	U
267	2	2016	1	60	3	2	3	Y	Y	Y	/	L	U	H	H	L	U	U
268	5	2021	5	96	1	2	3	Y	Y	Y	MAS：I+～IV	L	U	H	H	L	U	U
269	5	2000	8	92	3	6	3	N	N	Y	/	U	U	H	H	L	U	U
270	5	2010	1	90	1	6	3	N	N	Y	/	U	U	H	H	L	U	U
271	2	2011	1	60	4	2	3	Y	N	N	/	L	L	H	H	L	U	U
272	5	2008	1	78	3	2	3	Y	N	Y	/	L	U	H	H	L	U	U
273	4	2013	1	56	4	2	3	Y	N	N	/	H	U	H	H	H	U	U
274	3	2017	1	98	3	2	3	Y	Y	N	Neurorehabilitation Science	U	U	H	H	L	U	U
275	5	2022	1	56	4	2	3	N	N	N	/	U	U	H	H	L	U	U
276	5	2017	1	114	3	4	3	N	N	N	/	U	U	H	H	L	U	U
277	3	2023	2	198	3	3	3	Y	Y	N	/	L	U	H	H	H	U	U
278	3	2012	1	60	3	6	3	Y	N	Y	/	L	L	H	U	L	U	U
279	5	2020	7	90	4	2	3	Y	N	Y	/	L	U	H	H	L	U	U
280	3	2014	2	58	3	5	3	Y	Y	Y	VAS≥7	L	U	H	H	L	U	U
281	5	2013	1	129	3	2	1	Y	N	N	/	U	U	H	H	L	U	U
282	5	2018	1	60	3	2	1	Y	N	Y	/	H	U	H	H	H	U	U
283	4	2017	1	66	3	2	3	Y	N	Y	/	L	L	H	H	H	U	U
284	5	2024	1	66	3	2	1	Y	N	Y	Hemiplegic shoulder pain was diagnosed	L	U	H	H	H	U	U

											according to the criteria in Rehabilitation Medicine							
285	3	2021	1	116	3	2	3	N	Y	N	SHSAS：1 stage	L	U	H	H	H	U	U
286	5	2020	1	60	3	6	3	Y	Y	Y	/	L	U	H	H	H	U	U
287	5	2010	1	60	3	4	3	N	N	Y	/	U	U	H	H	L	U	U
288	4	2010	1	40	3	3	1	Y	N	Y	/	L	L	H	H	L	U	U
289	5	2018	1	60	3	2	3	Y	Y	Y	/	L	U	H	H	H	U	U
290	5	2019	1	30	4	2	3	Y	N	N	/	L	U	H	H	H	U	U
291	3	2013	1	62	1	2	3	Y	N	N	Miao Hongshi. Assessment and treatment of stroke rehabilitation [M1. Beijing: Huaxia Press, 2010. 1996:149-150.	L	U	H	H	L	U	U
292	5	2019	3	80	1	5	3	N	N	Y	/	L	U	H	H	L	U	U
293	4	2019	1	69	3	6	3	Y	Y	Y	Rehabilitation Medicine for Stroke	L	L	H	H	H	U	U
294	5	2016	1	70	3	6	3	Y	N	N	/	U	U	H	H	H	U	U
295	4	2023	1	104	3	2	1	Y	Y	N	/	L	U	H	H	L	U	U
296	5	2023	1	156	3	4	1	Y	N	Y	/	L	U	H	H	H	U	U
297	4	2008	1	40	3	4	3	Y	Y	Y	/	L	L	H	H	L	U	U
298	5	2015	1	80	3	2	3	Y	Y	Y	/	U	U	H	H	H	U	U
299	3	2012	1	60	3	4	3	Y	Y	N	/	H	U	H	H	L	U	U
300	4	2013	1	70	4	2	3	Y	N	N	Wang M B, BryanJO, Young, et al. Neurorehabilitation [M]. Beijing: People's Medical Publishing House, 2009:579-581.	H	U	H	H	L	U	U
301	5	2020	1	120	1	6	1	N	N	N	The supraspinatus tendon rupture test and Speed test were	L	U	H	H	L	U	U

											positive							
302	2	2012	1	40	3	2	1	Y	N	Y	/	U	U	H	H	L	U	U
303	5	2023	1	100	3	2	3	N	N	N	/	L	U	H	H	L	U	U
304	4	2019	1	60	3	2	1	Y	Y	Y	Chinese Guidelines for Diagnosis and Treatment of rehabilitation Medicine	L	U	H	L	L	U	U
305	4	2017	1	60	3	4	1	Y	Y	Y	/	U	U	H	H	L	U	U
306	5	2017	1	60	3	4	1	Y	Y	Y	/	U	U	H	H	L	U	U
307	5	2017	1	86	1	2	3	Y	N	N	/	U	U	H	H	L	U	U
308	5	2009	5	78	4	2	3	N	N	N	/	U	U	H	H	L	U	U
309	5	2021	1	60	4	2	3	Y	Y	N	/	U	U	H	H	L	U	U
310	5	2023	1	48	3	4	3	Y	N	N	/	L	U	H	H	H	U	U
311	5	2016	1	80	3	6	1	N	N	N	/	U	U	H	H	L	U	U
312	3	2024	1	80	3	2	1	Y	Y	N	/	U	U	H	H	L	U	U
313	5	2019	1	60	1	4	1	Y	N	Y	/	H	U	H	H	L	U	U
314	2	2019	1	60	3	4	3	Y	Y	Y	/	L	U	H	H	L	U	U
315	4	2009	1	42	3	4	3	Y	Y	Y	/	L	U	H	H	L	U	U
316	4	2023	1	62	3	2	1	Y	N	N	Guidelines for the rehabilitation of stroke in China	L	L	H	H	L	U	U
317	4	2013	1	90	3	6	3	Y	Y	N	/	L	U	H	H	L	U	U
318	2	2017	1	90	4	2	1	Y	Y	Y	/	L	U	H	H	H	U	U
319	2	2019	6	97	1	2	3	Y	N	N	/	L	U	H	H	H	U	U
320	4	2023	1	60	3	4	1	Y	Y	Y	VAS≥3	L	U	H	H	H	U	U
321	5	2023	1	46	3	6	3	N	N	N	/	L	U	H	H	H	U	U
322	2	2015	12	60	4	2	3	Y	Y	Y	/	U	U	H	H	H	U	U
323	5	2013	1	74	4	2	3	Y	N	N	/	L	U	H	H	L	U	U

324	3	2022	1	102	3	2	3	Y	N	N	VAS＞4	L	U	H	H	H	U	U
325	5	2024	1	40	3	2	3	N	N	Y	/	U	U	H	H	H	U	U
326	3	2002	5	85	3	2	3	N	N	Y	/	U	L	H	H	L	U	U
327	5	2023	4	60	3	6	3	N	N	N	/	U	U	H	H	L	U	U
328	5	2019	1	86	4	6	3	N	N	N	/	L	U	H	H	L	U	U
329	5	2012	1	80	3	5	3	Y	N	Y	/	L	U	H	H	L	U	U
330	2	2023	1	100	3	6	3	Y	Y	Y	/	L	U	H	H	H	U	U
331	4	2018	1	64	4	2	3	Y	N	Y	Rehabilitation of Stroke	U	U	H	H	H	U	U
332	5	2021	1	60	3	2	3	Y	N	Y	VAS＞3	U	U	H	H	L	U	U
333	5	2009	1	80	3	4	3	Y	Y	Y	/	L	U	H	H	L	U	U
334	3	2022	1	60	3	2	3	Y	Y	Y	Jia Zi-shan, LV Pei-yuan, YAN Yan-ning. Rehabilitation of stroke [M]. Hebei: Hebei Science and Technology Press, 2006:240-241.	U	U	H	H	H	U	U
335	5	2014	1	42	3	1	3	Y	Y	N	/	L	U	H	H	L	U	U
336	2	2004	1	90	3	4	3	Y	Y	Y	/	L	U	H	H	L	U	U
337	5	2021	1	60	4	4	1	N	N	N	/	L	U	H	H	H	U	U
338	5	2023	7	60	3	6	3	Y	N	Y	CRPS Ⅰ	L	U	H	H	L	U	U
339	5	2022	1	60	4	4	3	Y	N	N	/	U	U	H	H	L	U	U

NOTE:

The reference NO. are the same as those corresponding to the following references.

Journal grade：1.SCI；2.Chinese Science Citation Database journal；3.Science and Technology/Peking University Core Journal；4.Dissertation；5.Common journal；

Types of Stroke：1.Ischemic；2.Hemorrhagic stroke；3.Ischemic and hemorrhagic stroke；4.Not reported/Unrestricted；

Stroke phase：1.Acute period；2.Recovery period；3.Sequelae period；4.Acute and recovery period；5.Recovery and sequelae period；6.Not reported/Unrestricted staging；

Categories of Pain：1.Shoulder pain；2.Thalamic pain；3.Central pain after stroke；4.Neck and shoulder pain；5.Limb pain；6.Headache；7.Complex regional pain syndrome；8.joint pain；9.Not clearly defined pain；10.Limb and joint pain；11.Chronic neuropathic pain；11.Knee pain；

RCTs references were included in the full text

1 Dong J, Tan W, Chen Y, Kuang Y, Zhang F, Sun Y, et al. The clinical value of Fus subcutaneous needling therapy in improving pain and functional recovery of the affected limb in post-stroke shoulder-hand syndrome. Minerva surgery. 2024.

2 Eslamian F, Farhoudi M, Jahanjoo F, Sadeghi-Hokmabadi E, Darabi P. Electrical interferential current stimulation versus electrical acupuncture in management of hemiplegic shoulder pain and disability following ischemic stroke-a randomized clinical trial. Archives of physiotherapy. 2020;10:2.

3 Lee GE, Son C, Lee J, Lee SH, Lee HJ, Lee KJ, et al. Acupuncture for shoulder pain after stroke: A randomized controlled clinical trial. EUROPEAN JOURNAL OF INTEGRATIVE MEDICINE. 2016;8(4):373-83.

4 Lee JE, Akimoto T, Chang J, Lee HS. Effects of joint mobilization combined with acupuncture on pain, physical function, and depression in stroke patients with chronic neuropathic pain: A randomized controlled trial. PloS one. 2023;18(8):e0281968.

5 Liu S, Shi ZY. Observation on the therapeutic effect of scalp acupuncture and body acupuncture in combination with rehabilitation exercise for hemiplegia and shoulder pain after stroke. World Journal of Acupuncture - Moxibustion. 2013;23(1):21-6.

6 Seo YR, Jung WS, Park SU, Moon SK, Park JM, Park JY. The effect of ouhyul herbal acupuncture point injections on shoulder pain after stroke. Evidence-based complementary and alternative medicine : eCAM. 2013;2013:504686.

7 Sui M, Jiang N, Yan L, Liu J, Luo B, Zhang C, et al. Effect of Electroacupuncture on Shoulder Subluxation in Poststroke Patients with Hemiplegic Shoulder Pain: A Sham-Controlled Study Using Multidimensional Musculoskeletal Ultrasound Assessment. Pain research & management. 2021;2021:5329881.

8 Zhao H, Nie W, Sun Y, Li S, Yang S, Meng F, et al. Warm Needling Therapy and Acupuncture at Meridian-Sinew Sites Based on the Meridian-Sinew Theory: Hemiplegic Shoulder Pain. Evidence-based complementary and alternative medicine : eCAM. 2015;2015:694973.

9 Zhao HB, Lou YB, Zhou T, He J, You X, Yang AR, et al. Moxibustion plus Acupuncture for the Treatment of Poststroke Shoulder Pain: A Randomized Controlled Pilot Study. Complementary medicine research. 2022;29(5):393-401.

10 Yan Hongda, Zheng Liqun, Yang Nan, Zhang Zhiqiang, Sun Chunmei. Treatment of central pain after stroke. A Practical journal of Traditional Chinese Medicine. 2020(5).

11 Qiao Huizi. The effect of acupuncture in patients with central pain after stroke. Contemporary medical theory cluster. 2018;16(24).

12 Qiao Huizi. Caitation acupuncture with TDP irradiation in patients with central pain after cerebral infarction. Journal of the Qiqihar Medical College. 2019;40(21).

13 Qiao Huizi, Dong Anqing. Analysis of the clinical effect of head-point acupuncture for central pain after stroke. Family medicine and medical treatment and drug selection. 2018(07):101-2.

14 Yu Xiaogang, Li Yuxia, Li Wei, Li Shuang, Wang Hao, Zhang Lianghua, et al. Effect of head acupuncture in the treatment of central pain after stroke. Beijing Traditional Chinese Medicine. 2020;39(3).

15 Yu Xiaogang, Wang Hao, Li Wei. Clinical observation of head point acupuncture for central pain after stroke. Journal of Integrated Traditional Chinese and Western Medicine. 2015;13(8).

16 Yu Xiaogang, Wang Hao, Li Wei, Zhang Li. A randomized controlled study of acupuncture for central pain after stroke. Journal of Capital Medical University. 2017;38(1).

17 Fu Gaoyong, Luo Lihu. Effect of electroacupuncture combined with rehabilitation training for hemiplegic shoulder pain after stroke. Chinese and foreign medical research. 2023;21(7).

18 He Junliang, Mr. Clinical study on the treatment of shoulder and hand syndrome pain after stroke [Anhui University of Traditional Chinese Medicine] 2015.

19 He Rulin, Liu Churong, Gao Ruiqi, Wang Zhenghui, Xu Liguo. Acupuncture and extracorporeal shock wave treated 50 cases of shoulder pain after stroke. Scientific health preservation. 2020;23(11):260.

20 He Litao, Su Shengyong, Zou Zhuocheng, Feng Zhuo, Chen Shu. Clinical study on shoulder pain after hemiplegia based on syndrome differentiation of zang and fu meridians. Worlds latest medical information abstract. 2019(28).
21 He Yong. Analysis of the effect of warm acupuncture and penetrating penetration in shoulder pain. Health must read. 2019(20):234.
22 He Yufeng, Zhao Minghua, Wu Jiali, Xian Zuxin, Li Minggao. Clinical observation of shoulder pain after stroke with hand Sanyang and embedded thread. A Practical journal of Traditional Chinese Medicine. 2018;34(08):979-80.
23 He Li Zhu. Clinical study on the treatment of shoulder pain after stroke [Shandong University of Traditional Chinese Medicine] 2023.
24 He Yuxia. Observation of clinical efficacy of acupuncture in treating thalamic pain after stroke [Anhui University of Traditional Chinese Medicine] 2021.
25 Yu Mengting, Li Zhuangmiao, Yan Wenjuan, Liu Fang, Li Xia, Li Xiuxia. Observation of clinical effect of curcumin moxibustion paste on stroke hemiplegic shoulder pain. Chinese acupuncture. 2023;43(12).
26 Hou Yaju, Xu Shouchen. Clinical effects of acupuncture in the treatment of hemiplegic neck and shoulder pain after stroke. Clinical Medicine research and practice. 2022;7(07):147-9.
27 Hou Haiming, Bao Yehua, Wang Yanwu, Chu Jiamei, Zhu Guoxiang, Wang Chong, The clinical observation of electroacupuncture combined with rehabilitation on pain and improvement of upper limb motor function in patients with hemiplegia and shoulder pain after stroke. Chinese Chinese medicine science and technology. 2012;19(1).
28 Hou Haiming, Bao Yehua, Wang Yanwu, Chu Jiamei, Zhu Guoxiang, Wang Chong observed the curative effect of electroacupuncture on hemiplegic shoulder pain after stroke. China Journal of Traditional Chinese Medicine. 2011;29(11).
29 Hou Haiming, Gong Yan, Wang Chong min. Acupuncture Yanglingquan combined with exercise therapy for shoulder pain in 30 cases. Zhejiang Journal of Integrated Traditional Chinese and Western Medicine. 2008;18(8).
30 Guan Yuan, Wang Zheqi, Han Chun, Zhang Zhenfa, Hu Mengyun, Wang Zhiqi. Clinical study of acupuncture treatment for hemiplegic neck and shoulder pain after stroke. Chinese primary medicine. 2022;29(7):999-1002.
31 Feng Song Song. Clinical study of needle stimulation pain point therapy for shoulder pain of shoulder hand syndrome after stroke [Liaoning University of Traditional Chinese Medicine] 2015.
32 Xian Donglian, Chen Xiaoyun, Wang Xiaobo, Huang Liangyan. Clinical effect of acupuncture with microwave for pain in shoulder hand syndrome after stroke. The PLA Journal of Preventive Medicine. 2019;37(7):164-5.
33 Liu Guangting. Effect of giant acupuncture on shoulder pain and hemorrheology after stroke. Journal of Integrated Traditional Chinese and Western Medicine. 2005;3(6).
34 Liu Guanlin. Interventional acupuncture treatment in the acute phase of stroke and its effect on the occurrence of hemiplegia shoulder pain after stroke [Guangxi University of Traditional Chinese Medicine] 2007.
35 Liu Hongye, Zhang Meihua, Ma Fubin. The curative effect of diclofenac diethylamine ultrasonic introduction and deep warm moxibustion on hemiplegia shoulder pain after stroke. Mass technology. 2021;23(5).
36 Liu Yan. Observation on the curative effect of floating needle shock pain point combined with rehabilitation training for stroke and hemiplegic shoulder pain. Hebei traditional Chinese medicine. 2018;40(6).
37 Liu Qiaoyun, Wang Bin. Effect of press therapy and shoulder control training on improving shoulder pain and upper limb function after stroke. Journal of Nantong University (Medical edition). 2022;42(3):277-80.
38 The curative effect of acupuncture combined with rehabilitation techniques in the treatment of shoulder pain after stroke. Chinese disability medicine. 2006;14(1).
39 Liu Hongying, Liu Tao, Wang Zhe. Effect of acupoint injection combined with cupping therapy on the daily living ability of patients with shoulder pain after stroke. Chinese community physicians. 2016;32(27):169-70.
40 Liu Hongying, Fan Zhenlin, Wang Zhe. Efficacy of acupoint injection combined with cupping for shoulder pain after stroke. Jilin Traditional Chinese Medicine. 2016;36(10):1056-8.

41 Liu Lanbing, Wei Zemin, and Fan Hong. Application of press needle combined with rehabilitation therapy in shoulder pain after stroke. Modern and practical medicine. 2022;34(01):69-71.
42 Liu Yumin. Clinical observation of shoulder pain after stroke. Chinese contemporary medicine. 2009;16(22).
43 Bao Yehua, Yuan Jianhui, Le Mengqiao, Fu Qiu, Li Shan in the treatment of shoulder pain after stroke. The Shanghai Journal of Acupuncture. 2019;38(6).
44 Lu Yinfeng. Clinical study of electroacupuncture combined with transcutaneous electrical nerve stimulation in the treatment of shoulder pain after stroke [Anhui University of Traditional Chinese Medicine] 2020.
45 Lu Changjun, Liu Guocheng, Huang Yongjun, Lu Junlei, Zhou Zheyi, Wei Bingxin. Clinical study on the analgesic treatment of thalamic pain. Jilin Traditional Chinese Medicine. 2013;33(2):184-5.
46 Yuan Chen, Zhang Xiaojian, Yuan Jie, Liu Na, Liu Shuqing. Effect of giant thorn mmethod on pain and upper limb function in patients with shoulder hand syndrome after ischemic stroke. The World Journal of Integrated Traditional Chinese and Western Medicine. 2021;16(12):2164-7.
47 Shi Junchao, Ni Huanhuan, Wu Yaozhi, Bao Xinyan, Cui Xiao, Huang Dequan, et al. Floating thorn with stick exercise in the treatment of shoulder pain after stroke. The Shanghai Journal of Acupuncture. 2013;32(12).
48 Ye Bihong, Song Fengjun,, Liu Haifei,, Fang Junhui,. Treatment of shoulder pain after stroke. The Shanghai Journal of Acupuncture. 2014;33(7).
49 Xiang Shan. Effect of acupuncture combined with personalized exercise rehabilitation prescription on spontaneous pain in shoulder hand syndrome and upper limb motor function after stroke. Journal of Integrated Traditional Chinese and Western Medicine. 2018;16(6):699-702.
50 Lv Juan, Ben, easy exhibition. Randomized controlled study of shoulder pain after stroke. Shizhen national medicine. 2017;28(07):1678-80.
51 Lu Anne, Chen Qun. Observation and nursing of acupoint injection in stroke joint pain. Nursing practice and research. 2009;6(19).
52 Lu Zhijuan. Clinical observation of ear point stimulation combined with balance point for hemiplegic shoulder pain after stroke. Chinese Science and Technology Journal Database (full-text version) for Medicine and health. 2022(2).
53 Lu Ruliang. The effect of treating shoulder pain after stroke. Urban and rural enterprise health in China. 2024;39(6).
54 Wu Yaming. Comparison of the effects of massage and acupuncture massage on shoulder pain after stroke and its effect on upper limb function. Reflexology and rehabilitation medicine. 2021;2(13):25-7.
55 Wu Jiayong, Ye Baoye, Xue Kaihua, Huang Saie, Lin Zhicheng, Hong Jiang Cong. The effect of wrist and ankle needle with exercise therapy for stroke shoulder pain. The Shanghai Journal of Acupuncture. 2015;34(05):409-11.
56 Wu Dongdong, Li Yan, Tian Caifang,. Clinical effect of shaoyin meridian puncture method with head acupuncture in thalamic pain after stroke. The Chinese Journal of Traditional Chinese Medicine. 2018;33(2).
57 Wu Hongda. Observation of the clinical efficacy of acupuncture method combined with PNF therapy in treating hemiplegic shoulder pain after stroke [Nanjing University of Traditional Chinese Medicine] 2019.
58 Wu Chuanbi, Jiao Suqin, and No. Effect of acupoint injection on shoulder pain in stroke hemiplegia patients. Chinese rehabilitation. 2013;28(6).
59 Wu Xijun. Clinical study on the efficacy of wrist and ankle needle combined with acupuncture rehabilitation therapy on shoulder pain after stroke hemiplegia [Nanjing University of Traditional Chinese Medicine] 2008.
60 Wu Shuwu, Xin Huaping. Evaluation of the efficacy of pregabalin and acupuncture for central pain after stroke. Jiangxi Medicine. 2021;56(4).
61 Wu Chao, Chen Peng Mengying, Chen Zhibiao. Clinical observation of head needle combined with exercise rehabilitation training for poststroke shoulder pain. Hebei traditional Chinese medicine. 2019;41(4):532-5.
62 Wu Yanrong, Tan Renfei, Li Ming. Efficacy of acupuncture on hemiplegic neck and shoulder pain after stroke. China Practical Medical Journal. 2021;48(16):114-7.
63 Zhou Xia Jia. Clinical study of ear acupuncture in the treatment of headache symptoms after stroke. Journal of Changchun University of Traditional Chinese Medicine. 2011;27(4).

64 Zhou Jiarong, Wu Bingbing. Clinical observation of hemiplegia neck and shoulder pain after stroke. Bright traditional Chinese medicine. 2022;37(07):1245-7.
65 Zhou Xiaoyun, Chen Weiguan. Clinical effect of intradermal embedding needle combined with acupuncture in shoulder pain after stroke. The Grassroots Medical Forum. 2016;20(34):4875-6.
66 Zhou Enhua, Li Yangfan, Deng Baiying, Su Shengyong. Observation of the effects of acupuncture on blood lipid in patients with hemiplegic shoulder pain after stroke. Needle-prick study. 2004;29(4).
67 Zhou Enhua, Su Shengyong, Deng Baiying, Li Yangfan. Recent effects of acupuncture on hemiplegic shoulder pain and its whole blood viscosity after stroke. The Shanghai Journal of Acupuncture. 2004;23(12).
68 Zhou Aizhen, Qiu Feng, Guo Lin, Wang Lei, Liang Guoping. Clinical observation of press needle combined with muscle muscle for for post stroke shoulder pain. A Practical journal of Traditional Chinese Medicine. 2023;39(12):2463-5.
69 Zhou Miao, Hu Caihong, Tang Jie, Wang Xiaoyi, Li Xiaojun, Liu Yajing. Exercise combined with gentle acupuncture to treat shoulder pain after stroke. Health must read. 2018(30):177.
70 Tang Yun, Liu Yilei, Wang Ying, Xie Zongliang, Du Yanyan. Observation of clinical efficacy of needle acupuncture in hemiplegia with central pain: a randomized controlled trial. Needle-prick study. 2024;49(3).
71 Downson, Su Xinhua, Liu Ding. Effect of acupuncture method on hemorrheology and prognosis of hemiplegic neck and shoulder pain after stroke. TCM clinical research. 2021;13(23):95-7.
72 Tang Yan. Observation on the clinical efficacy of treating headache after ischemic stroke based on core prescription acupuncture [Tianjin University of Traditional Chinese Medicine] 2022.
73 Xia Min, Chen Xiaofeng, Cheng Xi. Acupoint application combined with rehabilitation training treated 50 cases of shoulder pain after stroke. Bright traditional Chinese medicine. 2013;28(10).
74 Xia Yang, Duan Yixuan, Zhang Zhaoqing, Luo Xiaojuan, Yin Jing. Randomized controlled study of the efficacy and safety of internal heat needle therapy for shoulder pain after stroke. Needle-prick study. 2019;44(3).
75 Yao Enping, Song Haiyong, Luo Danping, Zhang Mingxing, Shi Yihua, Shan Shari, et al. Wen, Clinical observation of acupuncture combined with modern rehabilitation techniques for shoulder pain after stroke. Chinese folk therapy. 2024;32(16).
76 Yao Guimei cotton, Ge Junling. Thunder-fire moxibustion and acupuncture treated 60 cases of shoulder pain after stroke. Chinese community physicians. 2017;33(18):84-90
77 Yao Lei, Bai Peng. Effect of acupoint injection combined with acupuncture on limb pain and motor ability in post-stroke shoulder-hand syndrome patients. Henan traditional Chinese medicine. 2015;35(5):977-9.
78 Jiang Shumin, Xue Li, Wang Yue Evaluation of acupuncture treatment on pain improvement and prognosis of patients with hemiplegia neck and shoulder pain after stroke. Chinese science and technology journal database (citation version) for medicine and health. 2024(3).
79 Jiang Yiqing. The clinical effect of acupuncture and acupuncture in treating shoulder pain after stroke [Tianjin University of Chinese Medicine] 2023.
80 Kong Lingletter. Clinical study on the treatment of hemiplegia shoulder pain [Shandong University of Traditional Chinese Medicine] 2011.
81 Kong Lin, Li Yifang, Si Shilei. Effect of abdominal acupuncture combined with rehabilitation training on shoulder pain after stroke. Practical medical skills magazine. 2017;24(11):1235-7.
82 Sun Yunguang, Zhang Yan, Lu Changjun, and Liu Guocheng. Clinical observation of 16 cases of thalamic pain after stroke. Shandong Journal of Traditional Chinese Medicine. 2012;31(6).
83 Sun Ningyang, Dong Yuxia, Zhao Manli, Luo Kai, Zhang Lunzhong, LAN Xifu. Treatment of shoulder pain in 24 cases of shoulder pain. Journal of external treatment of Traditional Chinese medicine. 2023;32(3).

84 Sun Wenhua, Zheng Jingrun, Zheng Chunfeng. Analysis of the effect of different acupuncture methods in treating stroke hemiplegia pain. Modern rehabilitation. 2001;5(12S).
85 Sun Wei, Ma Guangxing, Liu Wei, Dai Shuqing, Huan Xin. Clinical study of treatment of hemiplegia shoulder pain after stroke. The Clinical Journal of Acupuncture. 2022;38(5).
86 Sun Hong, Liu Yue. Clinical observation of acupuncture treatment for shoulder pain after stroke. Chinese acupuncture. 2013;33(S1).
87 Sun Ge, Wang Yongkang, Guo Jiakui, Li Li. The effect of central pain after stroke. Beijing Traditional Chinese Medicine. 2017;36(3).
88 Meng Ningning, Mei Cheng, Yangcheng. Efficacy of needle knife for shoulder pain after stroke. Chinese Chinese medicine science and technology. 2021;28(03):493-4.
89 Song Fengjun, Zheng Shili, Zhu Wenzong, Chen Bing, Xi Jingqiao, Zou Xiaojing, et al. The effect of serum IL-6, TNF- α and NO in patients with shoulder pain after stroke. The Shanghai Journal of Acupuncture. 2016;35(10):1158-61.
90 Song Xiuyuan, Dai Shuqing, Su Li, Qu Xianshuang, Liu Bo, Li Xiaoyu, et al. Clinical study of the treatment of shoulder pain after stroke. China Journal of Traditional Chinese Medicine. 2015;33(7).
91 Song Weijian. 29 cases. The Clinical Journal of Acupuncture. 2009;25(10).
92 Song Hujie, Zhang Lei. Clinical effect of abdominal acupuncture therapy combined with traditional acupuncture for shoulder pain after stroke. Medical information (medical and computer applications). 2016;29(34).
93 Yue Taifeng peak. Clinical study on shoulder pain with cerebral infarction 2022.
94 Cui Kai. Clinical effects of acupuncture in the treatment of hemiplegic neck and shoulder pain after stroke. Urban and rural enterprise health in China. 2022;37(06):139-41.
95 Cui Yinglin, Zheng Weifeng. Clinical observation of 100 cases of poststroke thalamic pain. Journal of Gansu University of Traditional Chinese Medicine. 2023;40(2).
96 Cui Xiufang. Efficacy of acupuncture for stroke. Chinese folk therapy. 2014;22(11).
97 Xi Jianming, Wang Huiling, Li Ruiqing, Wang Jing, Wu Mingli, Feng Xiaodong. Efficacy of neuromuscular joint promotion technique combined with floating needle in shoulder subluxation with shoulder pain after stroke. The Chinese Journal of Physical Medicine and Rehabilitation. 2021;43(5).
98 Chang Xiaorong, Liu Wei-ai, Wu Qingming, Fu Lei, Shi Xuehui, Zhang Peng. The effect of combining stroke unit pattern on shoulder pain in patients with shoulder hand syndrome. The Shanghai Journal of Acupuncture. 2012;31(8).
99 Chang Yuesong, Yu Lu. Clinical study of puncture discharge method combined with Chinese medicine to treat shoulder pain after stroke. Chinese prescription drugs. 2017;15(2):112-,3.
100 Kang Zhao, Fang Xingqiang. Clinical observation of Zhaos thunder fire moxibustion to treat shoulder pain after stroke. A Practical journal of Traditional Chinese Medicine. 2023;39(9).
101 Liao Shiying, Xie Qifu. Clinical effect of joint loosening and functional acupuncture in treating hemiplegic shoulder pain. The Journal of Clinical Rational Drug Use. 2019;12(07):149-50.
102 Zhang Shizheng, Chen Xinxin, Huang Jianping, Lin Feifei, Chen Miao. Clinical effect of warm acupuncture and body acupuncture for shoulder pain after stroke. Chinese modern doctors. 2021;59(36):133-7.
103 Zhang Shushan. Observation on the clinical effect of The acupuncture method combined with rehabilitation training in treating shoulder pain after stroke [Beijing University of Chinese Medicine] 2023.
104 Zhang Jinglan, Wang Cui. Effect of injection and drug use on pain perception and sensory thresholds in patients with central pain after stroke. The Shanghai Journal of Acupuncture. 2022;41(10).
105 Zhang Peiqin, Huang Fang, Lai Jasper, Hong Mengying, She Chang. Clinical observation of 30 cases of shoulder pain after stroke. Hunan Journal of Traditional Chinese Medicine. 2022;38(09):66-8+113.

106 Zhang Junfang. Clinical observation of acupuncture on shoulder knee pain and blood viscosity after stroke hemiplegia. Medical frontier. 2015;5(6).
107 Zhang Lina. Effect of acupuncture-assisted rehabilitation therapy on shoulder pain, upper limb function and psychological state in patients with poststroke shoulder hand syndrome. Huaihai Medicine. 2020;38(2):174-6.
108 Zhang Kunhua. Clinical effect of acupuncture combined with rehabilitation training in treating hemiplegic shoulder pain in stroke. Drugs and people. 2015;28(1).
109 Zhang Shaoyang. Observation of clinical efficacy of ultrasound-guided injection combined with electroacupuncture in poststroke shoulder pain [Hunan University of Chinese Medicine] 2023.
110 Zhang Chongyi. Observation of the efficacy of Huangdi internal acupuncture combined with new Bobath technology on hemiplegic shoulder pain after stroke [Guangxi University of Traditional Chinese Medicine] 2020.
111 Zhang Jianquan, Li Xiaoxia. Clinical observation of 15 cases of stroke pain after pain. Hunan Journal of Traditional Chinese Medicine. 2020;36(3).
112 Zhijian Zhang. Analysis of the clinical effects of acupuncture for hemiplegic shoulder pain after stroke. Health must read. 2019(25):98.
113 Zhang Min, He Min. Clinical study on shoulder pain after stroke. Shandong Journal of Traditional Chinese Medicine. 2017;36(3).
114 Zhang Xinting, Hu bridal chamber, Shan Ji Yong, Wang Fuwei. Clinical observation of side acupuncture method combined with local somatosensory neuromuscular promotion therapy for hemiplegic shoulder pain after stroke. A Practical journal of Traditional Chinese Medicine. 2022;38(10).
115 Zhang Mingming, Hao Na, Guoying male, Xiao Wenxia, Li Hongmei. The effects of menstrual remote extraction and wax therapy on pain, upper limb motor function, daily living ability, and serum interleukin 6, tumor necrosis factor α, and nitric oxide levels in patients with shoulder pain after stroke. Hebei traditional Chinese medicine. 2022;44(05):830-3+45.
116 Zhang travel during the Spring Festival travel rush. Clinical study on the treatment of shoulder pain after stroke [Beijing University of Chinese Medicine] 2013.
117 Zhang Xiaorui. Clinical study of electroacupuncture for thalamic pain after stroke. The Worlds Latest Medical Information Abstract (electronic version). 2014(20):23-4.
118 Zhang Hejian, Shen Yihua, Fu Linyi, Jiang Rongan. Effect of acupuncture combined with personalized exercise rehabilitation prescription on spontaneous pain in shoulder hand syndrome and upper limb motor function after stroke. Chinese and foreign womens health research. 2021(1):108-110.
119 Zhang Bo, Ding Yuju, Liu Zhaoyong, Xiao Rong. Clinical observation of stroke hemiplegia shoulder pain. Chinese acupuncture. 2023;43(8).
120 Zhang Xiujing, Cui Chaoyun, Meng Kang. Comparative clinical effectiveness of acupuncture on hemiplegic shoulder pain after stroke. Diet and health care. 2020;7(2):103.
121 Zhang Wei, Zhang Chunyun Mang needle deep stab is the treatment of the shoulder pain after stroke clinical observation. The Shanghai Journal of Acupuncture. 2013;32(12).
122 Zhang Wei, Xue Feiran. The influence of different mild moxibustion on the thermal sensitization of acupoints in patients with shoulder pain after stroke. Journal of Traditional Chinese Medicine. 2011;52(9).
123 Zhang Zheng. Analysis of acupuncture combined with modern rehabilitation for shoulder pain after stroke. Medical information (medical and computer applications). 2014;0(25).
124 Zhang Zouyang, Zhang Mingbo. A randomized parallel controlled study of hemiplegic shoulder pain after stroke. Practical journal of traditional Chinese medicine. 2015;29(11):23-5.
125 Xu Liying, Lin Yijun. Efficacy of acupuncture combined with rehabilitation training on shoulder pain after stroke. Asia-Pacific traditional medicine. 2014;10(7).
126 Xu Junjun. Clinical observation of shoulder pain after stroke with five shoulder points. Henan traditional Chinese medicine. 2022;42(5).
127 Xu Guodong, Ma Han Yi. The effect of acupuncture and PNF technique in treating hemiplegic shoulder pain after stroke. Inner Mongolia Traditional Chinese Medicine. 2023;42(9).
128 Xu Xueyan. Study on the clinical effect of treating central pain after stroke [Guangzhou University of Traditional Chinese Medicine] 2022.

129 Xu Huimin, Li Yuyuan, Wei Ling, Han Runxia. Effect of acupuncture combined with TCM external application for poststroke shoulder pain on patient serum IL-1 β. A Clinical Journal of Traditional Chinese Medicine. 2021;33(02):335-9.
130 Xu Lei, Jiang Yong, Cao Xiaofang, Zhang Xueyun, Shi Weina. Wrist and ankle needle combined with rehabilitation training for hemiplegic shoulder pain. Chinese General Medicine. 2011;9(12).
131 Qi Yan, Sun Yu. Effect of proprioceptive neuromuscular promotion technique and head needle on motor function and pain scores in patients with shoulder pain after stroke. Journal of North Sichuan Medical College. 2020;35(04):679-82.
132 Dai Guohua, Zhang Tong, Shan Yanli. Clinical study of acupuncture for hemiplegia pain secondary to stroke. Journal of Traditional Chinese Medicine. 2001;42(1).
133 Dai Shuqing, Zhu Yunhong, Qu Xianshuang, Yan Liu, and Sun Wei. Comparative study on the influence of large junction method and conventional body injection treatment on shoulder pain treatment effect, nerve function and shoulder motion range after stroke. China Journal of Traditional Chinese Medicine. 2022;40(2).
134 Wen Yichuan, Li Na, Wu Xiangrong. Clinical study of warm needle electroacupuncture with motor therapy and transcutaneous electric nerve stimulation for poststroke hemiplegia shoulder pain. The International Journal of Traditional Chinese Medicine. 2020;42(3):226-30.
135 Fang Peng. The effect of warm acupuncture and penetrating method in rehabilitation treatment of shoulder pain after stroke. Chinese Science and Technology Journal Database (full-text version) for Medicine and health. 2021(9).
136 Cao Yi, Jia Zekun, Pengyuanfeng, Zhou Ting, Yan Yangang, Jiang Na, et al. The curative effect of the needle in the treatment of central pain after stroke. Chinese medicine emergency. 2021;30(7).
137 Cao Jianping, He Songbin, Dai Fangyu, Kou Xuelian, CAI Lisong. Acupuncture and extracorporeal shock wave treated 39 cases of shoulder pain after stroke. Chinese modern doctors. 2015;53(23):88-91.
138 Zeng Scientific, He Qingtang. Efficacy of floating needle therapy on shoulder pain after stroke. Massage and rehabilitation medicine. 2014;5(12).
139 Zhu Yunhong, Ni Shanshan, Dai Shuqing, Ma Guangxing. Clinical observation of hemiplegic shoulder pain after stroke treated with solitary living parasitic soup. Sichuan traditional Chinese medicine. 2017;35(11).
140 Zhu Xianyong. Effect of double acupuncture for shoulder pain after hemiplegia. The Shanghai Journal of Acupuncture. 2008;27(5).
141 Zhu Xiaowei. Clinical observation of external shock wave in "eight shoulder point" [Chengde Medical College] 2022.
142 Zhu Yongqing, Zhang Xiaorui. Clinical observation of electroacupuncture treatment for thalamic pain after stroke. Xinjiang Traditional Chinese Medicine. 2012;30(4).
143 Zhu Honghang. Clinical effect of electroacupuncture in posterior stroke shoulder pain [Tianjin University of Traditional Chinese Medicine] 2023.
144 Zhu Wenbin. Clinical study on the analgesic treatment of thalamic pain. Medical information (medical and computer applications). 2014;0(24).
145 Park Lin. Efficacy of acupuncture for recovering headache during ischemic stroke. The Chinese Medical Journal of the Metallurgical Industry. 2022;39(6).
146 Li Wanlang, Xing Chunyan, Bai Jianzhong, Huang Yunlan, Chen Bingbing. Interactive head needle combined with work therapy for poststroke hemiplegia shoulder pain. The Journal of Traditional Chinese Medicine. 2019;0(8).
147 Li Jiayin, Zhang Mingqiu, Wang Guining. Clinical observation of floating needle therapy with rehabilitation training for shoulder pain after spastic stroke. Medical Information (Xian). 2015(21).
148 Li Zhanbiao, Lv Yunliang, Zhang Zhenyan, Liu Fangming. Efficacy of high-frequency repetitive transcranial magnetic stimulation combined with electroacupuncture in treating hemiplegic shoulder pain after stroke. The Chinese Journal of Physical Medicine and Rehabilitation. 2023;45(4):317-9.
149 Li Juan. Clinical efficacy of acupuncture combined with rehabilitation training for hemiplegic shoulder pain in stroke. Inner Mongolia Traditional Chinese Medicine. 2023;42(06):104-5.
150 Li Pingping, Zhang Liwen, Wang Zhifeng. The effect of host and passenger acupuncture on pain level and upper limb motor function. A Practical journal of Traditional Chinese Medicine. 2021;37(10).
151 Li Yingkun, Jiang Zhenya, Li Changdu. Clinical treatment of the spontaneous thalamic pain after stroke. Journal of Traditional Chinese Medicine. 1997;38(10).

152 Li Yue, Wang Qi, Sun Ge, Sun Xuejiao, Zhang Kaijun, Meng Sasha. Clinical effect of limb movement disorder caused by post-stroke pain. The Clinical Journal of Acupuncture. 2017;33(8).
153 Li Yangfan. Clinical observation of acupuncture on hemiplegic shoulder pain after stroke [Guangxi University of Traditional Chinese Medicine] 2005.
154 Li Yangfan. The curative effect of acupuncture and thunder fire moxibustion on hemiplegia shoulder pain after stroke. Inner Mongolia Traditional Chinese Medicine. 2013;32(26).
155 Li Wenbin. Effect of acupuncture combined with rehabilitation training on limb joint pain after stroke. clinical medicine. 2023;43(1).
156 Li Bin, Wang Kun. The influence of pain pain point moxibustion combined operation therapy on pain symptoms and joint mobility in patients with shoulder and hand syndrome after stroke. Reflexology and rehabilitation medicine. 2023;4(4):164-6,70.
157 Li Ming, Xiao Chunhai. The effect of warm acupuncture and rehabilitation therapy in 22 cases. Hebei traditional Chinese medicine. 2014;36(11).
158 Li Zhi, Zhong Jianguo, Jiang Nan, Wang Fuqing. Analysis of the impact of acupuncture treatment on pain improvement and prognosis in patients with hemiplegic neck and shoulder pain after stroke. Sichuan traditional Chinese medicine. 2020;38(05):191-3.
159 Li Jie, Gao Pan, Li Yonghong, Qiu Xiaoqiong. Effect of acupuncture combined with early acromibrachial rhythm training on pain control, joint movement and quality of life in patients with shoulder pain after stroke. Chinese medicine emergency. 2023;32(12):2142-5.
160 Meng Li. The effect of myosous ultrasound. Modern drug applications in China. 2022;16(15).
161 Li Ran, Liu Shuang, Liu Juntian. Clinical observation of different acupuncture points for hemiplegia shoulder pain after ischemic stroke. Hebei traditional Chinese medicine. 2022;44(10).
162 Li Yan, Cao Xin, Chao Xiaolin. Clinical effect of acupuncture exercise therapy on improving shoulder pain after stroke. China Medical Industry Herald. 2024;21(09):149-52.
163 Li Hong. Acupuncture combined with rehabilitation for post-stroke shoulder pain in 30 cases. Henan traditional Chinese medicine. 2014;34(9).
164 Li Shaokang, Liao Weidong, Zhao Jie, Cao Xiaowen. Effect of acupuncture on plasma endogenous opioid peptides in patients with stroke. Needle-prick study. 2020;45(12).
165 Lee Wei Jane. Observation on the clinical efficacy of acupuncture combined with rehabilitation training in treating shoulder pain after stroke [Hubei University of Traditional Chinese Medicine] 2024.
166 Li Cuie, Ke Juan, Wang Junhua. 40 patients were treated with balanced acupuncture for affected limb pain after stroke. Henan traditional Chinese medicine. 2015;35(3).
167 Li Jing, Chen Li, Mu Rong. The curative effect of acupuncture therapy with different acupoint taking methods combined with rehabilitation training for hemiplegic shoulder pain. The Chinese Journal of Physical Medicine and Rehabilitation. 2007;29(10).
168 Li Lei, Li Lin, Wei Ling, Wu Qiong. Clinical observation of shoulder pain after blood stasis stroke. Chinese drugs and clinical practice. 2018;18(4).
169 Li Beibei. Observation of clinical efficacy of lightning injection and acupuncture in treating shoulder pain after stroke [Shanxi University of Traditional Chinese Medicine] 2021.
170 Li Beibei, Yuan Xiaoling, Yang Invention. Clinical effect of thunder-fire moxibustion and acupuncture on shoulder pain after stroke. Practical medical skills magazine. 2021;28(04):450-453.
171 Li Jiyao, Yu Liwen, Wu Xuanqian, Yi Dan, Ma Yisi, Wu Xiaofei, et al. Clinical study of movement acupuncture and joint loosening in the treatment of shoulder pain after stroke. Integrated Chinese and Western medicine nursing (Chinese and English). 2017;3(12):64-7.
172 Yang Zuoqing, Yuan Haini, Liu Kunming, Lin Linyan. We mainly treated 30 cases of hemiplegia and shoulder pain after stroke. Modern traditional Chinese medicine. 2020;40(1).
173 Yang Lijuan. Acupuncture and extracorporeal shock wave treated 80 cases of shoulder pain after stroke. Chinese science and technology journal database (abstract edition) medicine and health. 2021(10).

174 Yang Xiaofeng, Wang Weiqin, Zhong Jinrui. Effect of acupuncture based on shoulder mobility and quality of life in patients with shoulder pain after hemiplegia of stroke. Inner Mongolia Traditional Chinese Medicine. 2023;42(1):116-7.
175 Yang Xiaorong. Clinical effect of internal heat acupuncture in treating shoulder pain after stroke [Ningxia Medical University] 2023.
176 Yang Zhi, Wang Ting, Gong Yu CD, Tang Mingshan. The inhibitory effect of acupuncture combined with pain rehabilitation on limb joint pain after stroke. The World Journal of Integrated Traditional Chinese and Western Medicine. 2020;15(9).
177 Yang Heng. Study on pain and edema in shoulder and hand syndrome after stroke. Reflexology and rehabilitation medicine. 2020;29(14):60-62.
178 Yang Xianrui. Observation on the clinical efficacy of Suns abdominal acupuncture in improving thalamic pain after stroke [Heilongjiang University of Traditional Chinese Medicine] 2018.
179 Yang Ji. Clinical study on the treatment of poststroke shoulder pain with modern rehabilitation technology [Guangzhou University of Traditional Chinese Medicine] 2010.
180 Lin Jieping, Zheng Xuefeng, Huang Yan. Clinical effect of 60 cases of stroke. Chinese health standard management. 2023;14(17).
181 Bai Qiang. Clinical observation of balance needle for shoulder pain after stroke. Chinese acupuncture. 2010;30(11).
182 Chai Fei, Zou Kun, Chi Zhenhai, Wang Shengling, Wang Peng. Clinical observation of heat-sensitive moxibustion combined with Maitland loosening to treat hemiplegia shoulder pain after stroke. Bright traditional Chinese medicine. 2024;39(07):1361-4.
183 Liang Zhaoming, ancient jianxiong, Chen Xiangmei, Lin Changying. Effect of external shock wave of three-needle shoulder combination on pain degree and motor function in patients with shoulder pain after stroke. Journal of Integrated Traditional Chinese and Western Medicine. 2020;18(09):1375-8.
184 Ou Haining, Zhou Kaixin, Huang Bin, Lu Weiyan, Zhu Junzhi, Qin Zhengjie, et al. Clinical observation of percutaneous acupoint electrical stimulation in the treatment of central neuralgia after stroke. Journal of Integrated Traditional Chinese and Western Medicine. 2015;13(07):881-3.
185 Buyan orchid. Effect of acupuncture combined with massage on pain and joint function in patients with shoulder arthralgia after stroke. Chinese medical innovation. 2023;20(26):80-84.
186 Duan Hongchao, Kong Ying, Wang Yu Mengxin, Wang Di. Clinical observation of "dragon and tiger fighting" acupuncture method for thalamic pain after stroke. Traditional Chinese Medicine Guide. 2018;24(14).
187 Mao Zhifang, Yang Runcheng, Yang Jianhua. Effect of electroacupuncture with rehabilitation training on the recovery of upper limb motor function in patients with shoulder pain after stroke. Chinese Chinese medicine science and technology. 2018;25(2).
188 Tang Zhizhong, Xu Yingle, Yi Jinke, Wang Wenke, Tang Yong. Effect of acupuncture combined with rehabilitation training on shoulder pain and motor function in patients with shoulder-hand syndrome. Shaanxi Traditional Chinese medicine. 2013(7).
189 Wang Jun, Cui Xiao, Ni Huanhuan, Huang Chunshui, Zhou Cuixia, Wu Ji, et al. Treatment of shoulder and hand syndrome and shoulder pain after stroke. Chinese acupuncture. 2013;33(4).
190 Shen Xian. Clinical study of moxibustion therapy for hemiplegia shoulder pain after stroke [Shanghai University of Traditional Chinese Medicine] 2019.
191 Shen Yinli, Wang Jieying. Clinical observation of nursing wrist and ankle needle therapy to relieve pain in patients with shoulder hand syndrome after stroke. Sichuan traditional Chinese medicine. 2019;37(11):211-3.
192 Hong Jue, Lu Ming, Zhu Zhiqiang. Clinical study of acupuncture for the treatment of thalamic pain Journal of Acupuncture and Tuina Science. 2011; 9 (2).
193 Wen Shake, Fan Jiangping, Huang Nanhai. Clinical observation of heat-sensitive moxibustion combined with Bobath therapy for treating hemiplegic shoulder pain after stroke. A Practical journal of Traditional Chinese Medicine. 2024;40(06):1203-5.
194 Wen Yongtang, Liu Yuanbiao. Clinical observation of floating needle therapy combined with Rood physical technique for hemiplegic shoulder pain. Zhejiang Journal of Integrated Traditional Chinese and Western Medicine. 2018;28(11).

195 Pan Sulan, Zhang Chong, Deng Qiulan. Observation and nursing of moxibustion and rehabilitation training in stroke hemiplegia shoulder pain. Liaoning Journal of Traditional Chinese Medicine. 2010;37(5).
196 The bear has no regrets. Clinical effect of electroacupuncture in treating shoulder pain after stroke [Guangzhou University of Chinese Medicine] 2022.
197 Wang Wanhong, Sun Wenyu, Bi Hongyan, Xu Mengting, Chen Yuxiao, Hao Shijie. Tongyuan medicine moxibustion combined with conventional rehabilitation training to treat shoulder pain after stroke. Journal of Gansu University of Traditional Chinese Medicine. 2018;35(06):73-6.
198 Wang Dongsheng, He Lizhu. Clinical observation of treating shoulder pain after stroke. Chinese traditional Chinese medicine science and technology. 2023;30(02):358-61.
199 Wang Lihua, Hu Xiaoyong, Guo Baozhen, LAN Dexiang, Gong Wei. Clinical efficacy study of floating needle combined with intramuscular patch in treating poststroke shoulder pain. Chinese and foreign medical treatment. 2022;41(33).
200 Wang Wei, Qin Aiguo. The analgesic effect of giant thorn method in the treatment of shoulder hand syndrome. Guangxi Traditional Chinese Medicine. 1994(05):6+9.
201 Wang Xiuhua, Guo Xingzhuo, Yan Jingjia. Effect of acupuncture on pain level and daily living ability in patients with lateral neck and shoulder pain after stroke. The Chinese Medicine Guide. 2021;19(32):24-6.
202 Wang Xing. Clinical study of the treatment of shoulder pain after stroke [Guangzhou University of Chinese Medicine] 2008.
203 Wang Hua, Sun Shuqian, Huang Zhe, Zhang Yuanyuan. Clinical effect of acupuncture combined with conventional rehabilitation training for shoulder pain. Massage and Rehabilitation medicine. 2023;14(01):15-7.
204 Wang Weiqiang, CAI Limei, Wei Ling. Treatment of thalamic pain. A Practical journal of Traditional Chinese Medicine. 2009(12).
205 Wang Zhe, Li Bing, and Liu Hongying. Effect of acupoint injection combined with cupping on shoulder mobility in patients with shoulder pain after stroke. Journal of Changchun University of Traditional Chinese Medicine. 2016;32(06):1211-3.
206 Wang Rujie. Acupuncture treated shoulder pain after cerebral infarction. Chinese acupuncture. 2007;27(12).
207 Wang Xiaoyin, Liu Jiaxin, Wen Xi, Liu Tong, Zeng Scientific. Floating needle combined with rehabilitation training for shoulder pain after stroke. Modern drug applications in China. 2018;12(12).
208 Wang Yan. Clinical effect of shoulder pain after stroke [Heilongjiang University of Traditional Chinese Medicine] 2019.
209 Wang Zhihua. Observation of acupuncture on shoulder pain after stroke. Grassroots TCM. 2024;3(03):51-55.
210 Wang Enjiang, Cao Minghui. Observation of the effect of acupoint embedding combined with shoulder pain after stroke. Chinese rural medicine. 2022;29(13):21-3.
211 Wang Wenyi, Wan Fu Ming, Ding Shuqiang. Clinical observation of "three-needle therapy" combined with the treatment of complex regional pain syndrome after stroke. Chinese acupuncture. 2019;39(12):1262-6.
212 Wang Zhimeng, Huo Yin Cheng, Qin Xiaoyong. Clinical observation of balance needle treatment for affected limb pain after stroke. Chinese folk therapy. 2014;22(1).
213 Wang Mei. Effect of traditional Chinese medicine acupoint application on treating limb joint pain in stroke disease. Chinese Chinese medicine science and technology. 2016;23(1).
214 Wang Xin, Li Hu, Lu Xiuyan. Effect of floating injection perfusion therapy combined with joint loosening on hemiplegic shoulder pain in stroke. Journal of Rehabilitation Journal. 2021;31(2).
215 Wang Hao. Clinical observation of acupuncture for the treatment of central pain after stroke [Beijing University of Chinese Medicine] 2014.
216 Wang Lin, Wang Enlong. Clinical observation of acupuncture with rehabilitation training for shoulder pain after stroke. Yunnan Journal of Traditional Chinese Medicine. 2019;40(8):57-8.
217 Wang Shumin. The curative effect of acupuncture and stellate ganglion in thalamic pain after stroke. The Journal of Traditional Chinese Medicine. 2018;46(4).
218 Wang Man, Zhang Zhilong, Wang Xu, Ji Xuequn, Yang Yuanqing, Lu Xuan, et al. Clinical study of the treatment of shoulder pain after stroke. Needle-prick study. 2019;44(08):605-9+19.

219 Wang Can. Evaluation of clinical efficacy of fire needle positive reaction point in treating shoulder pain after stroke [Guangzhou University of Chinese Medicine] 2018.
220 Wang Zujie. Clinical observation of electroacupuncture with exercise therapy for stroke hemiplegia shoulder pain. The Clinical Journal of Acupuncture. 2007;23(3).
221 Wang Xiangyu, Nie Wenbin, Zhao Hong, Yang Su, Meng Fanying, Zhang Liping. The effect of warm acupuncture in stroke spastic shoulder pain. The Shanghai Journal of Acupuncture. 2013;32(6).
222 Wang Mi, Gong Zunke, Wang Shiyan, Yan Wenguang. Clinical efficacy of acupuncture combined with low-frequency repetitive rTMS for shoulder pain after stroke. The Journal of Clinical and Pathology. 2017;37(09):1874-9.
223 Wang Mi, Su Caixia, Yao Rui, Gong Zunke, Wang Shiyan. Clinical observation of abdominal needle combined with repeated transcranial magnetic stimulation for hemiplegic shoulder pain. China Medical Industry Herald. 2018;15(12).
224 Wang Xianlong, Zhao Changying, Sun Jian. Effect of acupuncture combined functional training on upper limb motor function and blood rheology in patients with shoulder pain after stroke. Journal of Changchun University of Traditional Chinese Medicine. 2022;38(09):1015-8.
225 Wang Chao, Wang Wei, Liao Huixiong, Shen Mei. Effect of interactive floating needle combined with virtual reality technology on patients with type I regional pain syndrome in ischemic stroke. Journal of Integrated Traditional Chinese and Western Medicine. 2022;20(14).
226 Wang Xue, Zhang Fangfang, Ma Qing, Lu Yu, Zhang Liang. Efficacy and tolerability of eye needle combined with exercise therapy for stage I pain in shoulder hand syndrome after stroke and its effect on the expression of relevant inflammatory mediators. Practical journal of traditional Chinese medicine. 2021;35(09):41-4.
227 Wang Jianan. Clinical study of abdominal needle combined with Bobath therapy in the treatment of hemiplegic shoulder pain after stroke [Guangxi University of Traditional Chinese Medicine] 2020.
228 Wang Ying, Yang Yuntao, Wu Yiming, Pan Ran, Li Saisai. Clinical observation of balance needle with external needle for shoulder pain after stroke. Chinese folk therapy. 2018;26(12).
229 Wang Xin. Effect of the acupuncture method on the recovery of motor dysfunction caused by central pain after stroke and serum IL-6 and TNF- α levels. The International Medical and Health Guide. 2019;25(9).
230 Wang Hongdu, Jiang Hui. Exploring the clinical effect of acupuncture method on thalamic pain after cerebral infarction. China Journal of Traditional Chinese Medicine. 2020;38(3).
231 Zhen Xiaoran. Analysis of the clinical effects of acupuncture for hemiplegic shoulder pain after stroke. Worlds latest medical information abstract. 2017;17(95):261-2.
232 Tian Peiliang. Clinical observation of umbilical cord needle "thunder hydrolysis" combined with rehabilitation training for shoulder pain after stroke. Chinese folk therapy. 2024;32(06):45-8.
233 Tian Caifang. Clinical effect of head needle plexus acupuncture in stroke [Heilongjiang University of Traditional Chinese Medicine] 2018.
234 Tian Jingwen. Clinical observation of electroacupuncture on poststroke posterior thalamic pain [Heilongjiang University of Traditional Chinese Medicine] 2024.
235 Sheng Li. To investigate the clinical effect of acupuncture in the treatment of hemiplegic neck and shoulder pain after stroke. Diabetes world. 2022;19(8):66-7.
236 Qin Wenyi. Clinical study on treating pain in limbs after qi deficiency and blood stasis in stroke [Chengdu University of Traditional Chinese Medicine] 2010.
237 Cheng Wei, Lu Song. The effect of acupuncture and shoulder hand syndrome and its effect on limb function and pain. Sichuan traditional Chinese medicine. 2018;36(8):183-6.
238 Dou Zhengchuan. Efficacy of acupuncture and rehabilitation training to improve pain and edema in shoulder-hand syndrome after stroke. Massage and Rehabilitation medicine. 2016;7(20):29-30.
239 Su Shengyong. Clinical study of hemiplegic shoulder pain after stroke and quality of life [Guangxi University of Chinese Medicine] 2005.
240 Ji Yiping. Clinical study of acupuncture exercise therapy treatment of stroke shoulder pain [Guangzhou University of Chinese Medicine] 2022.
241 Ji Yiping, Wu Rong, Lu Xia, Liu Yue. Clinical observation of acupuncture exercise therapy for poststroke shoulder pain. Journal of Guangzhou University of Traditional Chinese Medicine. 2022;39(08):1819-23.

242 Luo Yuanpu, to love. Efficacy of three-needle shoulder combination with external shock wave for shoulder pain after stroke and its effect on patients exercise ability. Contemporary medical theory cluster. 2022;20(12):188-90.
243 Luo Yueqin, Zhu Jie wrist and ankle injection on patients with stroke sequelae and limb pain. TCM clinical research. 2019;11(7).
244 Luo Xiaolan, Xue Yingcheng, Xu Haipeng, Wang Meina. A randomized controlled study of acupuncture in patients with pain after central stroke. Journal of external treatment of Traditional Chinese medicine. 2021;30(5).
245 Luo Jinfa. Observation of the efficacy of acupuncture combined with PNF technology in the treatment of shoulder pain after stroke [Anhui Medical University] 2016.
246 Luo Jinfa, Jia Lei, Ni Chaomin. Efficacy of acupuncture combined with proprioceptive neuromuscular promotion technique for shoulder pain after stroke. The Chinese Journal of Rehabilitation Medicine. 2015;30(12):1284-1285.
247 Luo Xia, Liu Lianqiu, Sun Xiaoyan. Clinical study on the treatment of wrist and ankle needle with rehabilitation therapy for shoulder pain after stroke. Chinese disability medicine. 2016;24(15):58-9.
248 Weng Pingxuan, Chen Jinbo, Zheng Jinli. Efficacy of acupuncture and exercise therapy for shoulder pain after stroke hemiplegia. Shenzhen Journal of Integrated Traditional Chinese and Western Medicine. 2019;29(11):42-3.
249 Zhai Hongyu, Xun Yajing, Yan Qiwen, Liu Peijun, Zhan Yan, Lei Debao. Treatment effects of electricity for hemiplegic shoulder pain: A single-blind randomized controlled trial. The Chinese Journal of Integrated Traditional Chinese and Western Medicine. 2019;39(11).
250 Nie Wenbin. Observation of the curative effect of warm acupuncture in treating stroke spastic shoulder pain [Chinese Academy of Chinese Medical Sciences] 2011.
251 Nie Wenbin, Pang Xiuhua, Han Chunjie, Zhang Liping, Li Shoran, Liu Shiju, et al. Clinical research on the treatment of shoulder pain after stroke by comprehensive tendon moxibustion. Traditional Chinese Medicine Guide. 2016;22(16):58-9+62.
252 Xiao Qingyun. Clinical effect of acupuncture and wire embedding in treating hemiplegic shoulder pain after stroke. Modern distance education of Traditional Chinese medicine in China. 2006;4(12).
253 Hu Hongping, Lin Jun, Che Dewen. The curative effect of Dong shiqi acupoint and balanced needle combined with modified Chinese medicine hard paste in patients with shoulder pain after stroke. The World Journal of Integrated Traditional Chinese and Western Medicine. 2022;17(10):1989-92,96.
254 Hu Pan side. Clinical observation of four points in treating shoulder pain after stroke [Heilongjiang University of Traditional Chinese Medicine] 2015.
255 Hu Ruosong. Observation of the efficacy of electroacupuncture in patients with hemiplegic shoulder pain. Friends of the Health. 2020(1):130-1.
256 Su Tongsheng, Ma Rulong, Song Qinqin. 41 patients treated shoulder pain after stroke. Modern traditional Chinese medicine. 2023;43(5).
257 Su Caixia. Clinical study on abdominal acupuncture combined with high-frequency repetitive transcranial magnetic stimulation in the treatment of hemiplegia shoulder pain after stroke [Nanjing University of Traditional Chinese Medicine] 2018.
258 Su Jiafu, Zhuang Chuiga, Ruan Chuanliang, in the treatment of shoulder pain after stroke hemiplegia in 30 cases. Fujian Traditional Chinese Medicine. 2012;43(4).
259 Fan Jianzhong, Zhang Junkun, Yin Ruixue. Efficacy of electroacupuncture combined with new Bobath technology in treating shoulder pain after stroke. Hainan Medicine. 2022;33(1).
260 Meng Lu, Lu Ming, Luo Lingzhi. Clinical observation of 40 cases of poststroke thalamic pain combined with scalp needle electrical stimulation. Journal of Traditional Chinese Medicine. 2018;59(10).
261 LAN Dexiang, Yu Jing. Effect of embedded needle treatment for shoulder pain after stroke hemiplegia. Journal of Bengbu Medical College. 2018;43(6).
262 LAN Dexiang, Wang Lihua, Guo Baozhen, Wang Jun. Randomized controlled study of the efficacy of floating needle combined with intramuscular patch in patients with shoulder pain after stroke. Chinese practical medicine. 2021;16(23).
263 Yuan Wenping. To explore the clinical efficacy of acupuncture for hemiplegic shoulder pain after stroke. The Worlds Lest Medical Information Abstract (continuous electronic journal). 2021;21(3):233-4.
264 Yuan Yongchun. 30 cases of hemiplegic shoulder pain after stroke. Traditional Chinese medicine research. 2008;21(4).

265 Yuan Haiguang, Liu Zhibin, Feng Weixing. Treatment of shoulder pain after stroke. Chinese acupuncture. 2017;37(10):1035-9.
266 Yuan Wei, He Litao, Chen Shu, Su Shengyong, Huang Yixiang. Effect of acupuncture based on the quality of life in patients with shoulder pain after hemiplegia of stroke. TCM clinical research. 2019;11(3).
267 Xu Shiwen, Yu Chuan, Shen Bin. Effect of different needle retention time on the efficacy of acupuncture for shoulder pain after stroke. The Shanghai Journal of Acupuncture. 2016;35(8).
268 Xu Yanlin. The application of head needle needle in limb spasm with pain after cerebral infarction. Bright traditional Chinese medicine. 2021;36(6).
269 Xu Bin, Zhang Yuanling, Li Peifang. Skin needle percussion and traditional Chinese medicine damp and heat compress treated stroke joint swelling and pain in 48 cases. Chinese folk therapy. 2000;8(6).
270 Xu January. Efficacy of needle tank and TDP on hemiplegic shoulder pain after cerebral infarction. Qinghai Medical Journal. 2010;40(1).
271 Xie Renming, Chen Hongxia, He Mingfeng Clinical observation of abdominal acupuncture combined with rehabilitation training to treat shoulder pain after stroke. Journal of Nanjing University of Traditional Chinese Medicine. 2011;27(4).
272 Xie Hui, Yao Jiedi, Chen Xiangjun, Yao Jiehui. Clinical effect of acupuncture and embedded needle for hemiplegic shoulder pain after stroke. Chinese practical medicine. 2008;3(4).
273 He Xinze. Clinical study on the treatment of stroke convalescent shoulder pain [Qingdao University] 2013.
274 He Xiaohui, Xue Lifeng, Miao Yuxin, Xu Shengdong, Li Yanming. The effect of floating needle combined with infrared irradiation therapy on improving shoulder pain in elderly patients after stroke. The Chinese Journal of Gerontology. 2017;37(12).
275 Jia Xia. Analysis of acupuncture combined with blood prick therapy for shoulder pain after stroke. Chinese Science and Technology Journal Database (full-text version) for Medicine and health. 2022(12).
276 Lai Lei. Application of acupuncture in the treatment of patients with hemiplegic shoulder pain after stroke. Chinese Science and Technology Journal Database (full-text version) for Medicine and health. 2017(1).
277 Zhao Dong, Chu Jia Xin, Peng Fei, Li Jiaojiao, Cui Runhong, Liang Chunxu. Clinical effect of brain acupuncture combined with repetitive TMS for poststroke thalamic pain. China Medical Industry Herald. 2023;20(32).
278 Zhao Hong, Nie Wenbin. The effect of warm acupuncture in stroke spastic shoulder pain. Sichuan traditional Chinese medicine. 2012;30(3).
279 Zhao Yanyan. Effect of needle retention time on the efficacy of complex regional pain syndrome after stroke. Modern and practical medicine. 2020;32(4).
280 Lu Ming, Zhao Yanxiang. Clinical observation of treating thalamic pain after stroke. The Clinical Journal of Acupuncture. 2014;30(9).
281 Car leather square. Clinical effect of electroacupuncture and rehabilitation for hemiplegic shoulder pain after stroke. Massage and Rehabilitation medicine. 2013(10).
282 Xing Chunyan, Li Wanlang, Ma Fei, Xu Jiawei, Zhan Dongmei. Effect of interactive head needle therapy on hemiplegic shoulder pain. Massage and Rehabilitation medicine. 2018;9(12).
283 Shao Yuling. Observation of the curative effect of three-needle shoulder combined with exercise therapy in treating hemiplegic shoulder pain [Zhejiang Traditional Chinese Medicine University] 2017.
284 Di Furong, Zhao Yi. Clinical efficacy of assisted acupuncture myofascial trigger point under myoseletous ultrasound for hemiplegic shoulder pain in stroke. Journal of Shanxi University of Traditional Chinese Medicine. 2024;25(04):435-9.
285 Zou Zhihong, Wu Yulei, Lu Jian, Wen Jun. The effect of shoulder and hand syndrome on pain and upper limb motor function. The Clinical Journal of Acupuncture. 2021;37(12):19-25.
286 Zheng Liqun, Qi Jin, He Yufeng, Sun Chunmei, Zhang Zhiqiang. 30 cases. Journal of external treatment of Traditional Chinese medicine. 2020;29(03):64-5.
287 Zheng Wenying, Zhao Jinyan. Acupuncture for hemiplegic shoulder pain after stroke. Chinese folk therapy. 2010;18(12).
288 Guo Yuanyuan. Clinical study on the treatment of shoulder pain after stroke [Guangzhou University of Chinese Medicine] 2010.

289 Jin Yu, Wang Fei. Wrist and ankle injection treated shoulder pain after stroke in 30 cases. Journal of external treatment of Traditional Chinese medicine. 2018;27(01):38-9.
290 Zhong, Zhang Suwan, Gao Shenghai. The effect of warm acupuncture and penetrating method in rehabilitation treatment of shoulder pain after stroke. Chinese contemporary medicine. 2019;26(6):117-9.
291 Zhong Fei, Cui Shaoyang, Wang Shuhui, Xu Mingzhu, Lai Xinsheng, Zheng Shenghui shoulder three injections combined with rehabilitation training on the pain and motor function of patients with shoulder and hand syndrome after stroke. Journal of Liaoning University of Traditional Chinese Medicine. 2013;15(11).
292 Yan Jin, Qiao Huizi. Analysis of the curative effect of repeated transcranial magnetic stimulation on central nerve pain after cerebral infarction. Journal of Shenyang Medical College. 2019;21(6).
293 Chen Qian. Clinical effect of acupuncture combined with PNF technology in treating shoulder pain after stroke [Tianjin University of Traditional Chinese Medicine] 2019.
294 Chen Yiwei, You Zhenjuan Clinical observation of ligustrazine acupoint injection in the treatment of hemiplegic shoulder pain in stroke. Chinese Chinese medicine science and technology. 2016;23(4).
295 Chen Wei. Clinical study on hemiplegia shoulder pain with PNF after stroke [Jiangxi University of Traditional Chinese Medicine] 2023.
296 Chen Wei, Yu Hang. The effect of press needle and PNF technique on hemiplegic shoulder pain and its effect on pain level, upper limb function and expression of inflammatory factors. Asia-Pacific traditional medicine. 2023;19(07):87-91.
297 Chen Guozhen. The influence of warm acupuncture combined with rehabilitation training on shoulder pain after hemiplegia [Guangzhou University of Traditional Chinese Medicine] 2008.
298 Chen Zonghua, Huang Zongju, Li Yue. Clinical study on thunder-fire moxibustion combined with massage technique to treat shoulder pain after stroke. A Practical journal of Traditional Chinese Medicine. 2015;31(12):1159-60.
299 Chen Yu Yi, Shi Xuemin. 30 cases of stroke and shoulder pain were treated by giant thorn in peace and three points. The Clinical Journal of Acupuncture. 2012;28(5).
300 Chen Jiahong. Effect of abdominal needle therapy on upper limb function in patients with shoulder pain after stroke [Guangzhou University of Chinese Medicine] 2013.
301 Chen Tingqu. Clinical effect of treating shoulder pain with qi deficiency and blood stasis after stroke and the influence of patients quality of life. TCM clinical research. 2020;12(8).
302 Chen Jianping, Guo Yuanyuan: Clinical research on treating shoulder pain after stroke. The Chinese Journal of Rehabilitation Medicine. 2012;27(3).
303 Chen Bin, Li Jinxiang. Analysis of the influence of acupuncture combined with rehabilitation training treatment on shoulder pain and quality of life in stroke patients. Chinese and foreign medical treatment. 2023;42(01):177-180+189.
304 Chen Mei. Clinical study on the combination of acupuncture and knife healing method in the treatment of shoulder pain after stroke [Jiangxi University of Traditional Chinese Medicine] 2019.
305 Chen Qi. Observation on the efficacy of wrist and ankle acupuncture combined with rehabilitation training in treating shoulder pain after stroke [Nanjing University of Traditional Chinese Medicine] 2017.
306 Chen Qi, Zhang Hongbing. The curative effect of wrist and ankle needle combined with rehabilitation training in the treatment of shoulder pain after stroke. Everyone is healthy. 2017(10):138+280.
307 Chen Qiongzhen. Effect of different needle retention time on the efficacy of acupuncture for shoulder pain after stroke. Modern distance education of Traditional Chinese medicine in China. 2017;15(4).
308 Chen Qun, Anne Lu. Application of hydroneedle therapy in the treatment of limb joint pain after stroke. Journal of the Bethune Army Medical College. 2009;7(2).
309 Chen Saixuan, Hu Jiangbiao. Clinical observation of 30 cases of shoulder pain after stroke. Zhejiang Journal of Traditional Chinese Medicine. 2021;56(5).

310 Chen Xiongjie, Song Xuepeng, Liu Xinxin, Wu Xiaolin, Lai Ming, Zhou Hongchun, et al. Clinical observation of deltoid nerve injection for acute shoulder pain in stroke. A Practical journal of Traditional Chinese Medicine. 2023;39(12):2469-70.
311 Jing Chen. Efficacy of acupuncture and exercise therapy for shoulder pain after stroke hemiplegia. Chinese and foreign womens health research. 2016(09):79-81.
312 Chen Jingwen, Liu Ming, Li Zhihui, Zhang Jing, Wang Qiongfen, Mao Wei, et al. Clinical observation of anti-spasticity acupuncture combined with shoulder blade control training for stroke hemiplegia shoulder pain. Henan traditional Chinese medicine. 2024;44(1).
313 Chen Shun. Clinical effect of electroacupuncture in shoulder arthralgia after ischemic stroke. Asia-Pacific traditional medicine. 2019;15(6).
314 Chen Shunxi, Chen Feiyu, Liu Xiaoping, Bao Yehua, Chu Jiamei, Yuan Jianhui, et al. Impact and correlation analysis of acupuncture acupuncture on shoulder pain and upper limb motor dysfunction after stroke. The Chinese Journal of Rehabilitation Medicine. 2019;34(7).
315 Tao Lin flower. The influence of giant thorn method combined with rehabilitation training on shoulder pain after hemiplegia [Fujian College of Traditional Chinese Medicine] 2009.
316 Lei Qin. Clinical study on the treatment of poststroke shoulder pain with acupuncture of "shoulder pain group" [Gansu University of Traditional Chinese Medicine] 2023.
317 Ju Xiaojing. Clinical study on wrist and ankle acupuncture combined in the treatment of stroke and shoulder pain [Shandong University of Traditional Chinese Medicine] 2013.
318 Han Zhenxiang, Qi Lili, Zhou Yixin, Zhang Hong, Xu Wenjie, Wang Honglin, et al. Clinical observation of improving the motor function and quality of life with PNF technology. The Shanghai Journal of Acupuncture. 2017;36(12):1420-4.
319 Han Jingxian, Dong Hua, Zhao Hongyi, Wang Jianwu. Observation and mechanism study of acupuncture treatment for headache in the recovery period of ischemic stroke. Chinese acupuncture. 2019;39(11).
320 Han Lu. Clinical observation of four-needle shoulder tamping method in the treatment of shoulder pain after stroke [Heilongjiang University of Traditional Chinese Medicine] 2023.
321 Rao Wenhe. The improvement effect of acupuncture and moxibustion combined with periodic resistance training on motor function and pain symptoms in patients with poststroke shoulder and hand syndrome. Heilongjiang Traditional Chinese Medicine. 2023;52(03):93-5.
322 Rao Xiaodan, Liu Yongfeng, Ma Xiaoming, Yan Bing, Gou Yanhua, Zhang Shaoyun Wen acupuncture and knee four points in the treatment of knee pain after stroke (English). World Journal of Acupuncture-Moxibustion. 2015;25(3).
323 Ma Junling. A randomized parallel controlled study of acupuncture for hemiplegia shoulder pain after stroke. Practical journal of traditional Chinese medicine. 2013;27(6).
324 Ma Xiaomin, Shang Guochao, Sun Dunpo, Li Xin, Huang Qian, Zhao Hongyan, et al. The effect of the speed acupuncture method combined with massage on shoulder pain of stroke and ultrasound detection index of lesions. China Journal of Traditional Chinese Medicine. 2022;40(04):139-42.
325 Ma Songwu, Gaoshan, Zhang Cheng, Gao Chong. Rehabilitation training in patients with poststroke shoulder hand syndrome combines the effect of acupuncture treatment and its effect on pain score. Inner Mongolia Traditional Chinese Medicine. 2024;43(6):112-4.
326 Ma Jing, Zhang Wenjun. Wrist and ankle injection treated hemiplegic limb pain in stroke. Clinical rehabilitation in China. 2002;6(9).
327 Huawei Gao, and Mr Zhihong Li. Efficacy of acupuncture in the treatment of hemiplegia neck and shoulder pain after stroke and evaluation of adverse effects in patients. Health guidelines. 2023(5):197-9.
328 Gao Xiaowei. Effect of moxibustion combined with rehabilitation care on joint function and quality of life in patients with shoulder pain after stroke. Bifeet and health care. 2019;28(21).
329 Gao Hanyi, Zhang Zhongxia, Zhang Yan, Yu Tianying. The effect of acupuncture shoulder pain combined with exercise therapy for shoulder pain after hemiplegia. Shandong Medicine. 2012;52(27).

330 Gao Lingai, Han Xiuping, Lu Yanlin, Chen Kehua, Wang Jianzhi. Effect of wheat moxibustion combined with rehabilitation for shoulder pain after stroke. The Shanghai Journal of Acupuncture. 2023;42(08):820-4.
331 Huang Wenting. Clinical effect of acupuncture in treating shoulder pain after stroke [Heilongjiang University of Traditional Chinese Medicine] 2018.
332 Huang Xiaoming, Wen Qingxiu, Li Xiuxia ear acupoint pressure on the sleep quality of patients with shoulder pain after stroke. Contemporary nurses (Zhongxun journal). 2021;28(6).
333 Huang Yong, Su Shengyong, Deng Baiying, Huang Jinjun, Hu Yuying, He Yufeng acupuncture treatment of shoulder pain after stroke hemiplegia in 40 cases of clinical observation. Guangxi Traditional Chinese Medicine. 2009;32(2).
334 Li Huamao, Wang Qin. Effect of acupuncture on pain and comprehensive function in stroke patients with shoulder hand syndrome. Journal of Hubei University of Traditional Chinese Medicine. 2022;24(4).
335 Gong Xiuqin, Zhang Wenjuan moxibustion combined with rehabilitation nursing on joint function and quality of life in patients with shoulder pain after stroke. The International Journal of Nursing. 2014;33(12).
336 Su Shengyong, Deng Baiying, Li Yangfan, Zhou Enhua. Recent curative effect of acupuncture and moxibustion against hemiplegic shoulder pain after stroke. Chinese acupuncture. 2004;24(12).
337 Luo Ying, Xiao Xue, Yang Liye. The curative effect of qihuang acupuncture in treating shoulder pain after stroke. Reflexology and rehabilitation medicine. 2021;2(16):21-3.
338 Xiong Xianting, Ma Han Yi, Xu Linling, Chen Chaoming. Clinical observation of manual rehabilitation combined with electroacupuncture in the treatment of complex regional pain syndrome after stroke. Chinese medical innovation. 2023;20(6).
339 Zhang Yanhui, Wang Peng, very beautiful flowers. Clinical observation of acupuncture exercise therapy for hemiplegic shoulder pain after stroke. Chinese medical innovation. 2022;19(34).
340 Du Yuanhao, Zhang Man, Yin Xiumei, Di Jiawei, Ye Yanzhen. Meta-analysis and GRADE evidence rating of acupuncture for shoulder hand syndrome after stroke. Chinese folk therapy. 2023;31(5).
341 Wei Zemin, Sima Xunxin, Wu Qiang, Gong Jianqiu. To systematically evaluate the clinical efficacy of floating needle therapy for poststroke shoulder pain. New traditional Chinese medicine. 2019(10).
342 Qiu Zhiqin, Yu Mengting, Li Zhuangmiao, Yan Wenjuan, Zhang Jiayu,. Meta-analysis of wrist and ankle injection for hemiplegic shoulder pain in stroke. TCM clinical research. 2022;14(15).
343 Zhan J, Wei X, Tao C, Yan X, Zhang P, Chen R, et al. Effectiveness of acupuncture combined with rehabilitation training vs. rehabilitation training alone for post-stroke shoulder pain: A systematic review and meta-analysis of randomized controlled trials. Frontiers in medicine. 2022;9:947285.
344 Zhan J, Luo Y, Mao W, Zhu L, Xu F, Wang Y, et al. Efficacy of acupuncture versus rehabilitation therapy on post-stroke shoulder pain: A systematic review and meta-analysis of randomized controlled trials. Medicine. 2023;102(29):e34266.
345 Lee SH, Lim SM. Acupuncture for Poststroke Shoulder Pain: A Systematic Review and Meta-Analysis. Evidence-based complementary and alternative medicine : eCAM. 2016;2016:3549878.
346 Chau JPC, Lo SHS, Yu X, Choi KC, Lau AYL, Wu JCY, et al. Effects of Acupuncture on the Recovery Outcomes of Stroke Survivors with Shoulder Pain: A Systematic Review. Frontiers in neurology. 2018;9:30.

Appendix 4.The regional distribution of literature was included.

Country	City	count	proportion
China	Guangdong	47	10.1%
	Shangdong	22	6.4%
	Guangxi	20	5.8%
	Jiangsu	28	5.8%
	Zhejiang	19	5.5%
	Anhui	18	5.2%
	Fujian	17	4.9%
	Heilongjiang	17	4.9%
	Tianjin	20	4.9%
	Beijing	19	4.0%
	Henan	14	4.0%
	Sichuan	12	3.5%
	Hubei	9	2.6%
	Hunan	9	2.6%
	Shanxi	9	2.6%
	Jilin	8	2.3%
	Liaoning	8	2.3%
	Jiangxi	7	2.0%
	Hebei	5	1.4%
	Shangxi	5	1.2%
	Xinjiang	3	0.9%
	Chongqing	8	0.9%
	Gansu	2	0.6%
	Guizhou	2	0.6%
	Shanghai	7	0.6%
	Hainan	1	0.3%
	Inner Mongolia	1	0.3%
	Ningxia	1	0.3%
	Qinghai	1	0.3%
	Yunnan	1	0.3%
	Hongkong	1	0.3%
Iran	Tabriz	1	0.3%
South Korea	Seoul	4	1.2%

Appendix 5.Detailed table of Intervention and control design characteristics of RCTs.

Ref. No.	Group of RCTs	Basic treatment	Type of Intervention	Acupuncture method	EA waveform	Frequency of EA	Sham Acupuncture Setting	Type of Control	Course of intervention
1	3	N	Acup	Floating needle	/	/	/	Acup	4w
			Acup	Floating needle	/	/	/	RT	/
			Acup	Body acupuncture	/	/	/	RT	/
2	2	N	Acup+RT	EA	Not reported	100Hz	/	RT+Other	5w
3	2	Y	Acup+RT	Body acupuncture	/	/	Tactile stimulation, non-penetrating, retractable needles were used	Sham-acup+RT	3w
4	2	N	M-Acup+RT	Body acupuncture+Moxibustion	/	/	/	RT	12w
5	2	Y	M-Acup+RT	Body acupuncture+Scalp acupuncture	/	/	/	RT	4w
6	2	N	Acup	Acupoint injection	/	/	Acupoint injection of saline solution	Sham-acup	4w
7	2	Y	Acup+RT	EA	Not reported	2/100Hz	Sham acupuncture applied to non-meridian and non-acupoint locations	Sham-acup+RT	2w
8	2	N	Acup	Warm acupuncture	/	/	/	RT	2w
9	2	Y	M-Acup+RT	Body acupuncture+Moxibustion + Scalp acupuncture	/	/	/	M-Acup+RT	4w
10	2	N	Acup+CM	Body acupuncture	Dense wave	The patient's tolerance was taken as the degree.	/	CM	4w
11	2	Y	Acup	Body acupuncture	/	/	/	Blank	4w
12	2	N	Acup+Other	Body acupuncture	/	/	/	Other	4w
13	2	N	Acup	Body acupuncture	/	/	/	CM	4w
14	2	Y	Acup	Body acupuncture	/	/	/	CM	4w
15	2	Y	Acup	Body acupuncture	/	/	/	CM	4w
16	2	Y	M-Acup	Body acupuncture+Body acupuncture	/	/	/	Acup+CM	4w
17	2	Y	Acup+RT	EA	Sparse wave	2Hz	/	RT	12w
18	2	Y	Acup	Elongated needle	/	/	/	Acup	20d
19	2	N	Acup+RT	EA	Dispersive	9~12Hz	/	RT	Not

					dense wave				reported
20	2	Y	M-Acup+RT	Body acupuncture+Moxibustion	/	/	/	RT	30d
21	2	N	Acup+RT	Warm acupuncture	/	/	/	Acup+RT	Not reported
22	2	Y	M-Acup+RT	Catgut embedding therapy+Body acupuncture	/	/	/	Acup+RT	12w
23	2	Y	Acup	Body acupuncture	/	/	/	Acup	2w
24	2	Y	Acup	Body acupuncture	/	0	/	Acup	4w
25	2	Y	Acup+RT	Acupoint Application	/	/	/	RT	4w
26	2	N	Acup+RT	Body acupuncture	/	/	/	RT+CM	1m
27	3	Y	Acup+RT	EA	Sparse wave	2Hz	/	RT	32d
27			Acup	EA	/	/	/	RT	/
28	3	Y	Acup+RT	EA	Sparse wave	2Hz	/	RT	32d
28			Acup	EA	/	/	/	RT	/
29	2	N	M-Acup	Body acupuncture+Scalp acupuncture	Continuous wave	Low frequency	/	M-Acup	4w
30	2	N	Acup+RT	Body acupuncture	/	/	/	RT+CM	2w
31	2	N	Acup	Body acupuncture	/	/	/	Acup	3w
32	2	N	Acup+RT	Body acupuncture	/	/	/	RT	30d
33	2	N	Acup	Body acupuncture	/	/	/	Acup	Not reported
34	2	N	M-Acup	Three-edged needle+Body acupuncture	/	/	/	Other	4w
35	3	N	Acup+RT	Moxibustion	/	/	/	RT+Other	4w
35			FAcup+RT	Moxibustion	/	/	/	RT	/
36	2	Y	Acup+RT	Floating needle	/	/	/	RT	8w
37	3	N	Acup+RT	Intradermal needle	/	/	/	RT	10w
37			FAcup+RT+Other	Intradermal needle	/	/	/	RT+Other	/
38	3	N	Acup+RT	Body acupuncture	/	/	/	RT	1m
38			Acup	Body acupuncture	/	/	/	RT	/
39	3	Y	Acup+RT+Other	Acupoint injection	/	/	/	RT+Other	/
39			Acup+RT	Acupoint injection	/	/	/	RT+Other	/
40	3	Y	Acup+RT+Other	Acupoint injection	/	/	/	RT+Other	/
40			Acup+RT	Acupoint injection	/	/	/	RT+Other	/

41	2	N	Acup+RT	Intradermal needle	/	/	/	RT	/
42	2	N	Acup	Body acupuncture	/	/	/	Acup	3w
43	2	Y	Acup+RT	Body acupuncture	/	/	/	Acup+RT	4w
44	2	Y	Acup+RT	EA	Dispersive dense wave	2/100Hz	/	RT	4w
45	2	Y	Acup	Body acupuncture	/	/	/	Acup	2w
46	2	Y	Acup	Body acupuncture	/	/	/	Acup	8w
47	2	Y	Acup+RT	Floating needle	/	/	/	RT	60d
48	2	N	Acup	EA	Continuous wave	2～4V	/	Acup	4w
49	3	N	Acup+RT	Body acupuncture	/	/	/	RT	28d
49			Acup	Body acupuncture	/	/	/	RT	/
50	2	Y	Acup+RT	Warm acupuncture	/	/	/	Acup+RT	2w
51	2	Y	Acup+CM	Acupoint injection	/	/	/	CM	14d
52	2	Y	M-Acup+RT	Ear acupuncture+ Balance acupuncture	/	/	/	Acup+RT	4w
53	2	Y	M-Acup	Body acupuncture+Acupotomy	/	/	/	Acup	2w
54	2	N	Acup+Other	EA	Dispersive dense wave	2Hz、10 Hz、100Hz	/	Other	4w
55	2	Y	Acup+RT	Wrist-ankle needle	/	/	/	RT	4w
56	2	N	M-Acup	Scalp acupuncture+Body acupuncture	/	/	/	Acup	2w
57	2	Y	Acup+RT	Body acupuncture	/	/	/	RT	3w
58	2	Y	Acup+RT	Acupoint injection	/	/	/	RT	4w
59	2	N	M-Acup+RT	Body acupuncture+Wrist-ankle needle	/	/	/	RT	Not reported
60	3	N	M-Acup+CM	Body acupuncture+Scalp acupuncture	/	/	/	CM	4w
60			M-Acup	Body acupuncture+Scalp acupuncture	/	/	/	CM	/
61	2	N	Acup+RT	Scalp acupuncture	/	/	/	RT	3w
62	2	N	Acup+RT	Body acupuncture	/	/	/	RT	6w
63	2	N	Acup+RT	Ear acupuncture	/	/	/	RT	1m
64	2	N	Acup+RT	Body acupuncture	/	/	/	RT	4w
65	3	N	M-Acup+RT	Body acupuncture + Scalp acupuncture+Intradermal needle	/	/	/	M-Acup+RT	4w
65			M-Acup+RT	Body acupuncture + Scalp acupuncture+Intradermal needle	/	/	/	RT	/

65			M-Acup+RT	Scalp acupuncture+Body acupuncture	/	/	/	RT	/
66	3	Y	Acup+TCM	Body acupuncture	/	/	/	TCM	4w
66			Acup	Body acupuncture	/	/	/	TCM	/
67	3	Y	Acup+TCM	Body acupuncture	/	/	/	TCM	4w
67			M-Acup	Body acupuncture	/	/	/	TCM	/
68	2	Y	Acup+RT+Other	Intradermal needle	/	/	/	RT+Other	4w
69	2	N	Acup+RT	Warm acupuncture	/	/	/	RT	4w
70	2	N	M-Acup+RT	Body acupuncture+Elongated needle	/	/	/	Acup+RT	12w
71	2	N	Acup+RT	Body acupuncture	/	/	/	RT	1m
72	2	Y	Acup	Body acupuncture	/	/	/	Acup	4w
73	2	N	Acup+RT	Acupoint Application	/	/	/	RT	4w
74	2	N	Acup	Warm acupuncture	/	/	/	Acup	4w
75	2	N	Acup+RT	Warm acupuncture	/	/	/	RT	4w
76	2	N	M-Acup	Body acupuncture+Moxibustion	/	/	/	Acup	14d
77	2	Y	M-Acup+RT	Body acupuncture+Acupoint injection	/	/	/	Acup+RT	34d
78	2	Y	Acup	Body acupuncture	/	/	/	Blank	10d
79	2	Y	M-Acup	Body acupuncture+Acupotomy	/	/	/	Acup	2w
80	2	N	Acup	Warm acupuncture	/	/	/	RT	30d
81	2	N	Acup+RT	Abdominal acupuncture	/	/	/	RT	4w
82	2	Y	Acup	Body acupuncture	/	/	/	Acup	4w
83	2	Y	M-Acup	Three-edged needle+Body acupuncture	/	/	/	Acup	3w
84	3	N	M-Acup	Body acupuncture+Scalp acupuncture	/	/	/	Acup	Not reported
84			Acup	Body acupuncture	/	/	/	Acup	/
85	2	N	Acup+Other	Body acupuncture	/	/	/	Other	4w
86	2	N	M-Acup	Body acupuncture+Scalp acupuncture	/	/	/	CM	4w
87	2	Y	M-Acup	Body acupuncture+Scalp acupuncture+Wrist-ankle needle	/	/	/	Acup	4w
88	2	N	Acup	Acupotomy	/	/	/	Acup	4w
89	2	N	Acup	EA	Continuous wave	0 ~ 10 Hz	/	Acup	4w
90	3	Y	M-Acup	Moxibustion+Body acupuncture	/	/	/	M-Acup	30d
90			M-Acup	Moxibustion+Body acupuncture	/	/	/	RT	/
90			M-Acup	Moxibustion+Body acupuncture	/	/	/	RT	/

91	2	N	M-Acup	Body acupuncture+Three-edged needle	/	/	/	CM	6w
92	2	N	M-Acup	Body acupuncture+Abdominal acupuncture	/	/	/	Acup	Not reported
93	2	Y	Acup	Body acupuncture	/	/	/	Acup	2w
94	2	N	Acup+RT	Body acupuncture	/	/	/	RT	1m
95	2	N	Acup+CM	Body acupuncture	/	/	/	CM	8w
96	2	N	M-Acup	Body acupuncture+Wrist-ankle needle	/	/	/	Acup	14d
97	3	Y	Acup+RT	Floating needle	/	/	/	RT	4w
97			Acup	Floating needle	/	/	/	RT	/
98	2	N	Acup	Body acupuncture	/	/	/	Acup	4w
99	3	N	Acup+Other	Body acupuncture	/	/	/	Other	4w
99			Acup	Body acupuncture	/	/	/	TCM	/
100	2	Y	M-Acup	Moxibustion+Three-edged needle	/	/	/	RT	4w
101	2	N	Acup+RT	Body acupuncture	/	/	/	RT	8w
102	2	N	Acup	Warm acupuncture	/	/	/	Acup	4w
103	2	Y	Acup+RT	Body acupuncture	/	/	/	RT	4w
104	2	N	Acup+CM	Body acupuncture	/	/	/	CM	4w
105	2	Y	M-Acup	Body acupuncture+Three-edged needle	/	/	/	Acup	2w
106	3	N	Acup+Other	Body acupuncture	/	/	/	Other	4w
106			Acup	Body acupuncture	/	/	/	Other	/
107	2	N	Acup+RT	Body acupuncture	/	/	/	RT	Not reported
108	2	N	Acup+RT	Body acupuncture	/	/	/	RT	4w
109	3	Y	Acup+Other	EA	Dense wave	50Hz	/	Other	2w
109			Acup	EA	/	/	/	Other	/
110	2	Y	Acup+RT	Body acupuncture	/	/	/	RT	4w
111	2	Y	Acup	Body acupuncture	/	/	/	Blank	28d
112	2	Y	Acup	Body acupuncture	/	/	/	Blank	42d
113	2	N	Acup	Body acupuncture	/	/	/	Acup	30d
114	3	Y	Acup+RT	Body acupuncture	/	/	/	RT	4w
114			Acup	Body acupuncture	/	/	/	RT	/
115	2	Y	Acup+Other	Body acupuncture	/	/	/	Other	14d
116	2	N	Acup	Elongated needle	/	/	/	Acup	2w
117	2	N	Acup	EA	Dense wave	100times/s	/	CM	30d

118	2	N	Acup+RT	Body acupuncture	/	/	/	RT	28d
119	3	N	Acup+RT	EA	Continuous wave/Dispersive dense wave	Continuous wave:15Hz; Dispersive dense wave:2Hz/100Hz	/	Acup+RT	4w
119			Acup+RT	EA	/	/	/	Acup+RT	/
119			Acup+RT	EA	/	/	/	Acup+RT	/
120	2	Y	Acup	Body acupuncture	/	/	/	Blank	6w
121	2	N	Acup	EA	Continuous wave	2Hz	/	Acup	2w
122	3	Y	Acup	Warm acupuncture	/	/	/	Acup	2w
122			Acup	Warm acupuncture	/	/	/	Acup	/
122			Acup	Warm acupuncture	/	/	/	Acup	/
123	3	Y	Acup+RT	Body acupuncture	/	/	/	RT	4w
123			Acup	Body acupuncture	/	/	/	RT	/
124	3	N	Acup+TCM	Body acupuncture	/	/	/	TCM	4w
124			Acup	Body acupuncture	/	/	/	TCM	/
125	2	N	Acup+RT	EA	Dispersive dense wave	1~20Hz, 6V	/	RT	2w
126	2	Y	Acup	Body acupuncture	/	/	/	Acup	4w
127	3	Y	Acup	Body acupuncture	/	/	/	Blank	2w
128	2	Y	Acup	EA	Dispersive dense wave	The patient's tolerance was taken as the degree.	/	Acup	2w
129	3	Y	Acup+RT+Other	Body acupuncture	/	/	/	RT+Other	4w
129			Acup+RT	Body acupuncture	/	/	/	RT+Other	/
130	2	N	Acup+RT	Wrist-ankle needle	/	/	/	RT	23d
131	3	N	M-Acup+RT	Scalp acupuncture+Body acupuncture	/	/	/	RT	1m
131			M-Acup	Scalp acupuncture+Body acupuncture	/	/	/	PNF	/
132	3	N	M-Acup	Body acupuncture+Scalp acupuncture	/	/	/	Acup	Not reported
132			Acup	Body acupuncture	/	/	/	Acup	/
133	2	N	Acup	Body acupuncture	/	/	/	Acup	20d
134	2	Y	Acup+RT	EA	/	/	/	RT	4w

135	2	N	Acup+RT	Warm acupuncture	/	/	/	Acup+RT	4w
136	2	Y	Acup	Elongated needle	/	/	/	CM	4w
137	3	Y	Acup+RT	EA	Dispersive dense wave	8~10 Hz	/	RT	3w
137			Acup	EA	/	/	/	RT	/
138	2	N	Acup	Floating needle	/	/	/	Acup	10d
139	2	Y	Acup+TCM+RT	Body acupuncture	/	/	/	Acup+TCM+RT	20d
140	2	N	Acup	Body acupuncture	/	/	/	Acup	10d
141	2	Y	Acup+RT	Body acupuncture	/	/	/	RT	4w
142	2	N	Acup	EA	Dense wave	Not reported	/	CM	30d
143	4	Y	Acup	EA	Dispersive dense wave	High frequency:100Hz; Low frequency:2Hz	/	Acup	14d
144	2	Y	Acup	Body acupuncture	/	/	/	CM	2w
145	2	N	Acup	Body acupuncture	/	/	/	CM	3w
146	2	N	Acup+RT	Scalp acupuncture	/	/	/	Acup+RT	3m
147	2	N	Acup+RT	Floating needle	/	/	/	RT	2w
148	3	Y	Acup+RT	EA	Intermittent waves	/	/	RT	4w
148			Acup	EA	/	/	/	RT	/
149	2	N	Acup+RT	Body acupuncture	/	/	/	RT	14d
150	2	N	Acup	Body acupuncture	/	/	/	Acup	30d
151	2	N	Acup	EA	Dense wave	Not reported	/	CM	30d
152	2	Y	M-Acup	Body acupuncture+Scalp acupuncture+Wrist-ankle needle	/	/	/	Acup	4w
153	3	Y	Acup+TCM	Body acupuncture	/	/	/	TCM	4w
153			Acup	Body acupuncture	/	/	/	TCM	/
154	2	Y	M-Acup	Body acupuncture+Moxibustion	/	/	/	TCM	4w
155	2	Y	Acup+RT	Body acupuncture	/	/	/	RT	2m
156	2	N	Acup+RT	Warm acupuncture	/	/	/	RT	4w
157	2	Y	Acup+RT	Warm acupuncture	/	/	/	RT	15d
158	2	Y	Acup+RT	Warm acupuncture	/	/	/	RT	7d
159	2	N	Acup+RT	Body acupuncture	/	/	/	RT	8w
160	2	Y	Acup+Other	EA	Continuous	0~10 Hz	/	Acup+Other	6w

					wave				
161	2	Y	Acup+RT	Body acupuncture	/	/	/	Acup+RT	3w
162	2	Y	Acup+RT	Body acupuncture	/	/	/	RT	2w
163	2	Y	Acup	Warm acupuncture	/	/	/	Blank	4w
164	2	Y	Acup	Body acupuncture	/	/	/	CM	4w
165	2	Y	Acup+RT	Body acupuncture	/	/	/	Acup+RT	4w
166	2	N	Acup+RT	Balance acupuncture	/	/	/	RT	4w
167	2	N	Acup+RT	EA	Continuous wave	Not reported	/	Acup+RT	30d
168	2	Y	Acup+Other	Hooked needle	/	/	/	Acup+Other	2w
169	2	Y	M-Acup	Body acupuncture+Moxibustion	/	/	/	Acup	3w
170	2	Y	M-Acup	Body acupuncture+Moxibustion	/	/	/	Acup	3w
171	2	N	Acup+RT	Body acupuncture	/	/	/	RT+Other	8w
172	2	Y	Acup+RT	EA	Continuous wave	2Hz	/	Acup+RT	3w
173	2	Y	Acup+RT	EA	Dispersive dense wave	9~12Hz	/	RT	4w
174	2	Y	M-Acup	Body acupuncture+Moxibustion	/	/	/	RT	28d
175	2	Y	Acup	Warm acupuncture	/	/	/	Acup	15d
176	2	Y	Acup+RT	Body acupuncture	/	/	/	RT	2m
177	2	N	M-Acup+RT	Body acupuncture+Scalp acupuncture	/	/	/	RT	20d
178	2	Y	Acup	EA	Dense wave	50-100times/s	/	Acup	2w
179	2	N	Acup+RT	Abdominal acupuncture	/	/	/	RT	3w
180	2	Y	Acup	Acupotomy	/	/	/	CM	4w
181	2	N	Acup	Balance acupuncture	/	/	/	Acup	10d
182	3	Y	Acup+RT	Moxibustion	/	/	/	RT	4w
182			Acup	Moxibustion	/	/	/	RT	/
183	2	Y	M-Acup	Body acupuncture+Scalp acupuncture+Moxibustion	/	/	/	Blank	Not reported
184	2	Y	Acup	TAES	/	/	/	Blank	2w
185	2	N	Acup+RT+Other	Body acupuncture	/	/	/	RT+Other	1m
186	3	Y	Acup	Scalp acupuncture	/	/	/	Acup	2w
186			Acup	Body acupuncture	/	/	/	CM	/
186			Acup	Scalp acupuncture	/	/	/	CM	/

187	3	N	Acup+RT	EA	Continuous wave	2Hz	/	RT	4w
187			Acup	EA	/	/	/	RT	/
188	2	N	Acup+RT	Warm acupuncture	/	/	/	RT	20d
189	3	N	Acup+RT	Floating needle	/	/	/	TCM+RT	1m
189			Acup+RT	Floating needle	/	/	/	RT+CM	/
190	2	Y	Acup	Moxibustion	/	/	/	RT	3m
191	2	N	Acup+RT	Wrist-ankle needle	/	/	/	RT	3w
192	2	N	M-Acup	EA+Scalp acupuncture	Dispersive dense wave	1Hz	/	Acup	4w
193	2	N	Acup+RT	Moxibustion	/	/	/	RT	20d
194	2	N	Acup+RT	Floating needle	/	/	/	RT	6w
195	2	Y	Acup+RT	Moxibustion	/	/	/	RT	4w
196	2	Y	Acup	EA	Dispersive dense wave	2/100Hz	No power on	Sham-acup	7d
197	2	N	Acup+RT	Moxibustion	/	/	/	RT	6w
198	2	Y	Acup	Body acupuncture	/	/	/	Acup	2w
199	2	Y	Acup+RT+Other	Floating needle	/	/	/	RT+Other	4w
200	2	Y	Acup	EA	Dispersive dense wave	Not reported	/	CM	23d
201	2	Y	Acup	Body acupuncture	/	/	/	Blank	30d
202	2	Y	Acup	Body acupuncture	/	/	/	Acup	4w
203	2	Y	Acup	Body acupuncture	/	/	/	RT	2w
204	2	N	M-Acup	Skin needle+Three-edged needle	/	/	/	CM	2w
205	3	Y	Acup+RT+Other	Acupoint injection	/	/	/	RT+Other	/
205			Acup+RT	Acupoint injection	/	/	/	RT+Other	/
206	2	N	Acup	Body acupuncture	/	/	/	CM	20d
207	2	Y	Acup+RT	Floating needle	/	/	/	RT	2w
208	2	Y	Acup	Body acupuncture	/	/	/	Acup	3w
209	2	Y	Acup+RT	Body acupuncture	/	/	/	RT	3w
210	2	N	Acup+RT+Other	Body acupuncture	/	/	/	RT+Other	1m
211	2	Y	Acup	Body acupuncture	/	/	/	Acup	3w
212	2	Y	Acup+RT	Balance acupuncture	/	/	/	RT+CM	2w
213	2	Y	Acup	Acupoint Application	/	/	/	Blank	Not

									reported
214	2	Y	Acup+RT	Floating needle	/	/	/	RT	2w
215	2	Y	Acup	Body acupuncture	/	/	/	CM	2w
216	2	Y	Acup+RT	Body acupuncture	/	/	/	RT	4w
217	2	Y	M-Acup	EA+Scalp acupuncture	/	/	/	CM	60d
218	2	Y	Acup	Body acupuncture	/	/	/	Acup	4w
219	2	Y	M-Acup+RT	Fire needle+Body acupuncture	/	/	/	RT	3w
220	2	Y	Acup	EA	Dispersive dense wave	/	/	RT	23d
221	2	N	Acup+RT	Warm acupuncture	/	/	/	Acup+RT	2w
222	3	Y	Acup+RT	Body acupuncture	/	/	/	RT	20d
222			Acup+RT	Body acupuncture	/	/	/	RT	/
223	3	Y	Acup+RT	Abdominal acupuncture	/	/	/	RT	4w
223			AAcup	Abdominal acupuncture	/	/	/	RT	/
224	2	Y	Acup+RT	Body acupuncture	/	/	/	RT	4w
225	3	Y	Acup+RT	Floating needle	/	/	/	RT	4w
225			FAcup+RT+Other	Floating needle	/	/	/	RT+Other	/
226	4	N	Acup+Other	Eyes acupuncture	/	/	/	Other	6w
226			GAcup+Other疗法	Eyes acupuncture	/	/	/	Other	/
226			GAcup+Other疗法	Eyes acupuncture	/	/	/	Other	/
227	2	Y	Acup+RT	Abdominal acupuncture	/	/	/	RT	4w
228	2	N	M-Acup	Intradermal needle+ Balance acupuncture	/	/	/	Acup	90d
229	2	Y	M-Acup+RT	Body acupuncture+Scalp acupuncture+Wrist-ankle needle	/	/	/	RT	4w
230	2	Y	Acup+TCM	Body acupuncture	/	/	/	TCM	4w
231	2	Y	Acup	Body acupuncture	/	/	/	Blank	42d
232	2	N	Acup+RT	Umbilical needle	/	/	/	Acup+RT	2w
233	2	Y	M-Acup	Scalp acupuncture+Body acupuncture	/	/	/	M-Acup	4w
234	2	Y	M-Acup	EA+Scalp acupuncture	Continuous wave/Dense wave	100Hz	/	Acup	4w

235	2	N	Acup+RT	Body acupuncture	/	/	/	RT	2m
236	2	N	Acup	EA	Continuous wave	50Hz	/	Acup	4w
237	2	N	Acup+RT	Body acupuncture	/	/	/	RT	4w
238	2	N	Acup+RT	Body acupuncture	/	/	/	RT	20d
239	3	Y	M-Acup+TCM	Body acupuncture+Moxibustion	/	/	/	TCM	4w
239			M-Acup	Body acupuncture+Moxibustion	/	/	/	TCM	/
240	2	Y	Acup	Body acupuncture	/	/	/	RT	2w
241	2	Y	Acup	Body acupuncture	/	/	/	Acup	1w
242	2	N	Acup+RT	EA	Not reported	/	/	RT	30d
243	2	N	Acup	Wrist-ankle needle	/	/	/	CM	Not reported
244	2	N	Acup	Body acupuncture	/	/	/	CM	4w
245	3	N	Acup+RT	Body acupuncture	/	/	/	RT	2w
245			Acup	Body acupuncture	/	/	/	RT	/
246	3	N	Acup+RT	Body acupuncture	/	/	/	RT	Not reported
246			Acup	Body acupuncture	/	/	/	RT	/
247	2	N	Acup+RT	Wrist-ankle needle	/	/	/	RT	Not reported
248	2	Y	Acup	Body acupuncture	/	/	/	Blank	3w
249	2	N	Acup+RT	EA	Continuous wave	/	/	RT	Not reported
250	2	Y	Acup+RT	Warm acupuncture	/	/	/	Acup+RT	2w
251	2	N	Acup+RT	Warm acupuncture	/	/	/	Acup+RT	4w
252	3	Y	M-Acup	Body acupuncture+Catgut embedding therapy	/	/	/	Blank	4w
252			Acup	Body acupuncture	/	/	/	Blank	/
253	3	Y	M-Acup+RT+Other	Body acupuncture+ Balance acupuncture	/	/	/	RT+Other	8w
253			M-Acup+RT		/	/	/	RT+Other	/
254	2	Y	Acup	Body acupuncture	/	/	/	Acup	4w
255	2	N	Acup	EA	Continuous wave	5Hz	/	CM	10d

256	2	Y	Acup	Body acupuncture	/	/	/	Acup	12w
257	3	Y	Acup+RT	Abdominal acupuncture	/	/	/	RT	4w
257			Acup	Abdominal acupuncture	/	/	/	RT	/
258	2	N	Acup	Three-edged needle	/	/	/	Acup	4w
259	2	Y	Acup+RT	EA	Not reported	2Hz/15 Hz	/	RT	4w
260	2	Y	M-Acup	Scalp acupuncture+EA	Dispersive dense wave	1Hz	/	CM	4w
261	2	Y	Acup	Catgut embedding therapy	/	/	/	Acup	1m
262	2	N	Acup+Other	Floating needle	/	/	/	Other	4w
263	2	N	Acup	Body acupuncture	/	/	/	Other	6w
264	2	Y	Acup+RT	Three-edged needle	/	/	/	Acup+RT	19d
265	2	Y	Acup+RT	Body acupuncture	/	/	/	Acup+RT	3w
266	2	Y	M-Acup	Moxibustion+Body acupuncture	/	/	/	RT	4w
267	2	N	Acup	Body acupuncture	/	5未提及	/	Acup	4w
268	2	N	M-Acup+RT	Body acupuncture+Scalp acupuncture	/	/	/	M-Acup+RT	6w
269	2	N	Acup+TCM	Skin needle	/	/	/	TCM	Not reported
270	3	N	Acup+Other	Body acupuncture	/	/	/	Other	30d
270			Acup+Other	Body acupuncture	/	/	/	Other	/
271	2	Y	Acup+RT	Abdominal acupuncture	/	/	/	RT	2w
272	2	Y	M-Acup	Body acupuncture+Catgut embedding therapy	/	/	/	Acup	4w
273	2	N	M-Acup	Body acupuncture+Moxibustion	/	/	/	RT	30d
274	3	Y	Acup+Other	Floating needle	/	/	/	RT+Other	2w
274			Acup+RT	Floating needle	/	/	/	RT+Other	/
275	2	Y	Acup	Body acupuncture	/	/	/	Blank	4w
276	3	Y	Acup+TCM	Body acupuncture	/	/	/	TCM	36d
276			Acup	Body acupuncture	/	/	/	TCM	/
277	3	N	Acup+Other	Body acupuncture	/	/	/	Other	6w
277			Acup	Body acupuncture	/	/	/	Other	/
278	2	N	Acup+RT	Warm acupuncture	/	/	/	Acup+RT	2w
279	2	N	Acup	Body acupuncture	/	/	/	Acup	4w
280	2	Y	M-Acup	Body acupuncture+Scalp acupuncture	/	/	/	Acup	4w
281	3	Y	Acup+RT	EA	Sparse wave	2Hz	/	RT	20d

			Acup	EA	/	/	/	RT	/
282	2	N	Acup+RT	Scalp acupuncture	/	/	/	Acup+RT	3m
283	2	Y	Acup	EA	Dispersive dense wave	Sparse wave:2Hz; Dense wave:15Hz	/	RT	4w
284	2	Y	Acup+RT	Body acupuncture	/	/	/	Acup+RT	14d
285	2	Y	Acup+RT	Body acupuncture	/	/	/	Acup+RT	3w
286	2	Y	Acup+RT	Fire needle	/	/	/	RT	13d
287	2	Y	Acup	Body acupuncture	/	/	/	TCM	4w
288	2	Y	Acup+RT	Warm acupuncture	/	/	/	RT	3w
289	2	N	Acup+RT	Wrist-ankle needle	/	/	/	Acup+RT	30d
290	2	N	Acup+RT	Warm acupuncture	/	/	/	Acup+RT	4w
291	2	Y	Acup+RT	EA	Dispersive dense wave	0~5V	/	RT	4w
292	2	N	M-Acup+CM	Body acupuncture+Scalp acupuncture+Wrist-ankle needle	/	/	/	CM+Other	8w
293	2	Y	Acup	Body acupuncture	/	/	/	RT	2w
294	2	Y	Acup+RT	Acupoint injection	/	/	/	RT	30d
295	2	Y	M-Acup+Other	Intradermal needle+Body acupuncture	/	/	/	Acup+Other	8w
296	3	Y	Acup+RT	Intradermal needle	/	/	/	RT	9w
			Acup+RT	Intradermal needle	/	/	/	RT	/
297	2	Y	Acup+RT	Body acupuncture	/	/	/	RT	4w
298	2	Y	Acup+Other	Moxibustion	/	/	/	Other	30d
299	2	N	Acup	Body acupuncture	/	/	/	Acup	Not reported
300	2	Y	Acup+RT	Abdominal acupuncture	/	/	/	RT	2w
301	2	N	Acup	Body acupuncture	/	/	/	Acup	Not reported
302	2	Y	Acup+RT	Warm acupuncture	/	/	/	RT	3w
303	2	N	Acup+RT	Body acupuncture	/	/	/	RT	4w
304	2	Y	Acup+RT	Acupotomy	/	/	/	Acup+RT	4w
305	2	Y	Acup	Wrist-ankle needle	/	/	/	Acup	4w
306	2	Y	Acup	Wrist-ankle needle	/	/	/	Acup	4w
307	2	N	Acup	Body acupuncture	/	/	/	Acup	4w
308	2	Y	Acup	Acupoint injection	/	/	/	CM	Not

									reported
309	2	N	Acup+RT	Floating needle	/	/	/	Acup+RT	4w
310	2	Y	Acup	EA	Sparse wave	/	/	Acup	10d
311	2	Y	Acup+RT	Body acupuncture	/	/	/	RT	3w
312	2	Y	Acup+RT	EA	Sparse wave	2 Hz	/	RT+Other	Not reported
313	2	Y	Acup+RT	EA	Continuous wave	2～5 Hz	/	RT+CM	2w
314	2	Y	Acup	Body acupuncture	/	/	/	Acup	4w
315	2	Y	Acup	Body acupuncture	/	/	/	Acup	4w
316	2	Y	M-Acup+RT	EA+Scalp acupuncture	Not reported	/	/	RT	4w
317	3	N	Acup+RT	Wrist-ankle needle	/	/	/	Acup+RT	22d
317			Acup+RT	Wrist-ankle needle	/	/	/	Acup+RT	/
317			Acup+RT	Wrist-ankle needle	/	/	/	Acup+RT	/
318	3	Y	Acup+RT	Body acupuncture	/	/	/	RT	6w
			Acup	Body acupuncture	/	/	/	RT	/
319	2	Y	Acup+RT	Body acupuncture	/	/	/	RT+CM	14d
320	2	Y	M-Acup	Body acupuncture+Scalp acupuncture	/	/	/	M-Acup	4w
321	2	N	Acup+RT	Warm acupuncture	/	/	/	RT	4w
322	2	N	Acup+TCM	Warm acupuncture	/	/	/	TCM	4w
323	3	N	Acup+TCM	Body acupuncture	/	/	/	TCM	4w
			Acup	Body acupuncture	/	/	/	TCM	/
324	2	N	Acup+RT+Other	Body acupuncture	/	/	/	RT+Other	4w
325	2	N	Acup+RT	Body acupuncture	/	/	/	RT	28d
326	2	N	Acup	Wrist-ankle needle	/	/	/	Acup	10d
327	2	N	Acup+RT	Body acupuncture	/	/	/	RT	6w
328	2	Y	Acup+RT	Moxibustion	/	/	/	RT	Not reported
329	2	Y	Acup	Balance acupuncture	/	/	/	RT	3w
330	2	Y	Acup+RT	Moxibustion	/	/	/	RT	4w
331	2	Y	M-Acup+RT	Scalp acupuncture+EA	Dispersive dense wave	Not reported	/	M-Acup+RT	4w
332	2	Y	Acup	Ear acupuncture	/	/	/	Blank	30d
333	2	Y	Acup	Body acupuncture	/	/	/	RT	3w

334	2	N	Acup+RT	Body acupuncture	/	/	/	RT	4w
335	2	Y	Acup+RT	Moxibustion	/	/	/	RT	Not reported
336	3	Y	Acup+TCM	Body acupuncture	/	/	/	TCM	4w
			Acup	Body acupuncture	/	/	/	TCM	/
337	2	N	Acup	Body acupuncture	/	/	/	Acup	2w
338	2	Y	Acup+RT	EA	Continuous wave	120~250times/min	/	RT	3w
347	2	N	Acup	Body acupuncture	/	/	/	Acup	3w

Note: Specific references are given at the end of Appendix 3.

Appendix 6.The intervention and control design of the included RCTs.

Objectives of the Study	Intervention group VS control group	count (n=402)	proportion
Comparison of different acupuncture factors	Acup VS Acup	61	15.17%
	Acup+RT VS Acup+RT	29	7.21%
	M-Acup VS Acup	19	4.73%
	M-Acup+RT VS M-Acup+RT	4	1.00%
	M-Acup VS M-Acup	4	1.00%
	Acup+Other VS Acup+Other	2	0.50%
	Acup+TCM+RT VS Acup+TCM+RT	1	0.25%
Comparison of synergistic effects of acupuncture and other therapies	Acup+RT VS RT	104	25.87%
	Acup+Other VS Other	15	3.73%
	Acup+RT+Other VS RT+Other	11	2.74%
	M-Acup+RT VS RT	11	2.74%
	Acup+TCM VS TCM	10	2.49%
	Acup+CM VS CM	4	1.00%
	M-Acup+RT VS Acup+RT	4	1.00%
	M-Acup+RT+Other VS RT+Other	1	0.25%
	M-Acup+Other VS Acup+Other	1	0.25%
	M-Acup+CM VS CM	1	0.25%
	M-Acup+TCM VS TCM	1	0.25%
Comparison of acupuncture and other therapies	Acup VS RT	29	7.21%
	Acup VS CM	20	4.98%
	Acup VS Blank	15	3.73%
	Acup+RT VS RT+Other	9	2.24%
	Acup VS TCM	9	2.24%
	M-Acup VS RT	7	1.74%
	Acup+RT VS RT+CM	6	1.49%
	M-Acup VS CM	6	1.49%
	Acup VS Other	4	1.00%
	M-Acup VS Blank	2	0.50%
	M-Acup VS TCM	2	0.50%
	Acup+RT VS TCM+RT	1	0.25%
	Acup+Other VS RT+Other	1	0.25%
	M-Acup+RT VS RT+Other	1	0.25%
	M-Acup+CM VS CM+Other	1	0.25%
	M-Acup VS Acup+CM	1	0.25%
	M-Acup VS Other	1	0.25%
The efficacy of acupuncture and moxibustion itself	Acup+RT VS Sham-Acup+RT	2	0.50%
	Acup VS Sham-Acup	2	0.50%

NOTE:
Acup: Single acupuncture therapy; M-Acup: Multiple acupuncture therapies; Sham-Acup: Sham acupuncture therapies; RT: Treatment of rehabilitation; CM: Conventional medicine; TCM: Traditional Chinese medicine; Blank: Only basic stroke therapy was used; Other: Other treatments other than those described above.

Appendix 7.Frequency statistics of intervention categories.

Categories of Intervention	count（n=402）	proportion
Acup+RT	151	37.6%
Acup	138	34.3%
M-Acup	42	10.4%
M-Acup+RT	20	5.0%
Acup+Other	18	4.5%
Acup+RT+Other	11	2.7%
Acup+TCM	10	2.5%
Acup+Western medicine	4	1.0%
Acup	2	0.5%
M-Acup+Western medicine	2	0.5%
Acup+TCM+RT	1	0.2%
M-Acup+RT+Other	1	0.2%
M-Acup+Other	1	0.2%
M-Acup+TCM	1	0.2%

Appendix 8.Frequency statistics of control categories.

Categories of Control	count （n=402）	proportion
RT	151	37.56%
Acup	80	19.90%
Acup+RT	33	8.21%
Western medicine	31	7.71%
RT+Other	23	5.72%
TCM	22	5.47%
Other	20	4.98%
Blank	17	4.23%
RT+Western medicine	6	1.49%
M-Acup	4	1.00%
M-Acup+RT	4	1.00%
Acup+Other	3	0.75%
Sham-acup	2	0.50%
Sham-acup+RT	2	0.50%
Acup+Western medicine	1	0.25%
Acup+TCM+RT	1	0.25%
Western medicine+Other	1	0.25%
TCM+RT	1	0.25%

Appendix 9.Statistics of acupuncture interventions included in RCTs.

Acupuncture-related intervention methods	count （n=402）	proportion
Body acupuncture	155	38.56%
EA	52	12.94%
Warm acupuncture	27	6.72%
Floating needle	20	4.98%
Body acupuncture+Scalp acupuncture	16	3.98%
Body acupuncture+Moxibustion	14	3.48%
Moxibustion	12	2.99%
Wrist-ankle needle	12	2.99%
Acupoint injection	11	2.74%
Abdominal acupuncture	9	2.24%
Intradermal needle	6	1.49%
Scalp acupuncture+EA	6	1.49%
Scalp acupuncture	5	1.24%
Balance acupuncture	4	1.00%
Body acupuncture+Blood-pricking therapy	4	1.00%
Body acupuncture+Scalp acupuncture+Wrist-ankle needle	4	1.00%
Elongated needle	3	0.75%
Body acupuncture+Catgut embedding therapy	3	0.75%
Acupoint Application	3	0.75%
Eyes acupuncture	3	0.75%
Acupotomy	3	0.75%
Ear acupuncture	2	0.50%
Blood-pricking therapy	2	0.50%
Body acupuncture+Scalp acupuncture+Moxibustion	2	0.50%
Body acupuncture+Balance acupuncture	2	0.50%
Body acupuncture+Scalp acupuncture	2	0.50%
Body acupuncture+Wrist-ankle needle	2	0.50%
Body acupuncture+Acupotomy	2	0.50%
Ear acupuncture+Balance acupuncture	1	0.25%
Sharp-hook needle	1	0.25%
Fire needle	1	0.25%
TAES	1	0.25%
Moxibustion+Blood-pricking therapy	1	0.25%
Skin needle	1	0.25%
Skin needle+Blood-pricking therapy	1	0.25%
Umbilical needle	1	0.25%
Intradermal needle+Balance acupuncture	1	0.25%
Body acupuncture+Abdominal acupuncture	1	0.25%
Body acupuncture+Fire needle	1	0.25%
Body acupuncture+Elongated needle	1	0.25%
Body acupuncture+Intradermal needle	1	0.25%
Body acupuncture+Body acupuncture	1	0.25%
Body acupuncture+Acupoint injection	1	0.25%
Catgut embedding therapy	1	0.25%

Appendix 10.Statistical analysis of the frequency of each acupuncture method in RCTs.

Acupuncture-related intervention methods	count （n=477）	proportion
Body acupuncture	213	44.65%
EA	58	12.16%
Scalp acupuncture	35	7.34%
Moxibustion	29	6.08%
Warm acupuncture	27	5.66%
Floating needle	20	4.19%
Wrist-ankle needle	18	3.77%
Acupoint injection	12	2.52%
Abdominal acupuncture	10	2.10%
Balance acupuncture	8	1.68%
Intradermal needle	8	1.68%
Blood-pricking therapy	8	1.68%
Acupotomy	5	1.05%
Elongated needle	4	0.84%
Catgut embedding therapy	4	0.84%
Ear acupuncture	3	0.63%
Acupoint Application	3	0.63%
Eyes acupuncture	3	0.63%
Fire needle	2	0.42%
Skin needle	2	0.42%
Intradermal needle	2	0.42%
Sharp-hook needle	1	0.21%
TAES	1	0.21%

Appendix 11. Statistical analysis of the frequency of each acupuncture method in RCTs.

Course of intervention	count （n=477）	proportion
<2w	11	3.24%
2W≤T<4w	226	66.67%
4W≤T<6w	37	10.91%
6W≤T<8w	14	4.13%
8W≤T<10w	15	4.42%
10W≤T<12w	1	0.29%
≥12w	9	2.65%
Not report	26	7.67%

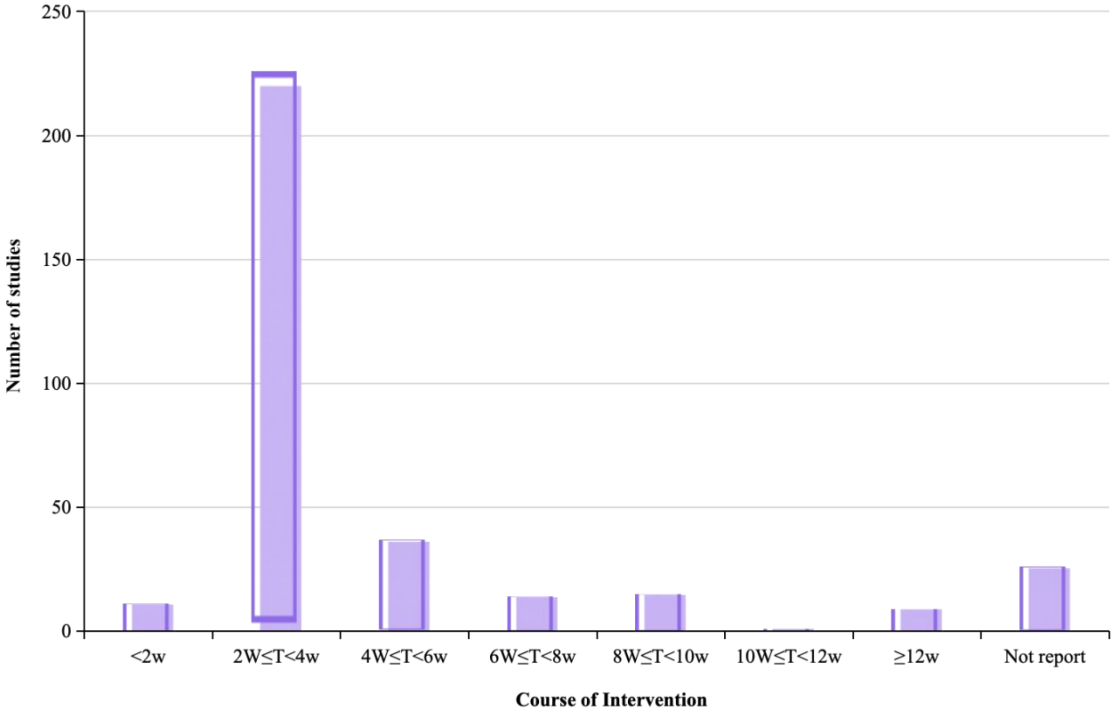

Appendix 12.Statistical analysis of the frequency of acupoint related information in RCTs.

Types of pain	Acupoints	Count	Meridians	Count	Body regions	Count	Specific points	Count
Pain of post-stroke	LI15	314	LI	1033	Shoulder	958	Sea Points	605
	LI11	250	TE	527	Upper arm	560	Crossing Points	475
	SJ14	231	GB	392	Forearm	513	Eight Confluent Points	392
	SJ5	199	SI	344	Cephalic region	503	Luo-Connecting Points	376
	LI4	197	ST	301	Lower leg region	389	Yuan-Source Points	315
	SI9	194	BL	203	Hand	360	Lower He-Sea Points	183
	ST36	133	GV	194	Ankle region	286	Eight Influential Points	158
	PC6	123	PC	165	Thigh	179	Well Points	113
	LI10	121	SP	144	ashi point	91	Stream Points	89
	SP6	108	HT	128	Abdomen	88	River Points	63
Shoulder pain of post-stroke	LI15	279	LI	897	Shoulder	894	/	/
	SJ14	211	TE	445	Upper arm	469	/	/
	LI11	209	SI	330	Forearm	425	/	/
	SI9	190	GB	269	Cephalic region	307	/	/
	LI4	167	ST	205	Lower leg region	300	/	/
	SJ5	162	PC	132	Hand	298	/	/
	LI10	106	GV	128	Ankle region	201	/	/
	PC6	100	BL	124	Thigh	107	/	/
	EX-UE12	99	SP	114	Abdomen	85	/	/
	ST36	98	HT	105	ashi point	85	/	/
Thalamic pain of post-stroke	DU26	14	GB	40	Cephalic region	56	/	/
	PC6	14	TE	39	Upper arm	35	/	/
	MS7	13	BL	35	Forearm	33	/	/
	BL40	10	LI	23	Thigh	31	/	/
	HT1	10	ST	20	Ankle region	30	/	/
	SJ5	10	PC	19	Hand	22	/	/
	SP6	10	HT	18	Knee region	19	/	/
	BL36	9	GV	17	Shoulder	19	/	/
	BL62	9	SP	13	Buttocks	18	/	/
	EX-LE2	9	KI	9	Lower leg region	18	/	/
	GB30	9	/	/	/	/	/	/
	GB40	9	/	/	/	/	/	/
	LI11	9	/	/	/	/	/	/
	LI15	9	/	/	/	/	/	/
	SJ14	9	/	/	/	/	/	/
	SJ4	9	/	/	/	/	/	/
	ST31	9	/	/	/	/	/	/
	ST35	9	/	/	/	/	/	/

Central post-stroke pain	GB20	16	LI	46	Cephalic region	97	/	/
	LI11	15	GV	38	Lower leg region	29	/	/
	ST36	14	GB	37	Forearm	26	/	/
	SJ5	13	ST	29	Upper arm	24	/	/
	DU20	11	BL	23	Ankle region	17	/	/
	LI4	10	TE	17	Hand	16	/	/
	DU24	9	PC	8	Thigh	13	/	/
	GB34	9	KI	6	Shoulder	12	/	/
	LI15	8	SP	6	Buttocks	10	/	/
	BL4	6	CV	5	Nuchal region	8	/	/
	DU16	6	/	/	/	/	/	/
	KI1	6	/	/	/	/	/	/
	LI14	6	/	/	/	/	/	/

Appendix 13.The high-frequency links of selected acupoint,Meridian, body parts and specific points in 484 acupuncture prescriptions.

Post-stroke pain							
Acupoints		Meridians		Body regions		Specific points	
High-frequency Link	frequency	High-frequency Link	frequency	High-frequency Link	frequency	High-frequency Link	frequency
LI15 -SJ14	214	LI -TE	315	Forearm- Upper arm	292	Eight Confluent Points-Luo-Connecting Points	246
LI11 -LI15	204	LI -SI	228	Shoulder-Upper arm	265	Eight Confluent Points-Sea Points	239
LI15 -SI9	176	SI -TE	217	Forearm- Shoulder	223	Luo-Connecting Points- Sea Points	239
LI11 -SJ5	174	GB -LI	162	Hand-Upper arm	205	Crossing Points- Sea Points	226
LI15 -SJ5	163	Ex - point-s-LI	155	Forearm- Hand	202	Crossing Points- Luo-Connecting Points	206
LI11 -LI4	157	GB -ST	144	Hand-Shoulder	184	Crossing Points- Eight Confluent Points	204
SI9 -SJ14	149	LI -ST	142	Lower leg region-Upper arm	164	Sea Points-Yuan-Source Points	191
LI15 -LI4	148	GB -TE	140	Forearm- Lower leg regio	162	Eight Confluent Points-Yuan-Source Points	180
LI11 -SJ14	146	Ex - point-s-TE	137	Cephalic region- Forearm	130	Luo-Connecting Points- Yuan-Source Points	171
LI4 -SJ5	127	ST -TE	110	Cephalic region- Upper arm	122	Crossing Points- Yuan-Source Points	160
SJ14 -SJ5	115	LI -PC	101	Cephalic region- Lower leg r	116	Lower He-Sea Points- Sea Points	150
LI10 -LI11	108	PC -SP	95	Ankle region-Forearm	111	Crossing Points- Lower He-Sea Points	142
LI10 -LI15	106	BL -GB	94	Forearm- Thigh	107	Lower He-Sea Points- Luo-Connecting Points	137
LI11 -SI9	105	Ex - points- SI	93	Ankle region-Upper arm	107	Eight Confluent Points-Lower He-Sea Points	135
LI4 -SJ14	96	BL -LI	92	Lower leg region-Shoulder	105	Eight Influential Points-Sea Points	103
EX-UE12 -LI15	91	Ex - points- GB	86	Ankle region-Lower leg reg	103	Lower He-Sea Points- Yuan-Source Points	98
LI14 -LI15	89	SP -ST	86	Thigh- Upper arm	99	Eight Influential Points-Luo-Connecting Point	92
PC6 -SP6	89	GV -PC	84	Hand-Lower leg region	97	Crossing Points- Eight Influential Points	90
LI10 -LI4	87	HT -PC	83	Ankle region-Cephalic regi	89	Eight Confluent Points-Eight Influential Poi	90
LI14 -SJ14	85	GB -SP	81	Lower leg region-Thigh	87	Eight Influential Points-Lower He-Sea Points	89
LI11 -ST36	82	BL -ST	78	Ankle region-Hand	85	Eight Influential Points-Yuan-Source Points	75
LI10 -SJ5	82	GV -SP	77	ashi point-Shoulder	77	Stream Points- Yuan-Source Points	59
SI9 -SJ5	81	HT -SP	77	Cephalic region- Hand	77	Eight Confluent Points-Stream Points	57
LI4 -SI9	80	LI -SP	77	Ankle region-Shoulder	73	Sea Points-Stream Points	52
LI11 -LI14	78	GB -GV	77	Cephalic region- Shoulder "	70	Luo-Connecting Points- Stream Points	46
LI10 -SJ14	77	GB -PC	77	Cephalic region- Thigh	68	Eight Confluent Points-Well Points	45
DU26 -PC6	73	PC -ST	76	Hand-Thigh	63	Crossing Points- Well Points	43
EX-UE12 -SJ14	72	PC -TE	75	Shoulder-Thigh	62	Luo-Connecting Points- Well Points	43
EX-UE12 -SI9	71	ashi point-LI	74	Ankle region-Thigh	56	Sea Points-Well Points	43

Post-stroke shoulder pain					
Acupoints		Meridians		Body regions	
High-frequency Link	frequency	High-frequency Link	frequency	High-frequency Link	frequency
LI15-SJ14	195	LI-TE	276	Forearm-Upper arm	242
LI11-LI15	173	LI-SI	218	Shoulder-Upper arm	232
LI15-SI9	172	SI-TE	207	Forearm-Shoulder	191
SI9- SJ14	145	Ex - points-LI	132	Hand- Upper arm	171
LI11-SJ5	142	GB-LI	122	Forearm-Hand	166
LI11-LI4	136	Ex - points-TE	118	Hand- Shoulder	156
LI15-SJ5	136	GB-TE	105	Lower leg region-Upper arm	123
LI15-LI4	130	GB-ST	104	Forearm-Lower leg region	119
LI11-SJ14	127	LI-ST	99	Cephalic region- Forearm	94
LI4- SJ5	108	Ex - points-SI	92	Cephalic region- Upper arm	89
LI11-SI9	101	LI-PC	84	Cephalic region- Lower leg region	83
SJ14-SJ5	99	PC-SP	76	Lower leg region-Shoulder	82
LI10-LI11	94	ST-TE	73	Ankle region-Forearm	81
LI10-LI15	93	SP-ST	72	Ankle region-Lower leg region	79
EX-UE12- LI15	89	ashi point-LI	69	Ankle region-Upper arm	78
LI4- SJ14	89	HT-PC	68	ashi point-Shoulder	73
LI14-LI15	84	GB-SP	67	Hand- Lower leg region	70
LI14-SJ14	81	ashi point-TE	66	Forearm-Thigh	66
LI4- SI9	77	GB-SI	66	Thigh-Upper arm	62
SI9- SJ5	77	PC-TE	63	Ankle region-Cephalic region	62
LI10-LI4	76	GV-PC	62	Lower leg region-Thigh	56
LI10-SJ5	72	LI-SP	62	Ankle region-Hand	55
EX-UE12- SI9	71	PC-ST	62	Cephalic region- Shoulder	53
PC6- SP6	71	GB-PC	62	Ankle region-Shoulder	51
EX-UE12- SJ14	70	ashi point-SI	61	Cephalic region- Hand	50
LI10-SJ14	70	Ex - points-GB	61	Back- Shoulder	49
LI11-LI14	69	BL-LI	60	ashi point-Upper arm	48
LI14-SI9	63	GV-SP	60	Cephalic region- Thigh	43
ashi point-LI15	63	HT-SP	60	Hand- Thigh	42

Post-stroke thalamic pain					
Acupoints		Meridians		Body regions	
High-frequency Link	frequency	High-frequency Link	frequency	High-frequency Link	frequency
DU26-PC6	14	BL-GB	28	Forearm-Thigh	21
BL40-DU26	10	GB-LI	26	Forearm-Upper arm	20
BL40-HT1	10	GB-ST	25	Thigh-Upper arm	19
BL40-PC6	10	GB-TE	25	Cephalic regionForearm	17
BL40-SP6	10	LI-ST	25	Cephalic regionUpper arm	16
DU26-HT1	10	LI-TE	24	Cephalic regionThigh	15
DU26-SP6	10	ST-TE	23	Forearm-Hand	13
HT1-PC6	10	BL-LI	22	Forearm-Lower leg region	12
HT1-SP6	10	BL-GV	21	Lower leg regionUpper arm	12
PC6-SP6	10	GV-PC	20	Ankle regionForearm	12
BL36-BL62	9	Ex - points-GB	20	Ankle regionHand	12
BL36-EX-LE2	9	BL-ST	19	Hand-Upper arm	12
BL36-GB30	9	BL-TE	19	Cephalic region Lower leg region	11
BL36-GB40	9	Ex - points-LI	18	Lower legregionThigh	11
BL36-LI11	9	GB-GV	17	Ankle regionUpper arm	11
BL36-LI15	9	Ex - pointsST	17	Hand-Thigh	11
BL36-SJ14	9	Ex - pointsTE	17	Ankle regionKnee region	10
BL36-SJ4	9	BL-PC	16	Ankle regionThigh	10
BL36-SJ5	9	HT-SP	15	Cephalic region Hand	10
BL36-ST31	9	PC-SP	15	Forearm-Knee region	10
BL36-ST35	9	/	/	Hand-Knee region	10
BL62-EX-LE2	9	/	/	Knee region =Upper arm	10
BL62-GB30	9	/	/	/	/
BL62-GB40	9	/	/	/	/
BL62-LI11	9	/	/	/	/
BL62-LI15	9	/	/	/	/
BL62-SJ14	9	/	/	/	/
BL62-SJ4	9	/	/	/	/
BL62-SJ5	9	/	/	/	/

Post-stroke central pain					
Acupoints		Meridians		Body regions	
High-frequency Link	frequency	High-frequency Link	frequency	High-frequency Link	frequency
LI11-ST36	12	GB-LI	15	Cephalic region-Forearm	14
LI11-SJ5	11	GB-ST	14	Cephalic region-Lower leg regio	14
SJ5-ST36	10	LI-ST	14	Forearm- Upper arm	14
GB20-LI11	9	BL-GB	14	Lower leg region-Upper arm	13
GB20-ST36	9	GB-TE	13	Cephalic region-Upper arm	11
DU20-DU24	9	LI-TE	13	Forearm-Hand	11
LI11-LI4	9	ST-TE	12	Ankle region-Cephalic region	10
LI4-ST36	9	Ex- points-GB	11	Ankle region-Forearm	10
GB20-SJ5	8	BL-LI	9	Ankle region- Lower leg region	10
GB34-LI11	8	GB-GV	9	Ankle region-Upper arm	10
GB34-LI4	8	Ex - points -LI	8	Forearm- Thigh	10
LI11-LI15	8	Ex - points -ST	8	Hand- Lower leg region	10
GB34-ST36	7	Ex - points -YE	8	Hand- Upper arm	10
LI4-SJ5	7	BL-GV	8	Lower leg region-Thigh	9
LI15-LI4	7	BL-ST	8	Cephalic region-Hand	9
LI15-SJ5	7	BL-TE	8	Cephalic region-Thigh	8
LI11-LI14	6	GV-PC	6	Thigh-Upper arm	8
BL4-DU16	6	Ex -points -GV	6	Cephalic region-Nuchal region	8
BL4-DU20	6	Ex - points -KI	5	Forearm- Shoulder	8
BL4-DU24	6	Ex-points-Scalp acupuncture	5	Hand-Shoulder	8
BL4-GB20	6	GB-KI	5	Shoulder-Upper arm	8
DU16-DU20	6	GB-Scalp acupuncture	5	/	/
DU16-DU24	6	KI-LI	5	/	/
DU16-GB20	6	KI-Scalp acupuncture	5	/	/
DU20-GB20	6	KI-ST	5	/	/
DU24-GB20	6	KI-TE	5	/	/
GB34-LI15	6	LI-Scalp acupuncture	5	/	/
GB34-SJ5	6	Scalp-acupuncture-ST	5	/	/
LI15-ST36	6	Scalp acupuncture-TE	5	/	/

Appendix 14.Details of the outcome measures of the included RCTs.

Ref. No.	Outcom 1	type	Outcome 2	type	Outcome 3	type	Outcome 4	type	Outcome 5	type	Outcome 6	type	Outcome 7	type	Outcome 8	type	Outcome 9	type	Outcome 10	type
	VAS	1	FMA	2	ROM Goniometer [Maximum Angles of Flexion, Extension, External Rotation, Internal Rotation, Abduction, Adduction]	5	ADL	3												
2	NFDS	2	VAS	1	FMA	2	Effective Rate	6												
3	VAS	1	PRS	1	PROM	2	MMT	2	MAS	2	MBI	3	Safety and adverse effects	7						
4	VAS	1	SPADI	1	WOMAC	1	ROM	5	10-Meter Walk Test (10MWT)	5	FGA	2	Manual Function Test (MFT)	2	ADL	3	Instrumental Activities of Daily Living (IADL)	3	ROM【Shoulder Flexion (SF), Shoulder Extension (SE), Shoulder Abduction (SAB), Shoulder Adduction (SAD), Knee Flexion (KF)】	5
5	Objectiv	5	Effective Rate	6																

	e Indicators【Hemorheology】																			
6	NRS	1	PROM	5	Fugl-Meyer Assessment (FMA) for Motor Function	2	MPQ-SF	3	Safety and adverse effects	7										
7	VAS	1	Musculoskeletal Sonography System【AHD, AGT, ALT】	5																
8	Degree of edema	2	Shoulder Joint Degree of pain	1	VAS	1	FMA-U	2	BI	3										
9	VAS	1	NIHSS	2	FMA	2	BI	3	HAMD-17	2	Safety and adverse effects	7								
10	Effective Rate	6	VAS	1	MBI	3	MRS	2												
11	Degree of pain	1	Effective Rate	6																
12	SF-MPQ	3	Effective Rate	6	SDS	2	PSQI	2	Safety Assessment	7										
13	VAS	1	SF-MPQ	3	Effective Rate	6														
14	Effective Rate	6	VAS	1	SF-MPQ	3	SF-36	3												
15	VAS	1	SF-MPQ	3	Safety	7	Compliance	10												
16	Effective Rate	6	VAS	1	SF-MPQ	3														
17	Effectiv	6	VAS	1	FMA	2	MBI	3												

	e Rate																			
18	VAS	1	FM	2	ADL	3														
19	VAS	1	BI	3	Three-Level Balance Diagnosis	2														
20	MBI	3	FMA	2	Effective Rate	6														
21	ROM	5	VAS	1	FMA	2	FIM	2	Safety and adverse effects	7										
22	MBI	3	FMA-U	2	VAS	1	Effective Rate	6												
23	VAS	1	Shoulder Pain Score	1	CMS	1	ADL	3	Effective Rate	6										
24	VAS	1	PPI	1	PRI	1	Effective Rate	6												
25	VAS	1	ROM	5	FMA	2														
26	VAS	1	SPADI	1	Goniometer [Severity of Pain, Daily Function, and Shoulder Range of Motion (ROM)]	5	Safety and adverse effects	7												
27	FMA	2	ROM	5																
28	NRS	1	FMA	2	ADL	3														
29	FMA	2	Activity Score	2	Pain Score	1	Motor Function Score	2												
30	VAS	1	NIHSS	2	BI	3	Effective Rate	6												
31	VAS	1	FMA	2	Edema Level	2	Effective Rate	6												
32	VAS	1	ROM	5	BI	3	MAS	2	Safety and adverse effects	7										
33	Effective Rate	6	Whole blood viscosity	5																

34	GLASGOW Coma Scale	2	Degree of clinical neurological impairment	2	ADL	3	Effective Rate	6												
35	Effective Rate	6	VAS	1	FMA	2														
36	VAS	1	FMA	2	BI	3	Effective Rate	6												
37	SF-MPQ	3	FMA	2																
38	Shoulder pain score table	1																		
39	VAS	1	MBI	3																
40	VAS	1																		
41	VAS	1	FMA	2	ADL	3														
42	Effective Rate	6																		
43	VAS	1	MBI	3	ROM	5														
44	VAS	1	FMA	2	BI	3	Effective Rate	6												
45	SF-MPQ	3	Transcranial Doppler ultrasound [blood flow velocity and direction of the posterior cerebral artery (PCA), vertebral artery (VA), and basilar artery (BA)]	5	Effective Rate	6														
46	TCM syndrom	4	FMA	2	ADL	3	VAS	1	Effective Rate	6										

	e score																			
47	VAS	1	ROM	5	FMA	2														
48	PRI	1	VAS	1	PPI	1	Effective Rate	6												
49	VAS	1	FMA	2	MBI	3	Effective Rate	6												
50	VAS	1	FMA	2	MBI	3														
51	Effective Rate	6																		
52	Effective Rate	6	VAS	1	FMA	2	CMS	1	MBI	3	ROM	5								
53	VAS	1	FM	2	MBI	3	Effective Rate	6												
54	VAS	1	FMA	2	MBI	3														
55	Measurement of somatosensory evoked potentials	5	VAS	1	FMA	2														
56	VAS	1																		
57	VAS	1	FMA	2	MBI	3														
58	ROM	5	VAS	1	FMA	2														
59	FMA	2	BI	3	Effective Rate	6														
60	VAS	1	SF-36	3	PSQI	2	Objective Indicators【Laboratory examination（Cx 43】	5												
61	Effectiv	6	VAS	1	FMA	2	BI	3												

	e Rate																			
62	FCA	3	NFDS	2	FCA	3	VAS	1	BI	3	Objective Indicators【Laboratory examination（SPP GE2】	5	Effective Rate	6						
63	VAS	1	Headache score	1	Effective Rate	6														
64	Neurological score	2	Effective Rate	6	SF-36	3	VAS	1												
65	VAS	1	ROM	5																
66	Objective Indicators【Laboratory examination（Blood lipids)】	5	Effective Rate	6																
67	Effective Rate	6	Objective Indicators【Laboratory examination Whole blood viscosity】	5																
68	VAS	1	ROM	5	FMA	2														
69	VAS	1	Effective Rate	6																
70	VAS	1	MRS	2	Fugl-Meyer Assessment (FMA) Sensory Function	2	Motor Impairment Score	2	Current Threshold Detector [Sensory Threshold	5										

									(CPT)]											
71	Effective Rate	6	Objective Indicators【Hemorheology(High cut whole blood viscosity, low cut whole blood viscosity, plasma viscosity and fibrinogen)】	5	NRS	1	NFDS	2	FCA	3	ADL	3	Safety and adverse effects	7						
72	VAS	1	Headache score	1	HIT-6	3	Effective Rate	6												
73	VAS	1																		
74	Effective Rate	6	Skin Lesion	2	Vital Signs	2	Blood Parameters	5	Satisfaction	9										
75	VAS	1	FMA	2	MBI	3	Effective Rate	6												
76	Effective Rate	6																		
77	NRS	1	FMA-U	2	ROM	5														
78	Effective Rate	6	NIHSS	2	FAC	2	NFDS	2	FMA-U	2										
79	VAS	1	FMA	2	ROM	5	FLEX-SF	1	Effective Rate	6										
80	VAS	1	FM	2	Effective Rate	6	Clinical Neurological Deficit Degree Rating Scale	2												
81	VAS	1	FMA	2																
82	VAS	1																		

83	VAS	1	FMA	2	Effective Rate	6														
84	Effective Rate	6																		
85	Effective Rate	6	VAS	1	FMA	2	ROM	5	SHSS	1	Objective Indicators【Laboratory examination（CRP，NO】	5								
86	Effective Rate	6																		
87	VAS	1	FMA	2	ADL	3	Effective Rate	6												
88	VAS	1	FMA	2	Effective Rate	6														
89	PRI	1	VAS	1	PPI	1	Objective Indicators【Laboratory examination（IL-6，TNF-α，NO】	5	Effective Rate	6										
90	VAS	1	FMA	2	NIHSS	2														
91	VAS	1																		
92	VAS	1	Effective Rate	6																
93	VAS	1	FMA	2	PROM	5	MBI	3	Effective Rate	6										
94	NFDS	2	FCA	3	SF-36	3	Effective Rate	6	Adverse Event (AE)	7										
95	VAS	1	Clinical	1	PSQI	2	Quality	3												

			symptoms integral				of Life Score													
96	Effective Rate	6																		
97	Assessment of the degree of shoulder subluxation	1	Musculoskeletal ultrasound【AGT】	5	VAS	1	FMA	2	Effective Rate	6										
98	VAS	1	Effective Rate	6																
99	VAS	1	FMA	2	MBI	3														
100	FMA	2	BI	3	VAS	1														
101	VAS	1	PROM	5	Effective Rate	6														
102	VAS	1	MAS	2	FMA	2	MBI	3												
103	VAS	1	FM	2	MBI	3														
104	VAS	1	PSQI	2	Transcutaneous Sensory Nerve Quantification Detector [Tolerance Threshold, Sensory Threshold]	5	Objective Indicators【Laboratory examination（IL-1β, CXCL10】	5												
105	Effective Rate	6	VAS	1	FMA-U	2	MBI	3												
106	Upper limb neurological	2	Patient's Living Ability Status	3																

	deficit score																			
107	VAS	1	FMA-U	2	STAI	2	Safety and adverse effects	7												
108	Effective Rate	6																		
109	VAS	1	FMA	2	BI	3	Effective Rate	6												
110	VAS	1	FMA	2	MBI	3														
111	VAS	1	PPI	1	PRI	1														
112	VAS	1	NIHSS	2	Effective Rate	6														
113	VAS	1	Effective Rate	6																
114	Effective Rate	6	FMA	2	VAS	1	MBI	3	ROM	5										
115	VAS	1	FMA	2	MBI	3	Objective Indicators【Laboratory examination（IL-6，TNF-α，NO】	5												
116	VAS	1	MBI	3	Frequency of Shoulder Pain	1	Evaluation of Analgesic Use	10	Effective Rate	6										
117	Limb pain score	1																		
118	VAS	1	FMA-U	2																
119	VAS	1	PROM	5	Upper limb muscle	2	MBI	3	FMA	2										

					strength															
120	VAS	1																		
121	VAS	1	FMA	2	Frequency of Shoulder Pain	1	Satisfaction Assessment	9												
122	VAS	1	frequency of shoulder pain	1	satisfaction degree	9	Correlation between average duration of thermosensitization and degree and frequency of pain	1												
123	VAS	1	FMA	2	FIM	2														
124	Clinical symptoms	1	VAS	1	Safety and adverse effects	7														
125	VAS	1	FMA	2	MBI	3														
126	VAS	1	FMA	2	BI	3	Objective Indicators【Laboratory examination（IL-1β】	5	Effective Rate	6										
127	NRS	1	FMA	2	ROM	5	BI	3												
128	VAS	1	BI	3	SDS	2	SAS	2	Effective Rate	6										

129	Shoulder function rating scale	2	FMA	2	Objective Indicators【Laboratory examination（IL一1β】	5														
130	Effective Rate	6																		
131	Effective Rate	6	VAS	1	FMA	2	BI	3												
132	Effective Rate	6																		
133	Effective Rate	6	Brunnstrom评定	2	MAS	2	ROM	5	VAS	1	FMA	2	MBI	3						
134	Effective Rate	6	FMA-U	2	MBI	3	SHSS	1	Objective Indicators【Laboratory examination（hs-CRP，IL-6】	5										
135	VAS	1	ROM Angle	5	FMA	2	FIM	2	MAS	2										
136	VAS	1	FMA Sensory Function	2	FMA Motor Function	2	Current Perception Threshold (CPT) Testing Device	5	MRS	2										
137	VAS	1																		
138	VAS	1	CMS	1	Effective Rate	6														
139	FMA	2	VAS	1	Effective Rate	6														
140	Effective Rate	6																		

141	VAS	1	FMA	2	MBI	3														
142	Limb pain score	1																		
143	VAS	1	Measurement of upper limb muscle strength	2	FMA	2	MBI	3	MAS	2										
144	VAS	1	Effective Rate	6																
145	Headache score	1	VAS	1	Effective Rate	6	recurrence rate	10												
146	NRS	1	MBI	3	FMA	2														
147	Effective Rate	6																		
148	VAS	1	SPADI	1	CMS	1	MBI	3												
149	BI	3	FMA	2	VAS	1	ROM	5	Treatment Satisfaction	9										
150	VAS	1	FMA	2																
151	Limb pain score	1																		
152	MAS	2	MMT	2	Brunnstrom	2														
153	VAS	1	Effective Rate	6	Pain relief situation	1	Objective Indicators【（Blood lipids）】	2	Effective Rate	6										
154	VAS	1	Effective Rate	6																
155	Effective Rate	6	Nursing Satisfaction	9	VAS	1														
156	VAS	1	Joint Range of Motion	2	GQOLI-74	3	Effective Rate	6												
157	VAS	1	FMA	2	Safety and adverse effects	7														

158	NDS	2	BI	3																
159	Effective Rate	6	VAS	1	ROM	5	FMA	2	BI	3	BBS	2								
160	VAS	1	FMA	2	Effective Rate	6														
161	VAS	1	FMA	2	MBI	3	ROM	5	Effective Rate	6										
162	VAS	1	FMA-U	2	MBI	3	PSQI	2												
163	Effective Rate	6	ROM	5	VAS	1	NIHSS	2												
164	VAS	1	Headache score	1	Content of pain-related factors in plasma	5														
165	VAS	1	FMA-U	2	MBI	3	Effective Rate	6												
166	BI	3	VAS	1	Use of Analgesic Drugs	10														
167	VAS	1	Brunnstrom评定	2																
168	VAS	1	FMA	2																
169	VAS	1	FMA	2	Quantitative Musculoskeletal Ultrasound Assessment	5	Effective Rate	6	Safety Evaluation	7										
170	VAS	1	FMA	2	Index of instrument【Musculoskeletal ultrasound [Depth of effusion of tendon sheath of long head of biceps brachii tendon, thickness of long head of biceps brachii tendon, thickness of subscapularis tendon, distance between acromion and greater tuberosity]】	5														

171	VAS	1	PROM	5	Effective Rate	6														
172	VAS	1	CMS	1	FMA	2														
173	BI	3	SF-36	3	Effective Rate	6														
174	Effective Rate	6	ROM	5	SF-36	3														
175	VAS	1	PPI	1	CMS	1	FMA	2	Effective Rate	6	Adverse event	7								
176	VAS	1	SF-MPQ	3	FMA	2	Effective Rate	6												
177	BI	3	VAS	1	Effective Rate	6														
178	VAS	1	PPI	1	PRI	1	TCM syndrome score	4												
179	Safety and adverse effects	7	VAS	1	FMA-U	2	MBI	3												
180	Objective Indicators【Laboratory examination（WBC, ALT, AST)】	5	headache TCM syndrome score	4																
181	VAS	1																		
182	NRS	1	FMA	2	BI	3	Effective Rate	6												
183	VAS	1	FMA	2	QOL-BREF	3														
184	VAS	1	HAMA	2	HAMD	2														
185	VAS	1	Symptom Score	1	DASH	2	FMA	2	MBI	3	ADL	3	Effective Rate	6						

186	SF-MPQ	3	Objective Indicators【Laboratory examination（血浆β-EP含量】	5	Effective Rate	6														
187	VAS	1	FMA	2	MSS	2	WMFT	2	MBI	3										
188	FMA	2	VAS	1	GQOLI-74	3	Swelling Score	5												
189	VAS	1	CMS	1	BI	3														
190	NIHSS	2	Score of Shoulder Ultrasound Examination for Lesions	5	VAS	1	BI	3	HADS	2										
191	VAS	1	MBI	3																
192	MPQ	3	VAS	1	PPI	1	PRI	1												
193	VAS	1	BI	3	DASH	2	FMA-U	2	Compliance	10	GQOLI-74	3	Effective Rate	6						
194	VAS	1	FMA	2	MBI	3	Effective Rate	6												
195	VAS	1	Melle Score	2	BI	3	Effective Rate	6												
196	FMA-U	2	MBI	3	PROM	5														
197	VAS	1	FMA	2	MBI	3	Effective Rate	6												
198	Effective Rate	6	VAS	1	CMS	1	ADL	3												
199	Effective Rate	6	VAS	1	FMA-U	2	MBI	3	Effective Rate	6										
200	Degree of pain relief	1	ROM	5																
201	Effective Rate	6	VAS	1	BI	3	FMA	2	SDS	2	SAS	2								
202	FMA	2	Wong-Banker	1																

			facial expression scale method																	
203	VAS	1	FMA	2	ROM	5														
204	VAS	1																		
205	Passive forward flexion of the shoulder joint	1	ROM	5																
206	Effective Rate	6																		
207	VAS	1	FMA	2	MBI	3														
208	VAS	1	FMA	2	Acromio-Humeral Interval（AHI）	5	Effective Rate	6												
209	VAS	1	FMA	2	Effective Rate	6														
210	NRS	1	BI	3	FMA	2														
211	Effective Rate	6																		
212	Effective Rate	6																		
213	Effective Rate	6																		
214	VAS	1	FMA	2	MBI	3	ROM	5												
215	SF-MPQ	3	VAS	1	Safety and adverse effects	7	Effective Rate	6												
216	VAS	1	FMA-U	2																
217	VAS	1																		
218	VAS	1	FMA	2	CMS	1	Objective Indicators【Laboratory examinat	5	Effective Rate	6										

							ion(βEP, ENK,Dyn）】													
219	肌力	2	MRS	2	Brunnstrom评定	2	VAS	1	Swelling score	5	FMA	2	MBI	3	ASS	1	Effective Rate	6		
220	Effective Rate	6																		
221	VAS	1	ROM	5																
222	VAS	1	FMA	2																
223	VAS	1	FMA	2																
224	VAS	1	MBI	3	FMA	2	ROM	5	Hemorheology指标	5	Effective Rate	6								
225	FMA	2	VAS	1	MAS	2	Hand Swelling	2												
226	VAS	1	Satisfaction Score	9	Objective Indicators【Laboratory examination（PGE2，SP，IL-8)】	5														
227	VAS	1	MBI	3	FMA	2	Effective Rate	6	PROM	5										
228	VAS	1																		
229	Effective Rate	6	VAS	1	FMA	2	Objective Indicators【Laboratory examination（IL-6，TNF-α】	5												
230	VAS	1	PPI	1	PRI	1	ADL	3	Objective Indicators【Laboratory	5										

									examination（Whole blood viscosity, fibrinogen, and hematocrit levels】											
231	VAS	1	NIHSS	2	Effective Rate	6														
232	VAS	1	FMA	2																
233	VAS	1	SF-MPQ	3	ES	2														
234	VAS	1	PPI	1	PRI	1	HAMD	2	Content of plasma β - endorphin (β - EP)	5										
235	Effective Rate	6	Objective Indicators【Laboratory examination（PGE2,SP)】	5																
236	VAS	1	PPI	1	PRI	1	Score of Pain Emotion Change	2	Clinical Neurological Deficit Score	2	FMA	2	BI	3						
237	FMA-U	2	PRI	1	PPI	1	VAS	1	Effective Rate	6	TCM Syndrome Score	4	Shoulder-Hand Syndrome Score	2	Safety and adverse effects	7				
238	Effective Rate	6	MBI	3																
239	VAS	1	Clinical Degree of Upper Limb Neurological Deficit	2	ADL	3	Objective Indicators【Hemorheology（Whol	5	Effective Rate	6										

							e blood viscosity ）】													
240	VAS	1	FMA	2	ADL	3	muscle strength	2	Effective Rate	6										
241	VAS	1	Effective Rate	6	Safety and adverse effects	7														
242	VAS	1	FMA	2	QOL-BREF	3	Effective Rate	6												
243	NRS	1																		
244	VAS	1	TCD【Changes in cerebral hemodynamic 】	5	EEG	5														
245	SF-MPQ	3	Shoulder Range of Motion (Shoulder ROM)	1	FMA	2														
246	SF-MPQ	3	FMA	2																
247	VAS	1	FMA	2	ADL	3														
248	VAS	1	FMA	2																
249	VAS	1	FMA	2	MBI	3														
250	VAS	1	ROM	5																
251	VAS	1	ROM	5	MAS	2	BI	3												
252	肌力	2	Effective Rate	6	VAS	1														
253	Effective Rate	6	FMA-U	2	ROM (Range of Motion) 【Active Abduction and Forward Flexion of the Shoulder Joint】	5	VAS	1	MBI	3	Objective Indicators【Laboratory examination（IL-1β】	5								
254	VAS	1	FMA	2	Effective Rate	6														
255	VAS	1	Effective Rate	6																
256	VAS	1	FMA	2	ADL	3	WMFT	2	Effective Rate	6	Safety and adverse effects	7								

257	VAS	1	FMA	2																
258	VAS	1	FMA	2																
259	Effective Rate	6	VAS	1	FMA	2	ROM	5												
260	PRI	1	VAS	1	PPI	1	Intracranial Vascular Detection [Blood flow velocities of the basilar artery (BA) and vertebral artery (VA) [peak velocity (Vs), mean velocity (Vm), end - diastolic velocity (Vd)], vascular pulsatility index (PI), vascular resistance index	5	Effective Rate	6										

							(RI)]													
261	VAS	1	PSQI	2	Effective Rate	6	Satisfaction	9												
262	VAS	1	FMA	2	Effective Rate	6														
263	Effective Rate	6	Neurological Deficit	2	BI	3														
264	Effective Rate	6	VAS	1	ROM	5	Frequency of pain	1												
265	VAS	1	FMA	2	MBI	3	Effective Rate	6												
266	Effective Rate	6	SF-36	3																
267	Effective Rate	6	VAS	1	Frequency of pain评分	1	FMA	2												
268	VAS	1	MAS	2	BI	3	Effective Rate	6												
269	Effective Rate	6																		
270	Effective Rate	6																		
271	VAS	1	FMA	2	MBI	3														
272	Effective Rate	6	VAS	1	ROM	5														
273	VAS	1	FMA	2	Effective Rate	6	Scale for Evaluating the Degree of Clinical Neurological Deficit	2												
274	VAS	1	CMS	1	MBI	3														
275	Effectiv	6	TCM Symptom	4	CMS	1	Quality	3												

	e Rate		Score				of Life Level													
276	VAS	1	Hand Swelling Measurement	2	FMA-U	2	BI	3												
277	VAS	1	PPI	1	PRI	1	Objective Indicators【Laboratory examination（IL-6，TNF-α】	5												
278	VAS	1	ROM	5																
279	Effective Rate	6	VAS	1	Frequency of pain	1	FMA	2	Patient Satisfaction	9										
280	VAS	1	PPI	1	PRI	1														
281	FMA	2	ROM	5																
282	SF-MPQ	3	WMFT	2	MBI	3														
283	VAS	1	BI	3	UE-FMA	2	ROM	5	Effective Rate	6										
284	SF-MPQ	3	FMA-U	2	ROM	5														
285	PRI	1	VAS	1	PPI	1	FMA-U	2	Objective Indicators【Hemorheology（Plasma viscosity, high cut whole blood viscosity,	5	Effective Rate	6								

									mid cut whole blood viscosity, low cut whole blood viscosity）】											
286	ROM	5	FMA	2	MBI	3	Oxford Handicap Scale (OHS)	10	Effective Rate	6										
287	Effective Rate	6	VAS	1																
288	VAS	1	FM	2	FIM	2														
289	VAS	1	FMA	2	BI	3	Effective Rate	6												
290	Effective Rate	6	ROM	5	VAS	1	FMA-U	2	FIM	2	Safety and adverse effects	7								
291	FMA	2	Upper limb pain assessment	1	ROM	5	Effective Rate	6												
292	VAS	1	PSQI	2	Effective Rate	6	Objective Indicators【Laboratory examination（S100β, NSE】	5	Safety and adverse effects	7										
293	VAS	1	FMA	2	ROM	5	Effective Rate	6												

294	VAS	1	ROM	5	FMA	2														
295	VAS	1	FMA	2	GQOLI-74	3	Effective Rate	6												
296	VAS	1	GQOLI-74	3	FMA	2	Objective Indicators【Laboratory examination（IL-4、IL-6、TNF-α)】	5	Effective Rate	6										
297	VAS	1	FMA	2	BI	3	ROM【Range of Motion of Shoulder Joint】	5												
298	NRS	1	ROM	5	MBI	3	Effective Rate	6												
299	VAS	1																		
300	VAS	1	FMA	2	Simple Evaluation of Upper Limb Function Score	2	Shoulder Joint Function Score	2												
301	Effective Rate	6	VAS	1	Melle Score	2	STEF Score	2	ADL	3	Hemorheology	5								
302	VAS	1	FM	2	FIM	2														
303	Effective Rate	6	VAS	1	GQOLI-74	3														
304	VAS	1	FMA	2	PROM	5	AROM	5	Effective Rate	6										
305	VAS	1	FMA	2	Ueda Assessment	2	BI	3												

					Scale for Hemiplegic Upper Limb															
306	VAS	1	FMA	2	Ueda Assessment Scale for Hemiplegic Upper Limb	2	BI	3	Effective Rate	6										
307	Frequency of pain	1	VAS	1	FMA	2														
308	Effective Rate	6																		
309	Effective Rate	6	VAS	1																
310	VAS	1	FMA	2																
311	VAS	1	FMA	2	ASES	1														
312	VAS	1	MAS	2	ROM	5	FMA	2	MBI	3										
313	VAS	1	FIQ	1	FMA	2	Effective Rate	6												
314	VAS	1	FMA	2																
315	VAS	1	Modified Lindmark Method	2	FMA	2														
316	VAS	1	FMA	2	ROM	5														
317	VAS	1	FMA	2	Ueda Assessment Scale for Hemiplegic Upper Limb	2	ROM	5	Effective Rate	6										
318	AFS	1	FMA	2	Stroke-Specific Quality of Life (SS-QOL) Score	3	Incidence of Shoulder-Hand Syndrome	1												
319	VAS	1	Headache score	1	Objective Indicators【Laboratory examination（Plasma pain-related factors,	5														

					endogenous opioid peptide content】															
320	VAS	1	CMS	1	FMA	2	Effective Rate	6	Adverse Event (AE)	7										
321	SHSS	1	Active Range of Motion (AROM) of the Shoulder Joint	2	FMA	2	Hemorheology【PV,WBV,PAgT,Fib）】	5	SF-MPQ	1										
322	VAS	1	Effective Rate	6	Safety and adverse effects	7	BI	3												
323	Clinical symptoms	1	Safety and adverse effects	7																
324	Effective Rate	6	VAS	1	ROM	5	UCLA	2	Musculoskeletal Ultrasound	5										
325	VAS	1	FMA	2	Effective Rate	6														
326	Effective Rate	6																		
327	Effective Rate	6	Safety Evaluation	7	Objective Indicators【Laboratory examination（SP,PGE2】	5	SAS	2	SDS	2	SF-36	3	GWB	10	GSES	10				
328	Quality of Life Questionnaire	3	Neer Score of Shoulder Joint	1	VAS	1														
329	VAS	1	FMA	2																
330	FMA	2	MBI	3	VAS	1	Effective Rate	6	Effective Rate	6	Safety and adverse	7								

											effects									
331	VAS	1	Muscle strength	2	FMA	2	MBI	3												
332	PSQI	2	VAS	1																
333	Effective Rate	6	VAS	1	FMA	2														
334	Effective Rate	6	VAS	1	PPI	1	PRI	1	Edema Severity	2	FMA-U	2	MAL-AOU	2	ADL	3	WHO-QOL	3		
335	VAS	1	ROM	5	FMA	2	QOL	3												
336	VAS	1	Effective Rate	6																
337	VAS	1	CMS	1	Effective Rate	6														
338	VAS	1	MSS	2	Brunnstrom	2	MBI	3												
339	FMA	2	VAS	1	BPI	1	ROM	5												

Note: Specific references are given at the end of Appendix 3.

VAS: Visual Analog Scale/Score; NRS: Numeric Rating Scale; NPRS: Numerical Pain Rating Index Scale; BRS: Behavioral Pain Scale; BPI: Brief Pain Inventory; PRI: Pain rating index; VRS: visual rating scale Intensities of pain; FIQ: Fibromyalgia Impact Questionnaire; NRS-FRS: Facial expression Rating Scale-Based Digital Rating Scale; FMA-S: Fugl-Meyer Scale of SensFunction; SFS: Sensory Function Assessment; reNSA: revised Nottingham Sensory Assessment Scale; SIS: Sensory Index Scale; BI: Barthel Index; MBI: Modified Barthel Index; ADL: activities of daily living; SF-36: Quality of Life-36; SS-QOL: Stroke-Specific Quality of Life; FIM: functional independence measure; FCA: Functional Capacity Assessment; WHO-QOL- BREF: World Health Organization Quality of Life Brief Scale; WHO-QOL: World Health Organization Quality of Life Scale ; GQOLI-74: General Quality of Life Unventory-74; GQOL-74: Generic quality of life inventory-74; SIS: stroke impact scale; QLQ-C30: European Organisation for Research and Treatment of Cancer Quality of Life Questionnaire C30; FAQ: functional activities questionnaire; QLI: Quality of Life Index; TAA: Task Analysis Assessment, BBSI: Behavioral Based Safety Index; SAS: Self-rating Anxiety Scale score; SDS:self-rating depression scale; HAMD: Hamilton Depression Rating Scale; HAMA: Hamilton Anxiety Scale; BAI: Beck Anxiety Inventory; SCL-90: Subscale of Symptom-Checklist; BDI : Beck Depression Inventory; ABC: Activities-specific Balance Confidence; WEMWBS: Warwick-Edinburgh Mental Well-being Scale; DASS-21: Depression, Anxiety and Stress Scales; HAD: Hospital Anxiety and Depression Scale; STAI: State-Trait Anxiety Inventory; GUSS: Gugging Swallowing Function Assessment Scale; BSA: Based on the bedside swallowing assessment; EAT-10: Eating Assessment Tool-10; VFSS: Videofluoroscopic Swallowing Study; SSA: Standardized Swallowing Assessment; MMSE: mini-mental state examination; MoCA: Montreal Cognitive Assessment scale; LOTCA: Loewenstein Occupational Therapy Cognitive Assessment; CDR: Clinical Dementia Rating Scale; HAMD: Hamilton Depression Scale; WAIS-III: Wechsler Adult Intelligence Scale, third edition; HDS: Hasegawa dementia scale; BDAE: The semantic score of the Boston Diagnostic Aphasia Examination; PSQI : Pittsburgh Sleep Quality Index; PRO: Patient-Reported Outcome Measures; SIAS: Stroke Impairment Assessment Set; OHS: Oxford Handicap Score; GEPI: GuidestotheEvaluationofPermanentImpairment ; AHD : Acromiohumeral Distance ; AGT : Acromion-GreaterTuberosity ; ALT : Acromion-LesserTuberosityALTNIHSS: National Institutes of Health Stroke Scale; NDS: Nerve Deficiency Scale;The CSS-1: Chinese Stroke Scale; mRS: modified Rankin scale; NFDS: Neural function defect score; MESSS: Modified Edinburgh-Scandinavian Stroke Scale; SSS: Scandinavian Stroke Scale; CCS: China clinical neurological deficit in stroke scale; ESS: European Stroke Scale; CNS: clinicalnervefunctionIim-itationscores; GOS: Glasgow Outcome Scale; CNDS: Clinical Neurological Disfunction Scale; CNFDS: Clinical neural function defect scale; NDF: The degree of neurological deficit score standard disability grade score; NBD: neurologicadeficit score; FMA: Fugl-Meyer Motor Assessment; FMMS: Fugl-Meyer assessment scale; SFMA: Selective Functional Movement Assessment; RMI: Rivermead Movement index; CMFM: Crude

Motor Function Scale; STREAM: the Stroke Rehabilitation Assessment of Movement; MSS, motor status score; COPM: Canadian Occupational Performance Measure; JOA: Japanese Orthopaedic Association Assessment Treatment Score; MAL: Motor Activity Log; MCT: Motor Control Test; FMA-UE: Fugl-Meyer assessment for the upper extremity; WMFT: Wolf Motor Function Test; DASH: DASH scale; STEF: Simple Test for Evaluating Hand Function; UEFT: Upper Extremity Function Test; The Ueda Senshiro Hemiplegic Function Evaluation Method; FTHUE-HK: the score of hemiplegic upper limb function test (Hong Kong version); MI: Upper extremity Motor Force Index (MI); SHSS: shoulder-hand syndrome scale; ARAT: Action Research Arm Test;FMA-F:Fugl-meyer assessment of finger; CMS: Constant-Murley Shoulder Function Scale; JTHFT: Jebsen Taylor Hand function test; SPADI: Shoulder Pain and Disability Index; UCLA: University of California Shoulder Score; ASES: American Association of Shoulder and Elbow Surgery score; MAL-AOU: Motor Activity Log Amount of Use; FMA-LE, Fugl–Meyer assessment of lower extremity subscore; AOFAS: ankle-hind foot function evaluation; IRISSG: International Restless Leg Syndrome Study Group Classification; MI-L: lower extremity muscle strength of Motricity index; BBS: Berg Balance Scale; FMA-B: The Fugl-Meyer Assessment Scale of Balance Functional;ICARS: International Cooperative Ataxia Rating Scale; PASS: Postural Assessment Scale; TCT: Trunk Control Test ; TIS: Trunk Dysfunction Scale; BBA: Brunel Balanced Assessment; BLS: Burke Lateropulsion Scale; mini BESTest: Mini Balance Evaluation Systems Test; SCP: Scale for Contraversive Pushing; MCT: Motor Control Test; CRT: Stand-up tes; PST: Postural stability test; EMS: Elderly Mobility Scale; MRMI: United Kingdom Medical Research Council MRMI Scale; POMA: Performance-Oriented Mobility Assessment scale; HFAC: Holden Functional Ambulation Category; FAC: Functional Ambulation Category; TUG: The Timed Up & Go; TGA: Tinetti Gait Assessment Scale; FGA: Functional Gait Evaluation Scale; WGS: Wisconsin Gait Scale; MAS：Modified Ashworth Scale； CSI: composite spasticity index；CSS：composite spasticity score；MTS：Modified Tardieu Scale

Appendix 15.Risk assessment of RCTs.

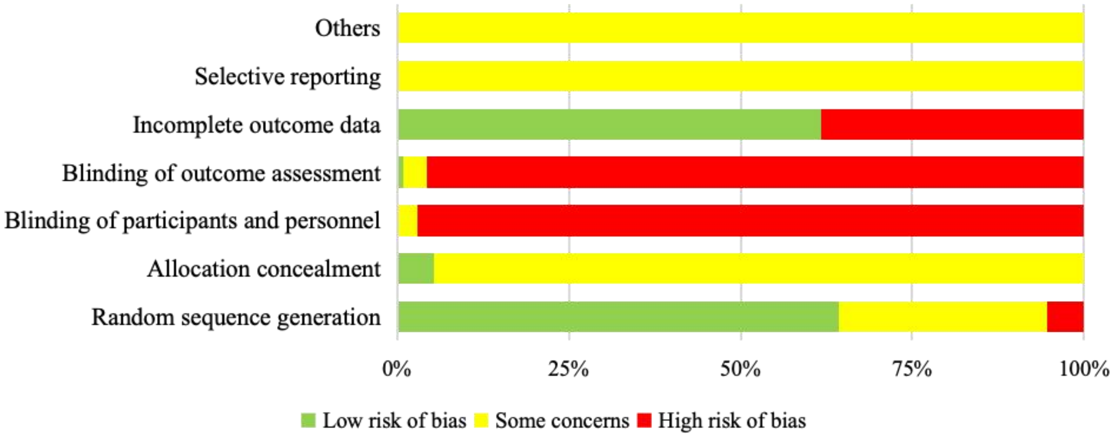

Appendix 16.Basic information and quality assessment of SRs/MAs.

Basic information for inclusion in SRs/MAs

Ref. No.	Types of included studies	Number of included literatures	Total sample size	Quality assessment tools	Control	Intervention	Outcome measures	Conclusion	Categories of Conclusions
346	RCT	29	2250	the Joanna Briggs Institute (JBI) Critical Appraisal Checklist	Routine stroke care	AC	shoulder pain、upper extremity function、edema、shoulder ROM、physical function、health-related quality of life、depression、self-esteem、and adverse events related to the acupuncture therapies received.	Although most studies reviewed concluded that conventional and elec troacupuncture could be effective for management of shoulder pain after stroke, the very high potential for bias should be considered. Further work in this area is needed that employs standardized acupuncture treatment modalities, endpoint assessments, and blinding of treatments.	Unclear
345	RCT	12	1002	ROB	RT	AC+RT	VA、FMA、Effective rate、FMA-UE、	Although there is someevidence for an effect of acupuncture on poststroke shoulder pain, the results are inconclusive. Further studies with more subjectsand a rigorous study design are needed to confirm the role of acupuncture in the treatment of poststroke shoulder pain.	Unclear
344	RCT	**15**	978	ROB	RT	AC	VAS、MBI、AEs	Acupuncture is similar to RT in relieving shoulder pain, improving upper limb motor function and ADL in patients with PSSP. Either acupuncture or RT might be the optimal treatment of PSSP. More well-designed RCTs of this topic are needed in the future.	Effective
343	RCT	40	2554	ROB	RT	RT+AC	VAS、NRS、MPQ/FPS、FMA-U、ADL、ROM、AEs	AR may be better than RT alone for the improvement of shoulderpain, upper limb motor function, ADL, and shoulder ROM, without obviousAEs in patients with PSSP. However, considering the clinical and statisticalheterogeneity, our findings need to be interpreted with caution. More rigorousRCTs in this area should be conducted in the future.	Effective
342	RCT	10	630	ROB	Blank /Placebo	Blank/Placebo+Wrist-ankle needle	VAS、Effective rate、AROM、FMA、U-FMA、BI、	The results of this study show that wrist-ankle acupuncture has a certain effect in the treatment of post-stroke hemiplegic shoulder pain, which can effectively reduce the degree of shoulder pain, improve the range of shoulder abduction and the ability of activities of daily living in patients with post-stroke hemiplegic shoulder pain. However, in the aspect of upper limb motor function, there is no difference between the wrist ankle acupuncture and the control group. The above conclusions need to be confirmed by more rigorous and high-quality studies.	Effective
341	RCT	18	1405	ROB	Others	Floating needle/Floating needle+Others	VAS、 FMA、Shoulder- hand syndrome scale, SHSS、Effective rate	Fu's Fu's acupuncture in the treatment of post-stroke shoulder pain can effectively improve the upper limb function and pain symptoms of patients, and improve the quality of life. However, due to the generally low quality of the literature, this conclusion still needs to be treated with caution, and more multi-center, large -sample, high-quality randomized controlled trials are needed to support and confirm it.	Effective
340	RCT	14	1189	ROB	Others	AC	**Effective rate、VAS**、MBI	Acupuncture has a definite effect on SHS after stroke, which is superior to the conventional treatment. However, the level of evidence is low, and large-sample, high-quality and more rigorously designed randomized controlled trials are needed to further verify the results.	Potentially effective

Quality assessment of SRs/MAs was included

Ref. No.	item 1	item 2	item 3	item 4	item 5	item 6	item 7	item 8	item 9	item 10	item 11	item 12	item 13	item 14	item 15	item 16	Overall quality
346	Y	Y	N	P-Y	Y	Y	P-Y	P-Y	Y	N	Y	Y	Y	Y	Y	N	Moderate
345	Y	N	N	P-Y	Y	Y	P-Y	P-Y	Y	N	Y	Y	N	N	N	Y	Critically low
344	Y	Y	N	P-Y	Y	Y	P-Y	P-Y	Y	N	Y	Y	Y	Y	Y	Y	High
343	Y	Y	N	Y	Y	Y	Y	P-Y	Y	N	Y	Y	Y	Y	Y	Y	High
342	Y	N	N	P-Y	Y	Y	P-Y	P-Y	Y	N	Y	Y	Y	Y	Y	N	Critically low
341	Y	N	N	P-Y	Y	Y	P-Y	P-Y	Y	N	Y	Y	Y	Y	Y	N	Critically low
340	Y	N	N	P-Y	Y	Y	P-Y	Y	Y	N	Y	Y	Y	Y	N	N	Critically low

【Reference】

340 Du Yuanhao, Zhang Man, Yin Xiumei, Di Jiawei, Ye Yanzhen. Meta-analysis and GRADE evidence rating of acupuncture for shoulder hand syndrome after stroke. Chinese folk therapy. 2023;31(5).

341 Wei Zemin, Sima Xunxin, Wu Qiang, Gong Jianqiu. To systematically evaluate the clinical efficacy of floating needle therapy for poststroke shoulder pain. New traditional Chinese medicine. 2019(10).

342 Qiu Zhiqin, Yu Mengting, Li Zhuangmiao, Yan Wenjuan, Zhang Jiayu,. Meta-analysis of wrist and ankle injection for hemiplegic shoulder pain in stroke. TCM clinical research. 2022;14(15).

343 Zhan J, Wei X, Tao C, Yan X, Zhang P, Chen R, et al. Effectiveness of acupuncture combined with rehabilitation training vs. rehabilitation training alone for post-stroke shoulder pain: A systematic review and meta-analysis of randomized controlled trials. Frontiers in medicine. 2022;9:947285.

344 Zhan J, Luo Y, Mao W, Zhu L, Xu F, Wang Y, et al. Efficacy of acupuncture versus rehabilitation therapy on post-stroke shoulder pain: A systematic review and meta-analysis of randomized controlled trials. Medicine. 2023;102(29):e34266.

345 Lee SH, Lim SM. Acupuncture for Poststroke Shoulder Pain: A Systematic Review and Meta-Analysis. Evidence-based complementary and alternative medicine : eCAM. 2016;2016:3549878.

346 Chau JPC, Lo SHS, Yu X, Choi KC, Lau AYL, Wu JCY, et al. Effects of Acupuncture on the Recovery Outcomes of Stroke Survivors with Shoulder Pain: A Systematic Review. Frontiers in neurology. 2018;9:30.
